# First randomised controlled trial comparing the sirolimus-eluting bioadaptor with the zotarolimus-eluting drug-eluting stent in patients with *de novo* coronary artery lesions: 12-month clinical and imaging data from the multi-centre, international, BIODAPTOR-RCT

**DOI:** 10.1016/j.eclinm.2023.102304

**Published:** 2023-10-24

**Authors:** Shigeru Saito, Johan Bennett, Holger M. Nef, Mark Webster, Atsuo Namiki, Akihiko Takahashi, Tsunekazu Kakuta, Seiji Yamazaki, Yoshisato Shibata, Douglas Scott, Mathias Vrolix, Madhav Menon, Helge Möllmann, Nikos Werner, Antoinette Neylon, Zlatko Mehmedbegovic, Pieter C. Smits, Marie-Claude Morice, Stefan Verheye, BIOADAPTOR-RCT Collaborators

**Affiliations:** a Heart Center, Iryohojin Tokushukai Shonan Kamakura General Hospital, Kamakura City, Japan; b Department of Cardiovascular Medicine, UZ Leuven, Leuven, Belgium; c Department of Cardiology and Angiology, University of Giessen, Giessen, Germany; d Cardiac Investigation Unit, Auckland City Hospital, Auckland, New Zealand; e Department of Cardiology, Kanto Rosai Hospital, Nakahara-ku, Kawasaki-shi, Japan; f Department of Cardiology, Takahashi Hospital, Kobe City, Japan; g Cardiovascular Medicine, Tsuchiura Kyodo Hospital, Tsuchiura City, Japan; h Department of Cardiology, Sapporo Higashi Tokushukai Hospital, Sapporo, Japan; i Department of Cardiology, Miyazaki Medical Association Hospital, Miyazaki City, Japan; j Department of Cardiology, Middlemore Hospital, Auckland, New Zealand; k Department of Cardiology, Ziekenhuis Oost-Limburg, Campus Sint Jan, Genk, Belgium; l Department of Cardiology, Waikato Hospital, Hamilton, New Zealand; m Department of Cardiology, St. Johannes Hospital Dortmund, Dortmund, Germany; n Department of Cardiology, Krankenhaus der Barmherzigen Brüder Trier, Trier, Germany; o CERC (Cardiovascular European Research Center) ICPS Ramsay, Massy, France; p Interventional Cardiology, ZNA Cardiovascular Center Middelheim, Antwerp, Belgium

**Keywords:** Bioadaptor, Percutaneous coronary intervention, Coronary artery disease, Drug-eluting stents, Cyclic pulsatility, Vessel function

## Abstract

**Background:**

The DynamX™ bioadaptor is the first coronary implant technology with a unique mechanism of unlocking the bioadaptor frame after polymer resorption over 6 months, uncaging the vessel while maintaining a dynamic support to the vessel. It aims to achieve the acute performance of drug-eluting stents (DES) with the advantages of restoration of vessel function.

**Methods:**

This international, single blinded, randomised controlled (1:1) trial compared a sirolimus-eluting bioadaptor with a contemporary zotarolimus-eluting stent (DES) in 34 hospitals in Europe, Japan and New Zealand. Patients with *de novo* coronary lesions and absence of acute myocardial infarction were enrolled from January 2021 to Feburary 2022. The implantation of the bioadaptor followed the standards of DES. An imaging subset of 100 patients had angiographic and intravascular ultrasound assessments, and 20 patients additionally optical coherence tomography. Data collection will continue through 5 years, we herein report 12-month data based on an intention-to-treat population. This trial is registered at ClinicalTrials.gov (NCT04192747).

**Findings:**

445 patients were randomised between January 2021 and February 2022. Device, lesion and procedural success rates, and acute gain were similar amongst the groups. The primary endpoint, 12-month target lesion failure, was 1.8% [95% CI: 0.5; 4.6] (n = 4) versus 2.8% [95% CI: 1.0; 6.0] (n = 6), p_non-inferiority_ < 0.001 for the bioadaptor and the DES, respectively (Δ-1.0% [95% CI: −3.3; 1.4]). One definite or probable device thrombosis occurred in each group. The 12-month imaging endpoints showed superior effectiveness of the bioadaptor such as in-device late lumen loss (0.09 mm [SD 0.34] versus 0.25 mm [SD 0.39], p = 0.04), and restored compliance and cyclic pulsatility (%mid in-device lumen area change of 7.5% versus 2.7%, p < 0.001).

**Interpretation:**

This is the first randomised controlled trial comparing the novel bioadaptor technology against a contemporary DES. The bioadaptor demonstrated similar acute performance and 12-month clinical outcomes, and superior imaging endpoints including restoration of vessel function.

**Funding:**

The study was funded by Elixir Medical.

**Research in Context**

**Evidence before this study**

To identify all publications using the DynamX bioadaptor, the search term (Dynamx OR bioadaptor) AND coronary was entered into the PubMed database on May 23rd, 2023, and to identify publications referring to cyclic pulsatility, the following search term was entered: (coronary artery [Title/Abstract]) AND (cyclic pulsatility). Four publications were identified for DynamX of which one was excluded as it was a duplicate, referring to the DynamX mechanistic trial as Key Clinical Trial in 2020, the remaining three articles referred to 12- and 24-month data of the DynamX mechanistic trial and one preclinical study; for cyclic pulsatility, the search revealed 14 hits, of which two were used (one book chapter on angioplasty and one preclinical model to study coronary stent fractures), six were excluded as they referred to different indications and six were excluded as they did not refer to cyclic pulsatility itself. Considering that the identified articles refer to single-arm studies, preclinical studies and reviews, there is a certain risk of bias.

**Added value of this study**

This is the first randomised study comparing a drug-eluting bioadaptor with a drug-eluting stent (DES). It demonstrated that a percutaneous coronary intervention (PCI)-procedure has non-inferior procedural outcomes and 12-month target lesion failure, as well as superior late lumen loss and vessel motion (cyclic pulsatility) compared to a contemporary DES.

**Implications of all the available evidence**

The outcomes of this randomised controlled trial demonstrate that the implantation of the DynamX sirolimus-eluting bioadaptor is associated with favorable safety and performance outcomes and give promise for establishing a new effectiveness standard over DES.

## Introduction

Around 40 years ago, the first PCIs were performed, using uncoated balloons to dilate the stenotic parts of the coronary vessels. The treatment was limited by vascular recoil and high restenosis rates, leading to the development of bare metal stents. While restenosis rates improved, they were still high, resulting in the development of drug-eluting stents (DES) that substantially improved outcomes, particularly newer generations with improved biocompatibility.^1^

Nonetheless, events continue to occur over time,^1^^,^^2^ impacted by the permanent vessel caging that constrains, distorts, and stretches the vessel, thereby inhibiting vasomotion and positive remodeling.^1^^,^^3^, ^4^, ^5^, ^6^ A landmark analysis after one year in more than 25,000 patients demonstrated that there was no improvement of stent technologies in terms of clinical events beyond one year, with target lesion failure rates that were similar for bare metal stents and second-generation DES, calling for new approaches to improve long-term outcomes.^2^

Bioresorbable scaffolds (BRS) were developed to free the coronary artery from metal caging, to restore cyclic pulsatility, ultimately preventing clinical events and to improve the effectiveness of PCI beyond DES. However, BRS suffered from several shortcomings such as deliverability challenges due to thick scaffold struts, inferior acute performance (e.g., suboptimal acute gain),^1^ and first generation devices have been associated with higher target lesion failure (TLF) and device thrombosis rates compared to DES.^1^^,^^6^ Newer generation BRS hold some improvements, but data are limited to small single-arm studies so far.^7^

**Fig. 1:**
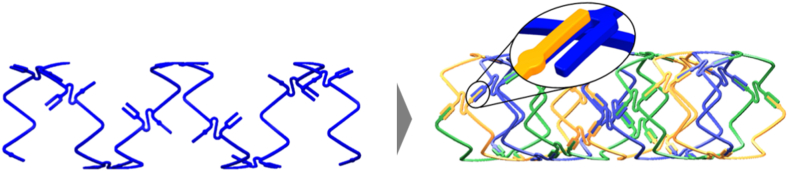
**DynamX bioadaptor.** The DynamX bioadaptor is comprised of three cobalt-chromium helical strands (denoted in blue, green, yellow) that are temporarily linked together by unique uncaging elements and bioresorbable polymer coating. The polymer coating resorbs over six months and enables the separation of the helical strands from each other (small image). Used with the permission of Elixir Medical Corporation.

The novel DynamX™ sirolimus-eluting bioadaptor (Elixir Medical Corporation, Milpitas, CA) is designed to address the aforementioned shortcomings of current PCI-devices. It aims to be similar to DES in terms of acute clinical performance, and superior in terms of effectiveness, including restoration of vessel motion and function. As an adaptive implant, initially the bioadaptor acts like a DES and can be implanted like a contemporary stent, but the device also has bioresorbable properties to transform the device and “uncage” the vessel. It is made of three 71 μm cobalt-chromium (CoCr) sinusoid helical strands that are joined radially by three unlocking (uncaging) elements per ring along the length of the device, and are held together by a bioresorbable polymer coating (basecoat). The thin polymer coating resorbs over six months allowing the CoCr helical strands to initiate the mechanism of “unlocking” the bioadaptor and allowing the helical strands to separate, uncaging the artery to restore motion and function, and continuing to provide dynamic support of the diseased vessel while adapting to vessel biomechanical forces. With this, the bioadaptor shall also be forearmed against device fractures (Fig. 1, Supplemental Movies 1 and 2).^8^

As demonstrated in the first clinical study with this device, after the release of the uncaging elements, the device is no longer a rigid structure and enables restoration of vessel function as shown by a) improved vessel cyclic pulsatility in response to the cardiac cycle, b) improved compliance, c) response to pharmacological agents, d) ability of positive arterial remodeling and e) restored coronary rotational motion.^9^, ^10^, ^11^ Subsequently, in the 50 patients implanted, there were no device or procedure related cardiac deaths, target-vessel myocardial infarction (TV-MI) or definite or probable device thrombosis up to 3 years of follow-up, and only one clinically-driven target lesion revascularization (CD-TLR).^12^

Based on these promising outcomes, a pivotal randomised controlled trial (RCT) was initiated to compare outcomes against a contemporary thin-strut DES, the Resolute Onyx DES (Medtronic, Santa Rosa, CA), which has demonstrated non-inferiority to other contemporary ultrathin strut DES.^13^ We herein report the primary endpoint, 12-month TLF to confirm non-inferiority to the current standard of care. Furthermore, imaging assessments at 12 months were performed in patient subsets to establish material and biomechanical effectiveness and the restoration of vessel motion and function after the unlocking of the bioadaptor. These are the first published randomised data for this novel technology.

## Methods

### Study design

The study design has been described previously.^14^ In brief, BIOADAPTOR-RCT is a multi-centre, international, randomised (1:1), single-blinded non-inferiorirty study conducted in Japan (14 centres), Europe (14 centres) and New Zealand (six centres). The study was stratified by region and was scheduled to enroll at least 222 patients per region (Japan and Europe/New Zealand). A pharmacokinetic substudy, performed in 8 patients receiving the DynamX sirolimus-eluting bioadaptor enrolled in Japan to assess the pharmacokinetics of sirolimus will be separately reported.

Source document verification is performed for all patients, a Clinical Events Committee adjudicates all endpoint-related clinical events, and an independent Core laboratory (CERC) analyses the imaging outcomes; the Clinical Events Committee and Core laboratory assessors are blinded to the treatment.

The clinical investigation plan is accessible as Appendix 2.

### Ethics

The study is conducted according to the Declaration of Helsinki, ISO14155, Good Clinical Practice, and local and national regulation, and was approved by the independent ethics committee of each participating center and the respective regulatory authorities: Tokushukai Group Joint IRB, Chiyoda-ku, Tokyo (Ref# 012-19-07 and 024-19-13), Society for Ethics in Clinical Research, Chuo-ku, Tokyo (Ref# A226), Cardiovascular Institute Hospital IRB, Minato-ku, Tokyo (Ref# ELX-CL-1805), Kanto Rosai Hospital IRB, Kawasaki-shi, Kanawaga (Ref# ELX-CL-1805), Yokohama City Eastern Hospital IRB, Yokohama City, Kanagawa (Ref# B-47), Oumi Hachiman City General Medical Center IRB, Omihachiman City, Shiga (Ref# ELX-CL-1805), Shinkoga Hospital IRB, Kurume-shi, Fukuoka (Ref# ELX-CL-1805), Kokura Memorial Hospital IRB, Kitakyushu-shi, Fukuoka (Ref# ELX-CL-1805), Miyazaki Medical Association Hospital IRB, Miyazaki City, Miyazaki (Ref# ELX-CL-1805), Tenyokai Central Hospital IRB, Kagoshima City (Ref# ELX-CL-1805), Takahashi Hospital IRB, Kobe, Hyogo (Ref# ELX-CL-1805), Kumamoto Rousai Hospital IRB. Yatsushiro City, Kumamoto (Ref# 2-1), Teikyo Univ Hospital IRB, Itabashi-ku, Tokyo (Ref# 20-382); Pharmaceuticals and Medical Devices Agency (PMDA, Ref# ELX 1805J); lead ethics committee Belgium: Ethics Committee Research UZ/KU Leuven (Ref# S63367); local ethics committees: Commissie voor Medische Ethiek ZNA, Antwerp (Ref# 5305), Ziekenhuis Oost-Limburg, Campus Sint Jan, Genk (Ref 19/0086L), AZ Sint Jan Brugge Oostende AV (Ref#2847); Federal Agency for Medicines and Health Products (Ref# FAGG/80/0800); lead ethics committee Germany: Ethik-Kommission des Fachbereichs Medizin der Justus-Liebig-Universität Gieβen (Ref# 24/20); local ethic committees: Ethikkommission der Landesärztekammer Rheinland-Pfalz, Mainz (Ref# 2021-15812), Geschäftsstelle der Ethikkommissionen bei der Ärztekammer Schleswig–Holstein, Bad Segeberg (Ref# 123/21 m), Ethik-Kommission bei der Landesärztekammer Hessen, Frankfurt (Ref# V/1/ewa – 2021-2461-zvBO), Friedrich-Alexander-Universität Ethik-Kommission, Erlangen (Ref# 90_21 Bc); Ethik-Kommission Westfalen-Lippe, Münster (Ref#2021-272-b-S), Ethikkommission der Friedrich-Schiller-Universität Jena (Ref 2021-2185_1 MPG §23), Medizinische Fakultät der Christian-Albrechts-Universität zu Kiel (Ref# B 240/21); Central ethics committee New Zealand: Health and Disability Ethics Committees, Ministry of Health, Northern A HDEC (Ref # 19/NTA/159); Medsafe notification (Ref # 19/NTA/159). All patients signed their informed consent before any study-specific procedure.

### Participants

All patients scheduled for PCI had to be screened for eligibility. Main inclusion criteria were: *de novo* coronary lesions with a vessel mean diameter of ≥2.25 and ≤4.0 mm, visually estimated stenosis of ≥50% and <100%, visually estimated target lesion length ≤34 mm, and when two target lesions were treated, they were to be located in separate major epicardial vessels. Main exclusion criteria were: acute myocardial infarction within 72 h prior to enrollment (and the CK and CK-MB have not returned to normal, or cTn >15x ULN, and the patient is experiencing clinical symptoms indicative of ongoing ischemia), lesion located in the left main, patients with dissection of Grade A or B that could not be covered (including 2 mm distal to the dissection) with a single study device or with dissection of Grade C or higher. The full list of inclusion/exclusion criteria has been published^14^ and is available from ClinicalTrials.gov (NCT04192747).

### Randomisation and masking

Randomisation was performed after successful pre-dilatation of the target lesion using an Interactive Web Response System with block randomization (1:1) and random block sizes per site, and was stratified by study site. It was performed by the personnel responsible for allocation and enrollment at the site and was independent of the sponsor and medical institution.

The study personnel could not be blinded due to the different device shapes. Furthermore, biostatisticians were not masked to the treatment group. The patients, the Core laboratory, and the Clinical Events Committee were blinded to the treatment.

### Interventions

The DynamX sirolimus-eluting bioadaptor (Fig. 1, Supplemental Movies 1 and 2) consists of three 71 μm cobalt-chromium helical strands acting as a backbone coated with two bioresorbable polymer layers, a base coat that holds the helical strands together and a top coat that elutes sirolimus. Specifically, the bioresorbable poly-lactic-co-glycolic acid top coat elutes sirolimus (approximately 7 μg per mm of device length) over three months. The 6 μm poly-L-Lactic-acid based polymer base coat that temporarily holds the bioadaptor together resorbs over 6 months, releasing the axial connection of the helical strands, ultimately leaving the separated CoCr longitudinally connected helical strands surrounded by smooth muscle cells and embedded in the vessel wall. The comparator, the Resolute Onyx DES, consists of a stent platform made of a cobalt-nickel alloy with platinum core that is coated with a durable polymer incorporating zotarolimus, a ‘limus’ analogue.

Pre-dilatation was mandatory in both arms and the residual diameter stenosis was to be <30% prior to randomisation; post-dilatation was left to the operator's discretion.

Per guidelines,^15^ dual antiplatelet therapy was recommended for a minimum of 6 months in stable patients, and for a minimum of 12 months in patients with acute coronary syndrome, unless contraindicated.

Clinical follow-up was scheduled at 1, 6, and 12 months, and will be repeated annually thereafter for a total of 5 years. A subset of 100 patients enrolled in Japan was scheduled to receive additional angiographic and intravascular ultrasound (IVUS) follow-up at 12 months; thereof 20 patients to receive additional optical coherence tomography (OCT) assessments. We herein report 12-month clinical and imaging data.

The angiographic assessment was to be performed in at least two matched orthogonal projections before and after the intervention, and at follow-up. In the imaging subsets, IVUS was to be performed with a motorized pullback of 0.5–1 mm/s, using the same speed at post-procedure and follow-up. The same frame were to be employed for all positions at post-implant and follow-up. For stationary recording, a minimum of three cardiac cycles were to be recorded using the same frames at post-procedure and follow-up. For OCT, the same imaging sequences were to be used. For quantitative angiographic analysis, the software QAngio XA 7.3 (Medis medical imaging systems B.V.) was used, for IVUS and OCT analysis CAAS IntraVascular 2.0 (Pie Medical Imaging B.V.).

### Outcomes

Endpoints have been described in detail previously.^14^ The primary endpoint is TLF at 12 months, defined as a composite of cardiovascular death, TV-MI and clinically-driven TLR. Secondary endpoints include lesion success (% diameter stenosis after treatment of target lesion with percutaneous coronary intervention, PCI, <30%), device success (% diameter stenosis after implantation of allocated study device <30%), and procedure success (lesion success without major adverse cardiac events during index hospitalisation). Further secondary endpoints are the individual composites of TLF; all-cause mortality; myocardial infarction (MI)^16^^,^^17^; target vessel revascularization (TVR); non-target vessel revascularization; definite or probable device thrombosis; stroke; patient oriented composite,^16^ and composites of all-cause mortality, TV-MI, revascularization, of cardiovascular death, TV-MI and clinically-driven TVR, of cardiovascular death, stroke, MI and revascularization, of cardiovascular death, MI and revascularization, of cardiovascular death and TV-MI, of all-cause death and MI, and of all-cause death, MI and TVR. Angiographic endpoints are acute recoil; late lumen loss (LLL); % diameter stenosis at post-procedure and follow-up; and change in vessel angulation; Intravascular imaging endpoints are stent malapposition (defined as 200 μm separation from the lumen), % strut coverage, neointimal hyperplasia (NIH) thickness, and vessel pulsatility (change in lumen area during cardiac cycle, calculated as the change between the maximal lumen area and minimal lumen area during the cardiac cycle, calculated as [Max-La − Min-La]/Max-La × 100%). For blood flow changes during the cardiac cycle the Hagen Poiseuille flow equation was used, assuming that blood is Newtonian, incompressible, and flow is laminar, fully developed; blood flow change was estimated to be proportional to the change in arterial radius taken to the fourth power (or lumen area taken to the second power), indicating its high sensitivity to changes in vessel radius and area.^18^

### Statistics

The sample size is based on non-inferiority of 12-month TLF compared to the Resolute Onyx DES and has been reported in detail previously.^14^ In brief, on the basis of Pharmaceutical Device Agency (PMDA) guidance and in accordance with the sample size calculation of another device that aimed PMDA approval,^19^ a minimum of 400 patients were calculated for a power of 90%, assuming a 12-month TLF-rate of 9%, a non-inferiority margin of 8.6%, and a one-sided α of 0.05. 44 patients were added to a total of 444 patients to allow for a 10% drop-out rate. The 100 patients for the pre-specified imaging subgroup analysis were based on discussions with regulatory authorities, and is sufficient to detect relevant statistical differences with adequate statistical power. In particular, assuming a late lumen loss of 0.12 mm in the DynamX group and 0.22 mm in the control group with a standard deviation of 0.18 mm,^10^^,^^20^ and a one-sided 5% level of significance with equal allocation, a sample size of 100 has approximately 87% power to demonstrate statistical difference between the two arms. In terms of pulsatility, assuming a pulsatility of 10% in the DynamX group (similar to 8–10% seen in native coronary arteries^21^^,^^22^) and 5% in the control group (halved due to caging effect) with a standard deviation of 10%, and a one-sided 5% level of significance with equal allocation, a sample size of 100 has approximately 80% power to demonstrate statistical difference between the two arms.

The primary analysis is based on the intention-to-treat population (patients were included in the analysis whether they fulfilled the in- and exclusion criteria or not) and based on the data available. Continuous data are presented as mean and standard deviation or median and interquartile ranges (IQR). Categorical data are presented as frequency and percentages. Confidence intervals are presented as appropriate. In addition, Kaplan–Meier estimates were calculated for the primary endpoint. Time zero was defined as the day of the procedure and extended until the earliest of an adjudicated event, death, study discontinuation, or 360 days post procedure.

Kaplan–Meier estimates are compared using the log-rank test, categorical variables using the Chi-square test whenever possible. When the assumptions of the Chi-square test were not met, the Fisher's exact test was used instead. For continuous variables, Gaussian distribution of the data was tested using the Kolmogorov–Smirnov test. Normally distributed continuous variables were compared using the two tailed t-test for independent samples to compare the means across groups with Welch correction; the Wilcoxon-Mann-Whitney U-test was used if the data was not normally distributed.

The following exploratory analyses were performed for a more detailed view on the data: Subgroup analyses for in-device LLL and % diameter stenosis for left anterior descending (LAD) lesions, small vessels (≤2.75 mm) and long lesions (≥23 mm) were performed. Lesions were characterized as lipid containing, fibrotic, calcified, or a combination of the same during IVUS analyses. A subgroup analysis was performed for change in plaque volume for lipid containing lesions and all lesions except calcified lesions. Statistical analyses were performed using the SAS® System, version 9.4 (SAS Institute Inc, Cary, NC). For imaging analyses, GraphPad Prism version 9.5.1 for Windows (GraphPad Software, San Diego, CA) was used.

### Role of the funding source

The study is funded by Elixir Medical. The study sponsor was involved in the trial design, in collection of the data, in the data analysis, and reimbursed the cost of the medical writer and open access charges.

## Results

### Baseline characteristics

**Fig. 2:**
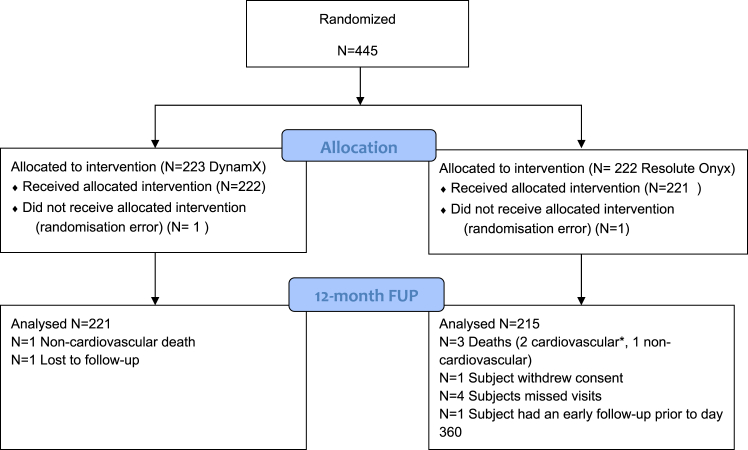
**Study Flow Chart.** ∗As cardiovascular death is an endpoint, these two patients count into the analysis.

From January 2021 to February 2022, 445 patients were randomly assigned to either the DynamX sirolimus-eluting bioadaptor or the Resolute Onyx stent (Fig. 2). One patient in each group did not receive the allocated therapy due to a randomisation error. The baseline and lesion characteristics were similar in both groups and are summarised in Table 1.

**Table 1:** Baseline characteristics.

	DynamX (N = 223)	Resolute Onyx (N = 222)
Age (years)	67.1 (10.3)	66.2 (10.1)
Sex		
Male	174 (78.0%)	173 (77.9%)
Female	49 (22.0%)	49 (22.1%)
Race		
Japanese	110 (49.3%)	113 (50.9%)
Caucasian	94 (42.2%)	89 (40.1%)
Maori	2 (0.9%)	1 (0.5%)
Not permitted to collect	12 (5.4%)	15 (6.8%)
Other	5 (2.2%)	4 (1.8%)
Smoking status		
Current	52 (23.6%)	48 (21.8%)
Former	96 (43.6%)	96 (43.6%)
Diabetes mellitus	59 (26.5%)	75 (33.8%)
Dyslipidemia	178 (80.9%)	177 (80.5%)
Hypertension	161 (73.2%)	156 (70.9%)
Cerebrovascular disease (stroke, TIA)	18 (8.2%)	12 (5.5%)
Renal Insufficiency	10 (4.5%)	13 (5.9%)
Peripheral Vascular Disease	18 (8.2%)	15 (6.9%)
Previous myocardial infarction	42 (19.1%)	48 (21.8%)
Previous CABG	2 (0.9%)	1 (0.5%)
Previous PCI	88 (40.0%)	83 (37.7%)
Angina/ischemia status		
Stable angina	144 (64.6%)	150 (67.6%)
Unstable angina	16 (7.2%)	9 (4.1%)
Silent ischemia	18 (8.1%)	18 (8.1%)
Asymptomatic post myocardial infarction	6 (2.7%)	15 (6.8%)
Non-ST-elevation myocardial infarction	15 (6.7%)	10 (4.5%)
Other	24 (10.8%)	20 (9.0%)

Lesion characteristics	DynamX (N = 226 lesions)	Resolute Onyx (N = 230 lesions)
Target lesion classification		
B2/C	51 (22.6%)	49 (21.3%)
Target vessel		
LAD	114 (50.4%)	104 (45.2%)
LCX	35 (15.5%)	66 (28.7%)
RCA	77 (34.1%)	60 (26.1%)
Bifurcation	50 (22.1%)	55 (23.9%)
Calcified lesion (moderate/severe)	43 (19.0%)	47 (20.4%)
Tortuous lesion (moderate/severe)	53 (23.5%)	46 (20.0%)
Reference vessel diameter (mm)	3.1 (0.4)	3.0 (0.4)
Target lesion length (mm)	15.8 (6.0)	16.2 (6.0)

Data are displayed as mean (SD) or n (%).

CABG = coronary artery bypass graft, LAD = left anterior descending, LCX = left circumflex, PCI = percutaneous coronary intervention, RCA = right coronary artery, TIA = transient ischemic attack.

### Procedural characteristics

Procedural characteristics are presented in Supplemental Table 1. The mean device length was 21.7 mm (SD 6.0) for DynamX and 21.9 mm (SD 6.4) for Resolute Onyx. Device, lesion, and procedural success were 99.6% (224/225), 99.6% (225/226) and 98.7% (220/223) versus 99.6% (228/229), 99.6% (229/230) and 97.3% (216/222), respectively. The use of antiplatelet and lipid lowering drugs was similar amongst the groups and is listed in Supplemental Table 2.

### Clinical follow-up

At 12 months, DynamX was non-inferior to Resolute Onyx in terms of TLF (1.8% [n = 4, 95% CI: 0.5; 4.6] versus 2.8% [n = 6, 95% CI: 1.0; 6.0], p_non-inferiority_ < 0.001 (Table 2 and Supplemental Fig. 1); there was also no difference in the individual components of TLF. Two out of the four TLF in the DynamX group were in patients who violated the exclusion criteria while all TLF events in the Resolute Onyx group occurred in per-protocol patients (for further details, see Supplemental Table 3). Definite or probable device thrombosis was observed in one patient of each group. The full list of secondary clinical outcomes is provided in Supplemental Table 4.

**Table 2:** Target lesion failure and device thrombosis at 12 months.

	DynamX (N = 223)	Resolute Onyx (N = 222)	Difference (%)	CI of difference	p_non-inferiority_^a^
Target lesion failure	4/221 (1.8%) [0.5; 4.6]	6/215 (2.8%) [1.0; 5.6]	−1.0%	[−3.3; 1.4]	<0.001
Cardiovascular death	0/221 (0.0%) [0.0; 1.7]	2/215 (0.9%) [0.1; 3.3]	−0.9%	[−3.4; 0.8]	–
Target vessel MI^b^	3/221 (1.4%) [0.3; 3.9]	4/213 (1.9%) [0.5; 4.7]	−0.5%	[−3.6; 2.3]	–
Clinically-driven TLR	2/221 (0.9%) [0.1; 3.2]	1/213 (0.5%) [0.0; 2.6]	0.4%	[−1.8; 2.9]	–
Definite or probable device thrombosis^c^	1/221 (0.5%) [0.0; 2.5]	1/214 (0.5%) [0.0; 2.6]	−0.0%	[−2.2; 2.2]	–

Data are displayed as n/N (%) [95% CI].

MI = myocardial infarction, TLR = target lesion revascularisation.

a Wald non-inferiority test statistic.

b Two periprocedural MI in the DynamX group and four in the Resolute Onyx group.

c The device thrombosis in the DynamX group occurred on day 3 in the patient with spontaneous target-vessel MI who was treated with a clinically-driven TLR; the device thrombosis in the Resolute Onyx group occurred in a patient with sudden death at day 1.

### Imaging follow-up

**Fig. 3:**
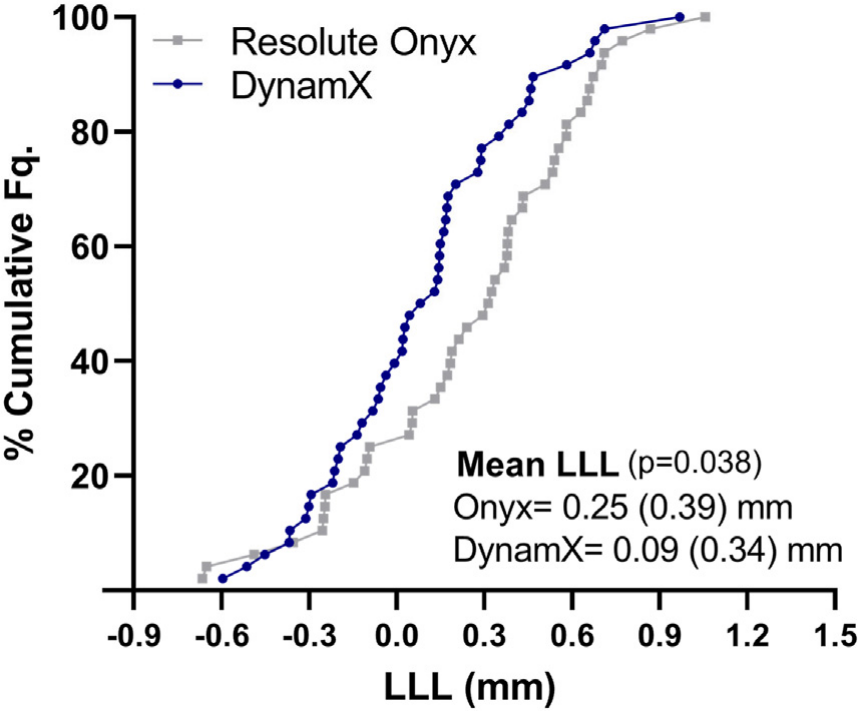
**Late lumen loss at12 months.** Fq = frequency, LLL = late lumen loss.

In the imaging cohort, the pre-procedure reference vessel diameter and percent diameter stenosis was 2.64 mm (SD 0.48) and 64.39% (SD 11.04) for DynamX group and 2.72 mm (SD 0.55) and 64.40% (SD 13.29) for the Resolute Onyx group. Pre-dilation was performed in all lesions in both groups and post-dilatation was performed in 80.0% (40/50) for DynamX and 78.0% (39/50) for Resolute Onyx (80.1% and 69.1% in the overall cohort, respectively). Acute gain was not different between DynamX and Resolute Onyx DES. However, at 12 months, paired data showed superior in-device LLL for DynamX compared to the Resolute Onyx DES: 0.09 mm (SD 0.34) [95% CI: −0.01; 0.19] versus 0.25 mm (SD 0.39) [95% CI: 0.13; 0.36], p = 0.038 (Table 3 and Fig. 3).

**Table 3:** Quantitative coronary angiography, intravascular ultrasound and optical coherence tomography (paired core laboratory analysis).

	DynamX	Resolute Onyx	p-value
**Angiographic data**	**(N = 48)**	**(N = 48)**	
In-device acute gain (mm)	1.66 (0.45)	1.75 (0.52)	0.41
	1.71 (1.28; 1.93)	1.66 (1.34; 2.24)	
In-segment acute gain (mm)	1.16 (0.43)	1.21 (0.57)	0.58
	1.10 (0.84; 1.44)	1.19 (0.81; 1.71)	
In-device recoil (mm)	0.21 (0.24)	0.27 (0.21)	0.33
	0.23 (0.04; 0.39)	0.33 (0.05; 0.44)	
In-device LLL at 12 months (mm)	0.09 (0.34)	0.25 (0.39)	0.038
	0.10 (−0.15; 0.29)	0.32 (0.01; 0.54)	
In-segment LLL at 12 months (mm)	−0.02 (0.38)	0.05 (0.34)	0.32
	−0.03 (−0.27; 0.19)	0.04 (−0.19; 0.27)	
In-device MLD post procedure (mm)	2.62 (0.49)	2.69 (0.44)	0.46
	2.58 (2.22; 2.91)	2.73 (2.28; 3.06)	
In-device MLD at 12 months (mm)	2.53 (0.57)	2.44 (0.59)	0.46
	2.42 (2.12; 2.99)	2.38 (2.06; 2.73)	
In-segment MLD post procedure (mm)	2.11 (0.50)	2.16 (0.51)	0.62
	2.06 (1.74; 2.48)	2.07 (1.81; 2.38)	
In-segment MLD at 12 months (mm)	2.13 (0.57)	2.11 (0.49)	0.84
	2.08 (1.69; 2.56)	2.00 (1.78; 2.41)	
In-device diameter stenosis post procedure (%)	9.56 (3.77)	10.24 (5.22)	0.77^a^
	9.23 (7.28; 11.48)	9.17 (6.34; 12.83)	
In-device diameter stenosis at 12 months (%)	12.70 (5.42)	17.33 (9.95)	0.051^a^
	1.02 (9.11; 16.56)	14.98 (9.53; 21.92)	
In-segment diameter stenosis post procedure, (%)	25.00 (11.37)	26.10 (10.48)	0.62
	22.94 (15.67; 33.35)	25.82 (17.46; 33.76)	
In-segment diameter stenosis at 12 months (%)	26.32 (11.07)	26.91 (10.39)	0.61^a^
	25.00 (17.90; 32.16)	26.27 (18.37; 33.49)	
**IVUS data**	**(N = 48)**	**(N = 47)**	
Mean lumen area post procedure (mm^2^)	7.54 (2.33)	8.11 (2.67)	0.26
	7.02 (5.83; 9.28)	7.58 (6.11; 9.92)	
Mean lumen area at 12-month (mm^2^)	7.19 (2.31)	7.55 (2.56)	0.47
	6.72 (5.35; 9.23)	7.08 (5.91; 8.87)	
Mean lumen area change from post procedure to 12-month (mm^2^)	−0.35 (0.79)	−0.57 (0.61)	0.14
	−0.32 (−0.86; 0.02)	−0.48 (−0.84; −0.10)	
In-device % neointimal obstruction at 12-month (%)	3.54 (2.28)	5.28 (3.32)	<0.001^a^
	3.01 (1.95; 3.98)	4.32 (3.34; 5.94)	
NIH volume (mm³)	5.75 (4.66)	9.67 (7.04)	<0.001^a^
	4.35 (2.63; 7.52)	7.70 (4.95; 11.20)	
**OCT**	**(N = 10)**	**(N = 9)**	
Mean NIH area (mm^2^)	0.71 (0.24)	1.28 (0.49)	0.009^a^
	0.77 (0.55; 0.91)	1.06 (0.96; 1.73)	
NIH volume (mm³)	15.43 (4.35)	24.13 (8.57)	0.018^a^
	15.80 (13.09; 16.74)	25.99 (17.66; 28.78)	
Derived NIH thickness (mm)	0.19 (0.05)	0.23 (0.06)	0.10
	0.19 (0.16; 0.22)	0.21 (0.18; 0.27)	
Freq. of covered struts per lesion (%)	98.47 (1.07)	97.39 (2.28)	0.46^a^
	98.51 (97.60; 99.14)	96.84 (95.36; 100)	
Freq. of malapposed struts per lesion (%)	0	0	–

Data are displayed as mean (SD) or median (interquartile range).

IVUS = intravascular ultrasound, LLL = late lumen loss, MLD = minimum lumen diameter, NIH = neointimal hyperplasia, OCT = optical coherence tomography.

a Assessed by Wilcoxon-Mann-Whitney U-test.

**Fig. 4:**
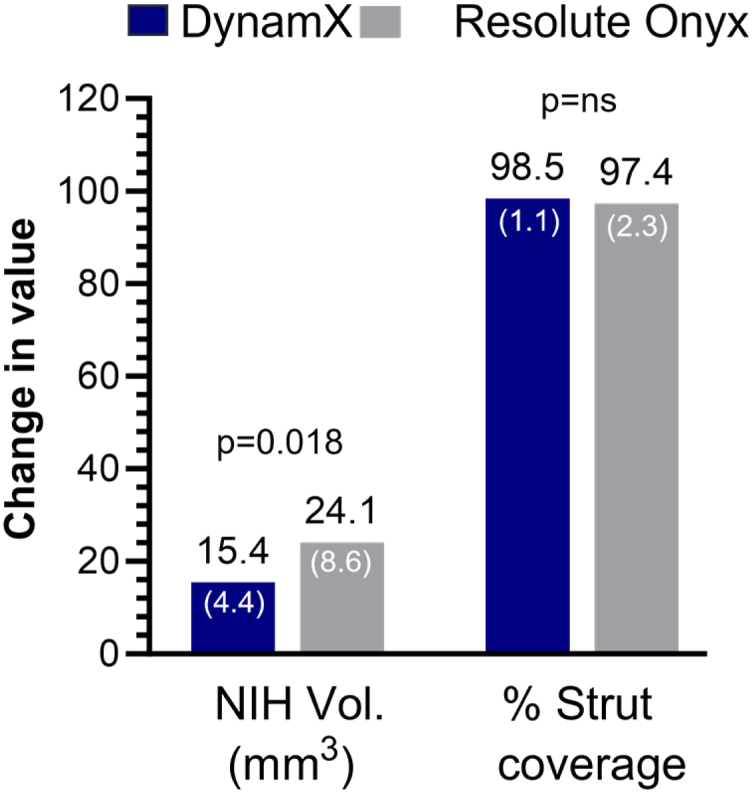
**Neointimal hyperplasia and strut coverage by optical coherence tomographyat 12 months.** Data are displayed as mean (SD). NIH = neointimal hyperplasia, ns = not significant, Vol = volume.

IVUS results showed that neointimal hyperplasia (NIH) volume was less in the DynamX group (5.75 mm³ [SD 4.66] versus 9.67 mm³ [SD 7.04], p < 0.001). Likewise, in the pull-back OCT imaging cohort NIH area and NIH volume were significantly lower in the DynamX group compared to the DES group at follow-up. Despite the lower NIH area of 0.71 (SD 0.24) versus 1.28 (SD 0.49) mm^2^, p = 0.009, OCT imaging showed there was no difference in % strut coverage, with nearly all struts covered in both groups (98.47% [SD 1.07] for DynamX versus 97.39% [SD 2.28] for Resolute Onyx, p = 0.46) and no observed malapposition (>200 μm) in either group (Table 3 and Fig. 4).

**Fig. 5:**
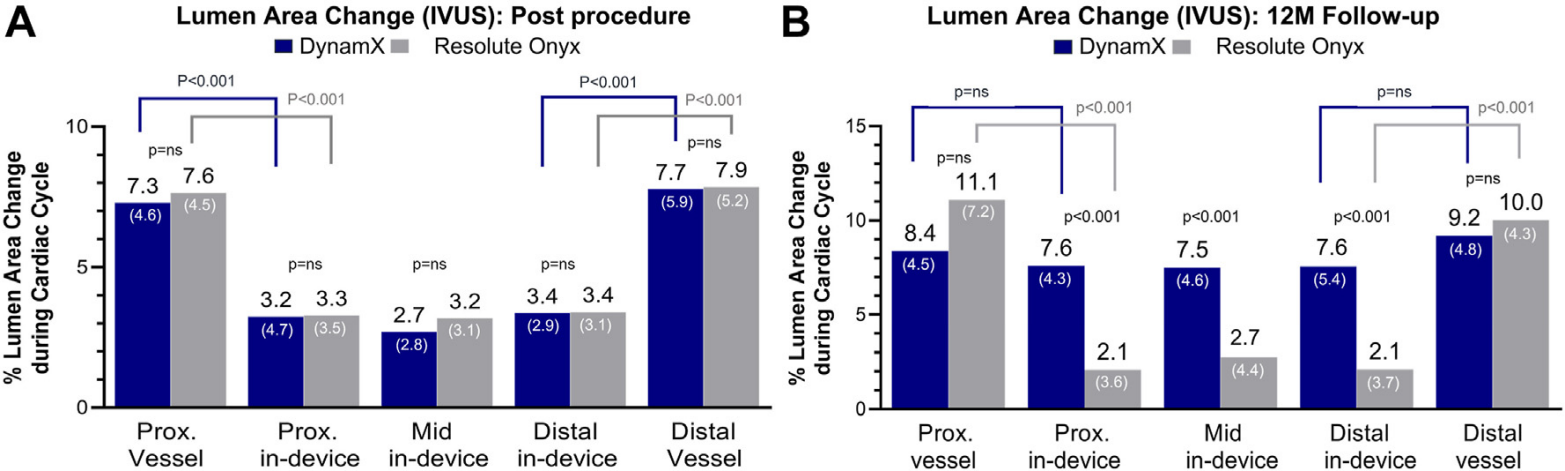
**Cyclic pulsatility assessment by intravascular ultrasound.** The figures show no difference in % lumen area change measured by IVUS in the proximal vessel, all in-device segments and distal vessel during a cardiac cycle in DynamX versus Resolute Onyx groups post procedure, whereas at 12 month follow up, DynamX shows a significantly higher % change in all in-device segments (p < 0.001) during a cardiac cycle as compared to the Resolute Onyx indicating restoration of pulsatility in treated segments with DynamX (N = 48 paired data for the DynamX group; N = 47 paired data for the Resolute Onyx group). Immediately post-procedure (A), there was a statistically significant difference between the pulsatility of the proximal and distal vessel segments and the in-device segment in both groups (all p < 0.001), whereas at 12 months (B), the difference was not significant anymore with DynamX, but it remained a statistically significant compliance mismatch for Resolute Onyx. Data are displayed as mean (SD). Ns = not significant, prox. = proximal.

Stationary IVUS imaging was performed to demonstrate pulsatility of the treated vessel segment during systole and diastole. While there was no difference amongst the groups immediately post-procedure, at follow-up, the DynamX sirolimus-eluting bioadaptor group had superior in-device lumen area and device area changes during the cardiac cycle compared to the DES group (mid in-device lumen area change of 7.5% [95% CI: 6.1; 8.9] versus 2.7% [95% CI: 1.4; 4.0], p < 0.001). There was a significant difference between in-device lumen area and the proximal and distal vessel segments in both groups post procedure. While the difference remained significant for the DES group, the in-device lumen area change for the bioadaptor at 12 months was similar to the proximal and distal vessel segment (Fig. 5), representing restoration of device/vessel compliance in the DynamX group. Likewise, blood flow changes derived by lumen area changes measured by IVUS using the Hagen Poiseuille flow equation show that the percentage blood flow increase per heartbeat improved from 6.5% [SD 5.6] at baseline to 16.7% [SD 8.9] at 12 months (p < 0.001) for the bioadaptor, while there was no difference between post-procedure and follow-up for the DES-group (Table 4).

**Table 4:** Blood flow changes during cardiac cycle calculated from stationary intravascular ultrasound (exploratory analysis, paired data).

	%Blood flow increase between systole and diastole		p-value
	Post-procedure	12-month FU	
DynamX	6.5 (5.6)	16.7 (8.9)	<0.001
Resolute Onyx	6.7 (4.2)	5.2 (8.0)	0.25
p-value	0.88	<0.001	/

Data are displayed as mean (SD).

FU = follow-up.

**Fig. 6:**
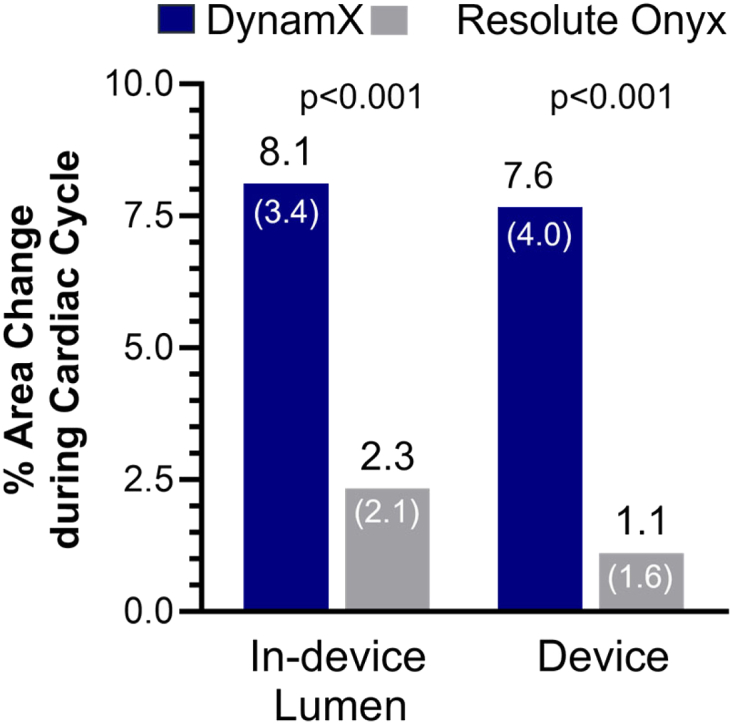
**Cyclic pulsatility assessment by optical coherence tomography.** The graphs represent the in-device lumen area change and the device-area change at 12 months; both were statistically significant different amongst the groups (p < 0.001). Paired data of 9 patients in the DynamX and 9 patients in the Resolute Onyx group. Data are displayed as mean (SD).

The cyclic pulsatility observed by stationary IVUS (N = 48 paired data for the DynamX group; N = 47 paired data for the Resolute Onyx group) was confirmed by stationary OCT with 8.1% (SD 3.5) [95% CI: 5.5; 10.8] versus 2.3% (SD 2.2) [95% CI: 0.6; 4.0], p < 0.001 for change in in-device lumen area and 7.6% (SD 4.0) [95% CI: 4.5; 10.6] versus 1.1% (SD 1.4) [95% CI: 0.0; 2.2], p < 0.001 for change in in-device device area in the DynamX versus the Resolute Onyx group (Fig. 6, for representative OCT images, see Supplemental Fig. 2).

### Post-hoc analyses

Post-hoc subgroup analyses on TLF amongst the subgroups of gender, diabetes, age ≥65 years, number of lesions, number of study devices used, and regions (Japan and Europe/New Zealand) are provided in Supplemental Table 5.

Furthermore, in the subsets of LAD and long lesions (≥23 mm) DynamX had a significantly better LLL and % diameter stenosis compared to the Resolute Onyx DES, and in small vessels (≤2.75 mm), DynamX had a significantly lower % diameter stenosis (Supplemental Table 6 and Supplemental Fig. 3).

The % change in in-device plaque volume was 3% [SD 19] versus 12% [SD 24], p = 0.03 by IVUS (Supplemental Table 7 and Supplemental Fig. 4), whereas in the proximal and distal untreated segments the change in plaque volume per segment length was similar for both groups. In treated lesions not containing calcium and in lipid-containing lesions, the plaque volume regressed in the DynamX group (−4% [SD 18] versus 9% [SD 22], p = 0.03 and −9% [SD 18] versus 10% [SD 18], p = 0.008) respectively (Supplemental Fig. 4). In this imaging cohort, 88% (44/50) in the DynamX group versus 90% (45/50) in the Resolute Onyx group were on lipid-lowering medication at baseline and 92% versus 94% at 12-month follow-up (92% versus 91% with lipid-containing lesions).

## Discussion

The BIOADAPTOR-RCT is the first trial comparing the novel bioadaptor technology with a contemporary DES. Outcomes at 12 months showed non-inferior safety, similar acute performance and significantly better effectiveness in reducing LLL, % diameter stenosis, restoration of vessel cyclic pulsatility, and restoration of vessel compliance. To the best of our knowledge, this is the first RCT that reports significantly better effectiveness restoration of vessel cyclic pulsatility, vessel compliance, and vessel function compared to a state-of-the art DES in the *de novo* coronary lesions.

In terms of safety, 12-month data are most relevant as they reflect the time period of resorption of the drug-polymer coating, unlocking of the bioadaptor and uncaging the vessel. TLF was low in both groups and observed in 1.8% of patients in the DynamX group versus 2.8% in the Resolute Onyx group, p_non-inferiority_ < 0.001. Considering that there is no difference in clinical outcomes among contemporary DES despite improvements in stent designs such as ultrathin struts^13^^,^^23^ it can be surmised that DynamX meets the safety and efficacy profile of latest generation DES.

The absence of cardiovascular death, the low TV-MI rate, and all TV-MIs being peri-procedural (except for one event in a protocol violator where the DynamX was implanted in the same lesion that was previously treated with a BRS, which also resulted in a device thrombosis and TLR on day 3), are all encouraging and ease potential concerns of triggering events through the uncaging of the bioadaptor. The second TLR in the bioadaptor arm also occurred in a lesion that violated the exclusion criteria (severely calcified lesion).

The low TV-MI and device thrombosis rates, as well as the low rate of restenosis could be attributed to vessel healing, regulation of vascular homeostasis and the restoration of vessel pulsatility, compliance, and function, the contractile phenotype switch of smooth muscle cell in the neointima at nine months, that is higher than for a conventional DES,^12^ and the improved torsional, axial, and rotational motion and reduced stress after unlocking of the bioadaptor,^9^ likely providing vaso-protective properties to the treated vessel.

Our results are consistent with the previous mechanistic study of 50 patients treated with the DynamX bioadaptor, in which no definite or probable stent thrombosis and no TLF-event between 9 and 24 months were reported.^11^

In terms of procedural outcomes, the device, lesion, and procedure success rates and the acute post-procedural lumen gain confirm that the bioadaptor's implantability and acute performance is similar to DES. This is an advantage over BRS which require more complicated implantation techniques and have shown inferior procedural outcomes, such as acute gain and post-procedural diameter stenosis compared to DES in RCTs.^24^^,^^25^

The DynamX bioadaptor is formed from three thin biocompatible metal sinusoid strands that unlock, so that the device is no longer a fixed, rigid structure, as demonstrated by restoring the vessel dynamic motion and function, but still provides the necessary support of the diseased vessel wall. Improvement of vasomotion and cyclic pulsatility of the “unlocked” compared to the “locked” version has been previously proven in the single-arm DynamX Mechanistic trial.^10^^,^^11^

Likewise, DynamX established cyclic pulsatility at 12 months by stationary IVUS and OCT in the BIOADAPTOR-RCT. For DynamX, the change in intra-device mean lumen area during the cardiac cycle was similar to the proximal and distal reference segments and close to the 8–10% seen in native coronary arteries.^21^^,^^22^ This provides evidence of the unlocking of the bioadaptor and freeing of the vessel, whereas the Resolute Onyx DES could only marginally comply to the cardiac cycle (mid in-device lumen area change of 7.5% for the bioadaptor versus 2.7% for the DES, p < 0.001, by 12-month IVUS). Importantly, this led to compliance restoration between the treated and proximal and distal segments with the bioadaptor compared to continued compliance mismatch for DES, which is known to lead to flow disturbances, flow and wall shear stress alterations, increased edge restenosis, and an increased risk of stent thrombosis.^9^^,^^26^, ^27^, ^28^

Restoration of vessel function and the ability for positive adaptive remodeling could be the reason for the statistically lower LLL, lower diameter stenosis and lower NIH area and volume in the DynamX compared to the Resolute Onyx group, yet not at the cost of strut coverage which was near complete (98.47%) with a NIH thickness of 0.19 mm (SD 0.05) mm at 12 months. Moreover, the resolution of the compliance mismatch between the in-device and proximal and distal segments in the unlocked bioadaptor might have also contributed to the absence of any TLF event and any definite device thrombosis beyond 6 months.

Importantly, the low LLL, diameter stenosis and NIH parameters at follow-up show that after uncaging, the bioadaptor still has sufficient radial strength, and that there is no tissue excess in response to the uncaging and its increased vessel motion.

The potential of the bioadaptor technology to adapt to vessel motion might be particularly useful in subgroups such as the LAD (prone to rotation during the cardiac cycle), small vessels, long lesions, and high plaque burden lesions such as lipid containing lesions.^29^^,^^30^ Likewise, the LLL and post-procedural diameter stenosis were significantly superior in the DynamX group compared to the Resolute Onyx group in the subset of LAD, small vessels, and long lesions.

Additionally, the plaque volume remained stable in the overall cohort of DynamX treated patients whereas it increased in Resolute Onyx treated segment (change in plaque volume from baseline to 12 months of 3% [SD 19] versus 12% [SD 24], p = 0.03). When excluding calcium containing plaque lesions, the in-device plaque volume regressed in the DynamX-treated group and increased in the Resolute Onyx group, while the change in plaque volume in the proximal and distal native segment was not different between the groups. These data suggest that the lower plaque volume changes in the DynamX group compared to the DES group might be attributable to the restoration of motion and function of the vessel working in synergy with the systemic lipid-lowering drugs, though lipid levels were not captured during the trial. These data are hypothesis generating and would need to be confirmed in larger patient series with controlled lipid levels.

This study has several limitations. Due to the visual differences between the devices, the operators could not be blinded to the randomisation assignment with the associated potential for bias. However, the imaging core laboratory and the clinical events committee were blinded to the treatment. The observed 12-month TLF-rate was lower than the expected 12-month TLF-rate of the sample size calculation. The number of screening failures was not assessed. Furthermore, data were obtained in a selected patient population that may be a subset of daily practice. For instance, patients with acute myocardial infarction and complex coronary artery lesions were excluded. The strength is the study rigor, with high follow-up compliance, and a sample size that is based on a primary clinical endpoint.

In conclusion, these are the first randomised data comparing the novel bioadaptor technology with contemporary DES and, to the best of our knowledge, the first RCT data that show superiority of a PCI-device compared to contemporary DES. The data presented herein provide promise that the DynamX sirolimus-eluting bioadaptor improved effectiveness over DES may pay off long-term by reducing long-term adverse events. It demonstrates not only non-inferiority of 12-month TLF against the current gold standard (DES), but also improved effectiveness parameters such as LLL and vessel function.

## Contributors

All authors read and approved the final version of the manuscript. SS: per ICMJE terms: conception and design of the work, acquisition, analysis, or interpretation of data for the work, drafting the work and revising it critically, final approval of the version to be published, agreement to be accountable for all aspects of the work in ensuring that questions related to the accuracy or integrity of any part of the work are appropriately investigated and resolved. Per CRediT terms: conceptualization, investigation, methodology, project administration, resources, supervision, writing- original draft, writing-review and editing. SS and SV directly accessed and verified the underlying data of this manuscript. AN, ZM, PCS, M-CM: analysis and interpretation of data, critically revising the work, final approval of the version to be published, agreement to be accountable for all aspects of the work. Per CRediT terms: investigation, resource, supervision, writing-review&editing, validation, project administration, visualization. JB, HMN, MW, AtN, AT, TK, SY, YS, DS, MV, MM, HM, NW: acquisition and interpretation of data, critically revising the work, final approval of the version to be published, agreement to be accountable for all aspects of the work. Per CRediT terms: investigation, resource, supervision, writing-review&editing.

## Data sharing statement

Data are available from the corresponding author upon reasonable request, but with an embargo of 12 months.

## Declaration of interests

Shigeru Saito reports consulting fees from Elixir Medical which are paid to the NPO International TRI network, Johan Bennett reports consulting fees from Biotronik AG, Boston Scientific, Abbott Vascular and Elixir, Holger Nef reports honoraria, payments for expert testimony, and support for attending meetings from Elixir Medical, Helge Möllmann reports grants/contracts and support for attending meetings from Abbott, Boston Scientific and Medtronic and payment/honoraria from Abbott, Boston Scientific, Edwards Lifesciences and Medtronic, Nikos Werner reports speakers honorarium and research grants from Abiomed, Boston Scientific, Edwards Lifesciences, Medtronic, and Shockwave, and is an advisory board member for ElixirMedical, Antoinette Neylon is a shareholder of CERC, Zlatko Mehmedbegovic is an independent core laboratory specialist at CERC, Pieter C. Smits reports institutional research grants from Abbott Vascular, SMT, Microport and Daiichy Sankyo, consulting fees of Abbott Vascular, Astra Zeneca, Terumo and Microport, and payments/honoraria from Abiomed, Terumo and Microport, participates in the DSMBs of the Protector, Legacy and ASET Japan trials, in the global advisory board of Abbott, and the European advisory board of Terumo (the latter paid to its institution), and is a minor shareholder of CERC. Marie-Claude Morice is a minor shareholder of Electroducer and Basecamps, and a shareholder and CEO of CERC, Stefan Verheye reports consulting fees and payment/honoraria from Elixir and Neovasc. The other authors report no conflict of interest.

## Appendix A Supplementary data

Appendix 1

Appendix 2 Clinical Investigation Plan Japan V 1.4.

Supplemental Movie 1 Postprocedural stationary intravascular imaging showing the acute radial strength of the connected (caged) bioadaptor. Movie provided by Elixir Medical.

Supplemental Movie 2 Stationary intravascular imaging at 12 months showing regained vessel motion after the unlocking of the bioadaptor and subsequent uncaging of the vessel. Movie provided by Elixir Medical.
