# Supplementary material for: First randomised controlled trial comparing the sirolimus-eluting bioadaptor with the zotarolimus-eluting drug-eluting stent in patients with de novo coronary artery lesions: 12-month clinical and imaging data from the multi-centre, international, BIODAPTOR-RCT
Source: eClinicalMedicine. 2023 Oct 24;65:102304. doi: 10.1016/j.eclinm.2023.102304 (PMC10725075; doi:10.1016/j.eclinm.2023.102304)
Supplement: Appendix 1 [file mmc1.docx]

**Supplemental Material**

**Supplemental Table 1: Procedural details**

| **Procedure details** | **DynamX**  **(N=223 patients)**  **(N=226 lesions)** | **Resolute Onyx**  **(N=222 patients)**  **(N=230 lesions)** |  |
| --- | --- | --- | --- |
|  |  |  | **P-value** |
|  |  |  |  |
| Procedure time (min) | 25·6 (20·0) | 24·0 (21·0) | 0·42 |
| Total contrast used (ml) | 142·6 (57·5) | 143·4 (56·0) | 0·88 |
| Total radiation dose (mGy) | 1006·8 (773·9) | 1062·3 (912·3) | 0·54 |
| Number of subjects with 1 target lesion | 220 (98·7%) | 214 (96·4%) | 0·13 |
| Number of subjects with 2 target lesions | 3 (1·3%) | 8 (3·6%) | 0·13 |
| Number of subjects with non-target lesions | 17 (7·6%) | 18 (8·1%) | 0·85 |
| Number of diseased vessels |  |  | 0·42 |
| 1 | 201 (90·1%) | 194 (87·4%) |  |
| 2 | 20 (9·0%) | 23 (10·4%) |  |
| >2 | 2 (0·9%) | 5 (2·3%) |  |
| Pre-dilatation | 226 (100·0%) | 230 (100·0%) | NA |
| % residual stenosis after pre-dilatation | 22.4 (14·5) | 22.2 (11·3) | 0·22 |
| Post-dilatation | 181 (80·1%) | 159 (69·1%) | 0·007 |
| Successful post-dilatation | 181 (100·0%) | 159 (100·0%) | NA |
| % residual stenosis after post-dilatation | 0·5 (1·9) | 0·3 (1·4) | 0·22 |
| Number of stents used (implanted or not) |  |  | 0·83 |
| 1 | 216 (95·6%) | 217 (94·3%) |  |
| 2 | 10 (4·4%) | 12 (5·2%) |  |
| 3 | 0 (0·0%) | 1 (0·4%) |  |
| Inflation pressure (atm) | 12·6 (2·1) | 13·4 (2·5) | 0·0002 |
| Inflation duration (s) | 31·8 (13·5) | 28·7 (14·3) | 0·018 |
| Bailout procedure* | 1 (0·4%) | 9 (4·1%) | 0·011 |
|  | N=236 devices | N=244 devices |  |
| Device diameter (mm) | 3·1 (0·4) | 3·0 (0·4) | 0·43 |
| Device length (mm) | 21·7 (6·0) | 21·9 (6·4) | 0·67 |

Data are displayed as mean ± SD or n (%). *requirement to implant a second device due to complications such as dissection, occlusive complication with blood flow decrease, malapposition, or device damage. NA=not applicable

**Supplemental Figure 1: Target lesion failure by Kaplan-Meier estimates**


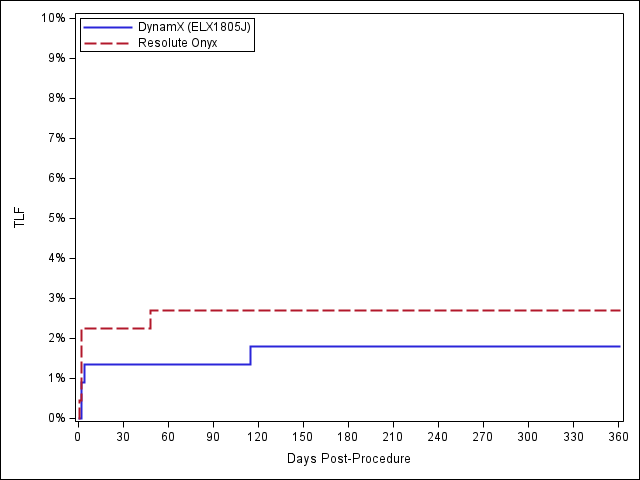


TLF-target lesion failure

| **Number at Risk** | **Day 0** | **Day 180** | **Day 360** |
| --- | --- | --- | --- |
| DynamX | 223 | 219 | 217 |
| Resolute Onyx | 222 | 214 | 214 |

P_non-inferiority_  < 0.001

Difference [95% CI, -3.34%; -1.38%]

**Supplemental Table 2: Antiplatelet and lipid lowering drugs at six and 12 months**

| **Medication** | **DynamX  (N=223)** | **Resolute Onyx (N=222)** |
| --- | --- | --- |
| At 6 months |  |  |
| ASA  P2Y12 inhibitor  Lipid lowering drugs | 207 (92·8%)  190 (85·2%)  202 (90·6%) | 209 (94·1%)  202 (91·0%)  201 (90·5%) |
| At 12 months |  |  |
| ASA  P2Y12 inhibitor  Lipid lowering drugs | 182 (81·6%)  144 (64·6%)  201 (90·1%) | 183 (82·4%)  151 (68·0%)  193 (86·9%) |

Data are displayed as n (%). ASA=acetylsalicylic acid

**Supplemental Table 3: Details of patients with target lesion failures**

|  | **DynamX** | **Resolute Onyx** |
| --- | --- | --- |
| Target lesion failure | 4 | 7 |
| Cardiovascular death | 0 | 2  1 Sudden death at POD 1  1 unwitnessed death POD 30 |
| Target-vessel MI | 3  2 PPMI+1 spontaneous on POD 3  Thereof the spontaneous MI was caused by a ST and led to a TLR* | 4  All PPMI, 1 patient also had TLR |
| Clinically-driven-TLR | 2* | 2 |

Data are displayed as n. *The inclusion of the patient that experienced a spontaneous MI, ST and TLR violated the exclusion criteria of in-stent restenosis (the bioadaptor was implanted in a lesion previously treated with a bioresorbable scaffold). The second TLR occurred at three and eight months in a patient that violated the exclusion criterion of severely calcified lesions (the lesion required 4 balloons for successful predilatation). MI=myocardial infarction, PPMI=periprocedural myocardial infarction, POD=postoperative day, ST=stent thrombosis, TLR=target lesion revascularisation.

**Supplemental Table 4: Secondary clinical endpoints**

| **Endpoint/Components** | **DynamX** | **Resolute Onyx** |  |  |
| --- | --- | --- | --- | --- |
|  | **(N=223)** | **(N=222)** | **Difference (%)** | **95% CI of Difference** |
| All-cause mortality | 1/222 (0·5%) | 3/216 (1·4%) | -0·9% | -3·7% to 1·3% |
|  | 0·0% to 2·5% | 0·3% to 4·0% |  |  |
| Cardiovascular death | 0/221 (0·0%) | 2/215 (0·9%) | -0·9% | -3·4% to 0·8% |
|  | 0·0% to 1·7% | 0·1% to 3·3% |  |  |
| Non-cardiovascular death | 1/222 (0·5%) | 1/214 (0·5%) | -0·0% | -2·2% to 2·1% |
|  | 0·0% to 2·5% | 0·0% to 2·6% |  |  |
| Stroke | 0/221 (0·0%) | 0/213 (0·0%) | 0·0% | N/A |
|  | 0·0% to 1·7% | 0·0% to 1·7% |  |  |
| Myocardial infarction | 3/221 (1·4%) | 4/213 (1·9%) | -0·5% | -3·6% to 2·3% |
|  | 0·3% to 3·9% | 0·5% to 4·7% |  |  |
| Target-vessel MI | 3/221 (1·4%) | 4/213 (1·9%) | -0·5% | -3·6% to 2·3% |
|  | 0·3% to 3·9% | 0·5% to 4·7% |  |  |
| Non-target vessel MI | 0/221 (0·0%) | 0/213 (0·0%) | 0·0% | N/A |
|  | 0·0% to 1·7% | 0·0% to 1·7% |  |  |
| Q-wave MI | 0/221 (0·0%) | 2/213 (0·9%) | -0·9% | -3·4% to 0·8% |
|  | 0·0% to 1·7% | 0·1% to 3·4% |  |  |
| Non-Q-wave MI | 2/221 (0·9%) | 2/213 (0·9%) | -0·0% | -2·6% to 2·4% |
|  | 0·1% to 3·2% | 0·1% to 3·4% |  |  |
| Not evaluable if Q-wave MI | 1/221 (0·5%) | 0/213 (0·0%) | 0·5% | -1·4% to 2·5% |
|  | 0·0% to 2·5% | 0·0% to 1·7% |  |  |
| All Revascularisation | 13/221 (5·9%) | 11/213 (5·2%) | 0·7% | -3·9% to 5·3% |
|  | 3·2% to 9·8% | 2·6% to 9·1% |  |  |
| TVR | 3/221 (1·4%) | 4/213 (1·9%) | -0·5% | -3·6% to 2·3% |
|  | 0·3% to 3·9% | 0·5% to 4·7% |  |  |
| Clinically-driven TVR | 2/221 (0·9%) | 3/213 (1·4%) | -0·5% | -3·3% to 2·0% |
|  | 0·1% to 3·2% | 0·3% to 4·1% |  |  |
| Non-TVR | 12/221 (5·4%) | 8/213 (3·8%) | 1·7% | -2·5% to 6·1% |
|  | 2·8% to 9·3% | 1·6% to 7·3% |  |  |
| TLR | 3/221 (1·4%) | 2/213 (0·9%) | 0·4% | -2·2% to 3·1% |
|  | 0·3% to 3.9% | 0·1% to 3·4% |  |  |
| Clinically-driven TLR | 2/221 (0·9%) | 1/213 (0·5%) | 0·4% | -1·8% to 2·9% |
|  | 0·1% to 3·2% | 0·0% to 2·6% |  |  |
| Probable or definite stent thrombosis | 1/221 (0·5%) | 1/214 (0·5%) | -0·0% | -2·2% to 2·2% |
|  | 0·0% to 2·5% | 0·0% to 2·6% |  |  |
| Patient oriented clinical endpoint | 16/222 (7·2%) | 17/216 (7·9%) | -0·7% | -5·9% to 4·5% |
|  | 4·2% to 11·4% | 4·7% to 12·3% |  |  |
| Composite of all-cause mortality, MI and revascularization | 16/222 (7·2%) | 17/216 (7·9%) | -0·7% | -5·9% to 4·5% |
|  | 4·2% to 11·4% | 4·7% to 12·3% |  |  |
| Composite of cardiovascular death, TV-MI and clinically-driven TVR | 4/221 (1·8%) | 8/215 (3·7%) | -1·9% | -5·6% to 1·4% |
|  | 0·5% to 4·6% | 1·6% to 7·2% |  |  |
| Composite of cardiovascular death, stroke, MI and revascularization | 15/221 (6·8%) | 16/215 (7·4%) | -0·7% | -5·8% to 4·4% |
|  | 3·8% to 10·.9% | 4·3% to 11·8% |  |  |
| Composite of cardiovascular death, MI and revascularization | 15/221 (6·8%) | 16/215 (7·4%) | -0·7% | -5·8% to 4·.4% |
|  | 3·8% to 10·9% | 4·3% to 11·8% |  |  |
| Composite of cardiovascular death or TV-MI | 3/221 (1·4%) | 6/215 (2·8%) | -1·4% | -4·8% to 1·5% |
|  | 0·3% to 3·9% | 1·0% to 6·0% |  |  |
| Composite of all-cause death or MI | 4/222 (1·8%) | 7/216 (3·2%) | -1·4% | -5·0% to 1·8% |
|  | 0·5% to 4·5% | 1·3% to 6·6% |  |  |
| Composite of all-cause death, MI or TVR | 6/222 (2·7%) | 10/216 (4·6%) | -1·9% | -6·0% to 1·8% |
|  | 1·0% to 5·8% | 2·2% to 8·3% |  |  |

Data are displayed as n/N (%) and 95%CI. MI=myocardial infarction, TLR=target lesion revascularisation, TV=target vessel, TVR=target vessel revascularisation

**Supplemental Figure 2: Optical coherence tomographic lumen and device area tracings at 12-month follow-up**


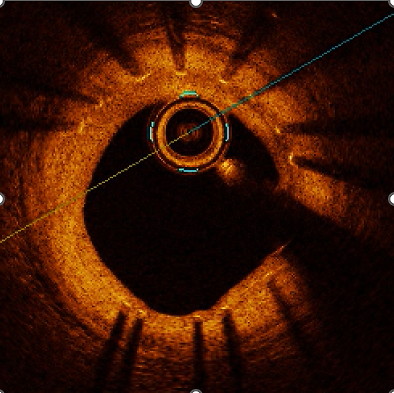


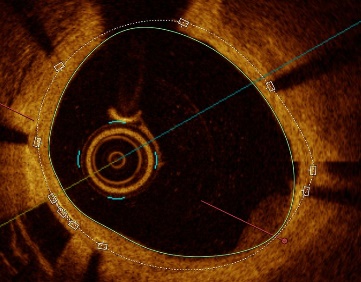

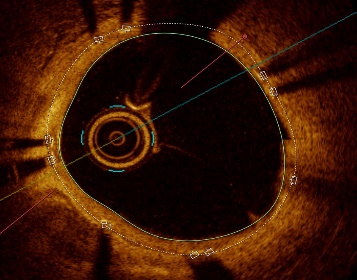

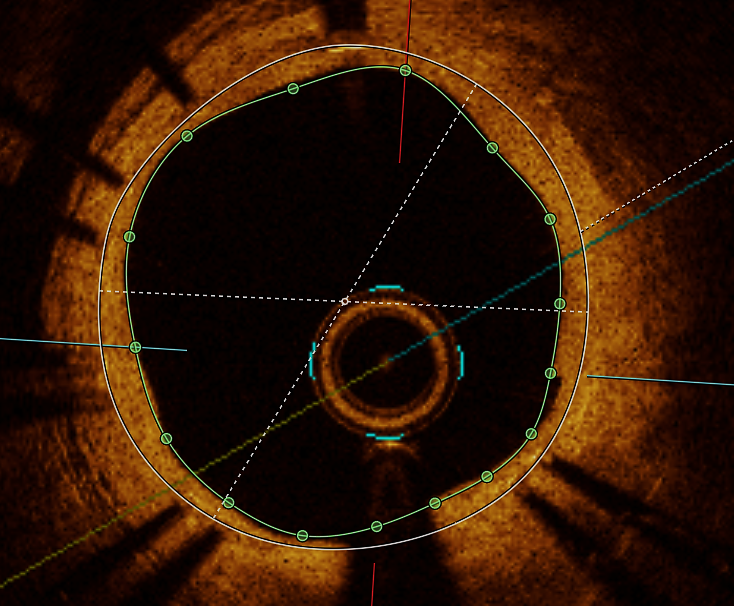

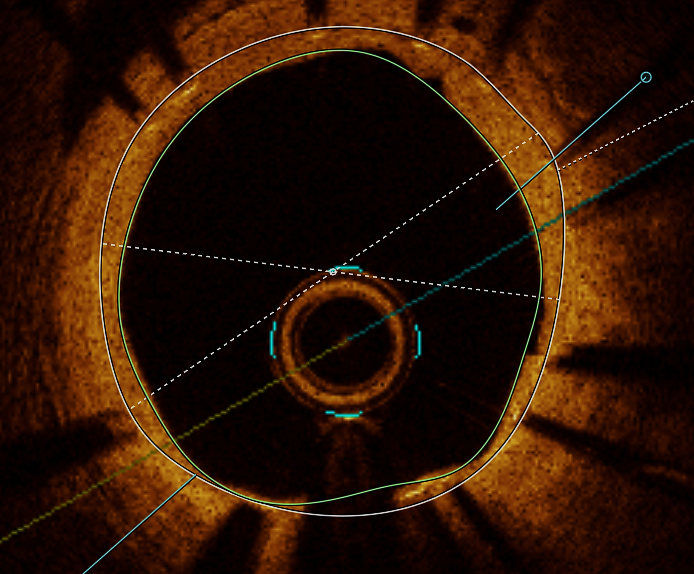


**Lumen 5.6 mm^2^**

**Device 7.0 mm^2^**

**Lumen 5.7 mm^2^**

**Device 7.1 mm^2^**


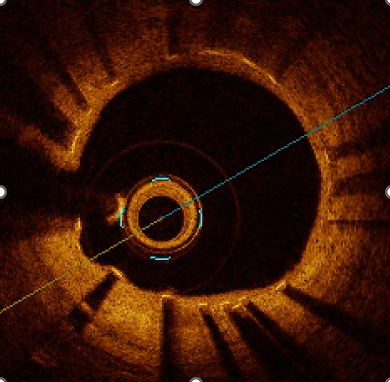


**Resolute Onyx**

**12M FUP**

**DynamX 12M FUP**

**A**

**B**

**C**

**Lumen 5.6 mm^2^**

**Device 6.4 mm^2^**

**Lumen 6.5 mm^2^**

**Device 7.9 mm^2^**

**Systole**

**Diastole**

**Systole**

**Diastole**

**Resolute Onyx**

**Resolute Onyx**

**DynamX**

**DynamX**

(A) Representative OCT images cross-sections at 12-month follow-up showing the Resolute Onyx and the DynamX bioadaptor with uncaging elements within the white circle. (B and C) Representative paired tracings of lumen and device area measurements during systole and diastole at 12 months post- Resolute Onyx (B) and DynamX (C) implantation. The unlocking of DynamX allows expansion of the device and uncaging of the vessel permitting the change in vessel lumen area at the 12-month follow-up that is evident during a cardiac cycle in DynamX implanted vessel compared to the Resolute Onyx implanted vessel.

**Supplemental Table 5: 12-month target lesion failure by subgroup-analysis**

| **Primary Endpoint** | **DynamX** | **Resolute Onyx** | **Hazard Ratio (95% CI)** | **Subgroup by Treatment Interaction** |
| --- | --- | --- | --- | --- |
|  | **(N=223)** | **(N=222)** |  |  |
|  |  |  |  |  |
| **All Patients** | 4 (1·8%) | 6 (2·7%) | 0·66 [0·19, 2·33] |  |
| Gender |  |  |  | 0·74 |
| Female | 1/49 (2·0%) | 1/49 (2·0%) | 1·00 [0·06, 15·99] |  |
| Male | 3/174 (1·7%) | 5/173 (2·9%) | 0·59 [0·14, 2·47] |  |
| Status of diabetes |  |  |  | 0·99 |
| Diabetes | 0/59 (0·0%) | 1/75 (1·3%) | < 0·01 [0·00, NA] |  |
| Non-diabetes | 4/164 (2·4%) | 5/147 (3·4%) | 0·71 [0·19, 2·64] |  |
| Age |  |  |  | 1·00 |
| ≥ 65 years old | 2/127 (1·6%) | 3/126 (2·4%) | 0·65 [0·11, 3·92] |  |
| < 65 years old | 2/96 (2·1%) | 3/96 (3·1%) | 0·66 [0·11, 3·95] |  |
| Number of lesions |  |  |  | 0·99 |
| Single | 3/218 (1·4%) | 6/214 (2·8%) | 0·49 [0·12, 1·95] |  |
| Multiple | 1/5 (20·0%)) | 0/8 (0·0%)) | > 100 [0·00, NA] |  |
| Number of vessels |  |  |  | 1·00 |
| Single | 4/201 (2·0%) | 6/214 (3·1%) | 0·64 [0·18, 2·26] |  |
| Multiple | 0/22 (0·0%) | 0/28 (0·0%) | < 0·01 [NA, NA] |  |
| Number of study device used (excluding bailout) |  |  |  | 0·99 |
| 1 | 3//213 (1·4%) | 6/207 (2·9%) | 0·49 [0·12, 1·97] |  |
| 2 | 1/12 (8·3%) | 0/13 (0·0%) | > 100 [0·00, NA] |  |
| Region |  |  |  | 1·00 |
| Japan | 0/110 (0·0%) | 0/113 (0·0%) | < 0·01 [NA, NA] |  |
| Europe and New Zealand | 4/113 (3·5%) | 6/109 (5·5%) | 0·63 [0·18, 2·24] |  |

Data are displayed as n/N (%) and Hazard ratio [95%CI]

**Supplemental Table 6: Quantitative coronary angiography at 12 months by exploratory subgroup analysis (core laboratory, paired data)**

| **In-Device Endpoints** | **DynamX** | **Resolute Onyx** | **P-value*** |
| --- | --- | --- | --- |
| **LAD subset** | **(N=28)** | **(N=23)** | / |
| LLL (mm) | -0·02 (0·31) | 0·24 (0·35) | 0·007 |
| Diameter stenosis (%) | 12·07 (4·73) | 18·97 (10·34) | 0·006 |
| **Small vessel (≤2.75 mm) subset** | **N=15** | **N=13** | / |
| LLL (mm) | 0·08 (0·27) | 0·26 (0·32) | 0·12 |
| Diameter stenosis (%) | 13·00 (5·46) | 18·26 (7·34) | 0·045 |
| **Long lesion (≥23 mm) subset** | **N=10** | **N=10** | / |
| LLL (mm) | -0·06 (0·42) | 0·38 (0·29) | 0·016 |
| Diameter stenosis (%) | 13·04 (3·10) | 22·93 (9·11) | 0·008 |

Data are displayed as mean (SD). *The analysis did not pass interaction testing. LAD=left anterior descending artery, LLL=late lumen loss

**Supplemental Figure 3: Late lumens loss (QCA)**


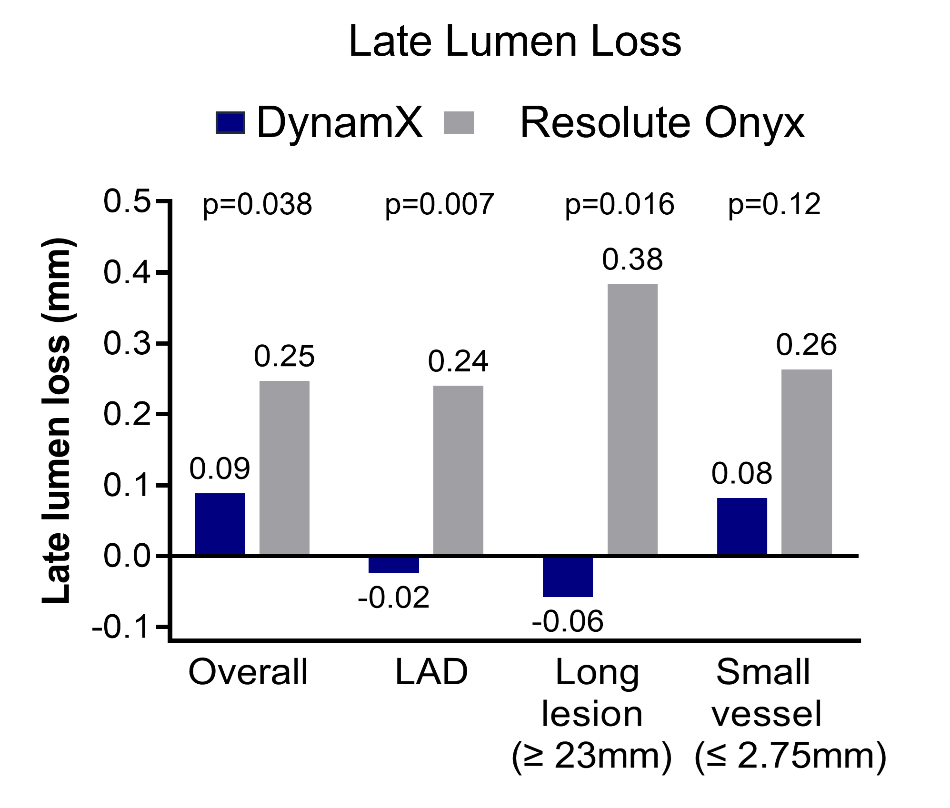


The late lumen loss was significantly lower for DynamX compared to Resolute Onyx for the overall population and the subgroups. LAD=left anterior descending, QCA=quantitative coronary angiography

**Supplemental Table 7: Change in plaque volume by intravascular ultrasound (exploratory analysis, paired data)**

|  | **Plaque Volume** | | **P-value** |
| --- | --- | --- | --- |
|  | **DynamX**  **(N=48)** | **Resolute Onyx**  **(N=47)** |  |
| **In-Device** |  |  |  |
| Plaque Volume at baseline, mm^3^ | 136·37 (58·14) | 148·57 (74·08) | 0.38 |
| Plaque Volume at 12M FU, mm^3^ | 140·26 (64·54) | 172·09 (100·58) | 0·16 |
| Absolute change in plaque volume from baseline to 12M FU, mm^3^ | 3·89 (23·20) | 18·22 (35·42) | 0·024 |
| **Artery segment Proximal to device** | **DynamX**  **(N=42)** | **Resolute Onyx**  **(N=42)** |  |
| Absolute change in plaque volume normalized per length from baseline to 12M FU | 0·67 (1·45) | 0·42 (1·73) | 0·99 |
| **Artery segment Distal to device** | **DynamX**  **(N=41)** | **Resolute Onyx**  **(N=42)** |  |
| Absolute change in plaque volume normalized per length from baseline to 12M FU | 0·50 (1·34) | 0·41 (1·30) | 0·50 |

Data are displayed as mean (SD). FU=follow-up

**Supplemental Figure 4: Plaque volume change by intravascular ultrasound**

1. **Non-calcified lesions**


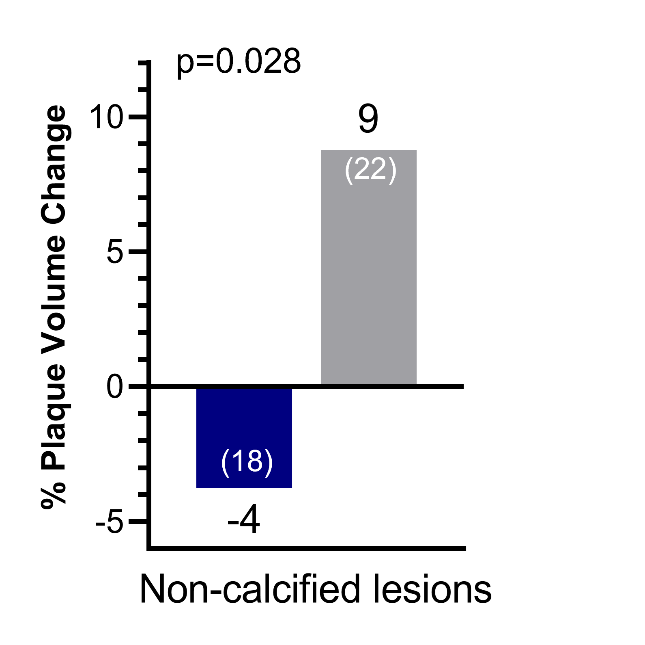

1. **Lipid containing lesions**


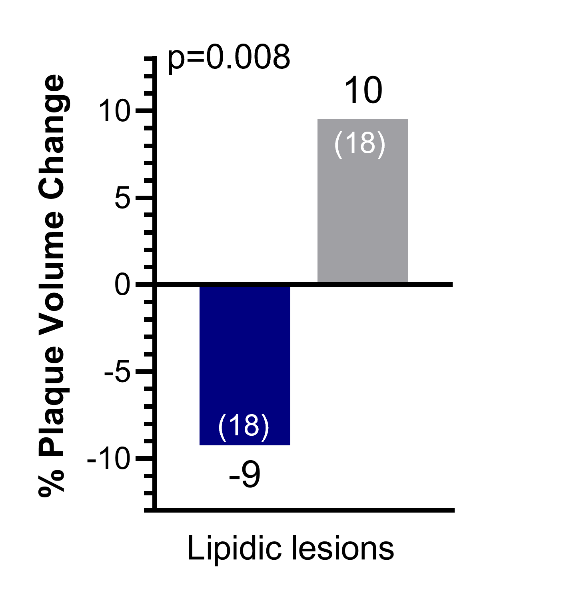


DynamX Resolute Onyx

(A) Plaque volume regressed in the bioadaptor group in patients without calcified lesions, defined as lesions with lipidic or fibrotic, but not calcified plaque (n=21 for DynamX and n=32 for Resolute Onyx), and (B) it regressed in patients with lipid containing lesions (n=12 for DynamX and n=22 for Resolute Onyx), defined as lesions with lipidic plaque, but not calcified plaque. Data are displayed as mean (SD).
