# Supplementary material for: First randomised controlled trial comparing the sirolimus-eluting bioadaptor with the zotarolimus-eluting drug-eluting stent in patients with de novo coronary artery lesions: 12-month clinical and imaging data from the multi-centre, international, BIODAPTOR-RCT
Source: eClinicalMedicine. 2023 Oct 24;65:102304. doi: 10.1016/j.eclinm.2023.102304 (PMC10725075; doi:10.1016/j.eclinm.2023.102304)
Supplement: Appendix 2 — Clinical Investigation Plan Japan V 1.4. [file mmc2.pdf]

Evaluation of a Sirolimus Eluting Bioadaptor as Compared to a Zotarolimus Eluting Stent in *De novo* Native Coronary Arteries

ELX-CL-1805
"BIOADAPTOR RCT STUDY"

Bioadaptor Study Protocol
Protocol number: ELX-CL-1805
Version 1.4

Sponsored By:
Elixir Medical

Confidentiality

This protocol should be handled as confidential information provided only to the relevant parties of this clinical study. For disclosure of any unpublished information contained in this document, a written approval should be obtained from the sponsor of this clinical study.

**Title**	Evaluation of a Sirolimus Eluting Bioadaptor as Compared to a Zotarolimus Eluting Stent in *De novo* Native Coronary Arteries<br>ELX-CL-1805 "BIOADAPTOR STUDY"
**Objective**	The objective of this study is to verify the safety and efficacy of the investigational device (ELX1805J) for the treatment of ischemic heart disease due to *de novo,* native coronary artery lesions
**Study design**	A prospective, multicenter, randomized (1:1; DynamX Bioadaptor (ELX1805J): Resolute Onyx), single-blind study, registering approximately 444 subjects at approximately 35 sites within Japan, Europe, and New Zealand to be conducted in two parallel cohorts. The combined Japan Bioadaptor RCT data and European Bioadaptor RCT data are intended to serve as the pivotal trial to support the regulatory approval in Japan with the Pharmaceuticals and Medical Devices Agency (PMDA).<br><br>**Bioadaptor RCT (Japan) Cohort**<br>Approximately 222 subjects (1:1 randomization) will be enrolled in Japan, to achieve a minimum of 202 evaluable subjects. All subjects will receive follow-up clinical assessments at 1, 6 and 12 months and every year for 5 years thereafter.<br><br>An Imaging Subset of approximately 100 Japan subjects will be included as part of the 222 randomized subjects in Japan. This includes 80 subjects with IVUS at Baseline & 12 Month Follow-Up and 20 subjects with IVUS & OCT at Baseline & 12 Month Follow-Up.<br><br>Single-group study (referred to as PK substudy)<br>The PK study will enroll 8 non-randomized subjects in Japan in subjects receiving only the ELX1805J. The PK substudy is being conducted to assess the blood pharmacokinetics of Sirolimus eluted from the ELX1805J Bioadaptor implanted in patients. PK measurement will be conducted at pre-treatment, 10 minutes, 30 minutes, 1, 2, 4, 6, 12, 24, 72 hours, and 7 days. In addition, all subjects will undergo clinical follow-up assessments at 1, 6 and 12 months and every year for 5 years thereafter. The PK substudy subjects are not considered part of the primary analysis population.<br><br>**Bioadaptor RCT (European) Cohort**<br>Approximately 222 subjects (1:1 randomization) will be enrolled in Europe, to achieve a minimum of 202 evaluable subjects. All subjects will receive follow-up clinical assessments at 1, 6 and 12 months and every year for 5 years thereafter.<br><br>Data from the Bioadaptor RCT European Cohort will be pooled with the data from the Bioadaptor RCT Japan Cohort to support primary and secondary endpoints. The primary endpoint analysis will be performed using the Intent-to-Treat (ITT) population on all subjects at the point of randomization.

Clinical Investigational Plan Internal Elixir Approval Page

Protocol Name: Evaluation of a Sirolimus Eluting Bioadaptor as Compared to a Zotarolimus Eluting Stent in *De novo* Native Coronary Arteries "BIOADAPTOR RCT"
Protocol No. ELX-CL-1805
Version: 1.4, 20-July-2022

Sponsor Representative:	Tina Cordaro, MPA<br>Sr. Director, Clinical Research, Elixir Medical Corporation
Signature and Date:	______________________________
Sponsor Representative:	Candace Elek, M.S.<br>Executive Vice President, Clinical Research, Elixir Medical Corporation
Signature and Date:	Digitally signed by Candace Elek<br>Date: 2022.10.06 10:42:42 -07'00'

Evaluation of a Sirolimus Eluting Bioadaptor as Compared to a Zotarolimus Eluting Stent in *De novo* Native Coronary Arteries

ELX-CL-1805
"BIOADAPTOR RCT STUDY"

Bioadaptor Study Protocol
Protocol number: ELX-CL-1805

Japan Protocol (English)
Version 1.4
20Jul2022

The Japan protocol is independently paginated

Evaluation of a Sirolimus Eluting Bioadaptor as Compared to a Zotarolimus Eluting Stent in *De novo* Native Coronary Arteries
ELX-CL-1805
"BIOADAPTOR RCT STUDY"

Bioadaptor Study Protocol

Protocol number: ELX-CL-1805

Confidential Information

Elixir Medical

Confidentiality

This protocol should be handled as confidential information provided only to the relevant parties of this clinical study. For disclosure of any unpublished information contained in this document, a written approval should be obtained from the sponsor of this clinical study.

Table of Contents

I. Definition of Terms 6
II. Time, Period and Other Rules 15
III. Summary of Protocol 16
1.0 Background and Rationale 25
1.1 Literature Review 25
1.2 Non-Clinical Studies 33
1.3 Risk Assessment 33
2. Study Objective 34
3. Clinical Study Plan 34
3.1 Type of Study 34
3.2 Study Design 34
3.3 Efficacy Endpoints and Safety Endpoints 34
3.4 Primary Endpoint 35
3.5 Secondary Endpoint 35
3.6 Number of Subjects and Rationale 37
3.7 Methods for Randomization and Blinding 38
4. Selection of Subjects 38
4.1 Consent of Subjects 38
4.2 Considerations for Informed Consent 40
4.3 Target Disease 40
4.4 Inclusion Criteria 40
4.5 Exclusion Criteria 42
5. Handling of Study Devices 44
5.1 Name of Study Devices 44
5.2 Shape and Structure of Study Devices 44
5.3 Packaging/Labeling of Study Devices 48
5.4 Control of Study Devices 49
5.5 Labeling of Shelf Life 50
6. Study Method 50
6.1 Method and Items of Observation 50
6.2 Flowchart of Clinical Study 56
7. Usage of Study Devices 58
7.1 Preparation of Study Procedures 58
7.2 Coronary Angiography and Confirmation of Location of Target Lesion 58
7.3 Treatment of Non-Target Lesion during Study Procedure 58

7.4 Treatment of Target Lesion 59
7.5 Bailout Procedure Immediately after Implantation..... 66
7.6 Completion of Study Procedure and Management after Study Procedure 66
8. Drugs, Therapies and Devices Used Concomitantly..... 67
8.1 Drugs, Therapies and Devices Used Concomitantly 67
8.2 Verification of Antiplatelet Therapy..... 68
9. Adverse Events..... 68
9.1 Basic Items 68
9.2 Definitions..... 68
9.3 Actions to be Taken for Adverse Events 69
9.4 Assessment of Adverse Events 70
9.5 Operational Check of Study Device during Study Procedure 71
10. Items to Ensure Safety of Clinical Study 71
10.1 Basic Items 71
10.2 Adverse Events Expected for Use of Investigational Device 72
10.3 Adverse Drug Reactions and Adverse Events Related to Study Devices 72
10.4 Adverse Drug Reactions Expected for Concomitant Drugs..... 73
10.5 Provision of New Information..... 73
10.6 Compensation for Health Damage 73
11. Evaluation of Efficacy 73
11.1 Multicenter randomized single-blind study 73
11.2 Single-group PK study..... 77
12. Evaluation of Safety 77
12.1 Safety Endpoints..... 77
12.2 Method for Evaluation of Safety..... 77
12.3 Method for Analysis of Endpoints for Safety 77
12.4 Record and Timing of Evaluation of Safety..... 78
13. Discontinuation/Suspension of Subject 78
13.1 Discontinuation Criteria for Subject and Rationale and Procedure for Discontinuation 78
14. Discontinuation/Suspension of Clinical Study..... 78
14.1 Partial Discontinuation/Suspension of Clinical Study..... 78
14.2 Discontinuation/Suspension of Entire Clinical Study..... 79
15. Statistical Analysis..... 79
15.1 Analysis population 79
15.2 Method of Statistical Analysis..... 79
15.3 Interim analysis 82
16. Deviation from, Change in and Revision of Protocol..... 82

16.1 Compliance with Protocol 82
16.2 Deviation from or Change in Protocol 83
16.3 Revisions of Protocol 83
17. Identification of Source Data 84
17.1 Source Documents 84
17.2 Items for which Records on eCRF are Considered to be Source Data 84
17.3 Data not Directly Recorded in eCRF and Considered to be Source Data 84
17.4 Collection of eCRF Data 84
18. Direct Access to Source Documents, etc. 85
18.1 Direct Access to Source Documents, etc. 85
18.2 Monitoring 85
19. Quality Control and Quality Assurance of Clinical Study 85
19.1 Items Related to Quality Control of Clinical Study 85
19.2 Items Related to Quality Assurance of Clinical Study 86
20. Retention of Records 86
20.1 Retention of Records 86
21. Items Related to Ethics and Compliance with GCP 87
21.1 Institutional Review Board 87
21.2 Protection of Privacy 87
22. Payment and Insurance 87
22.1 Payment 87
22.2 Compensation for Health Damage and Insurance 87
23. Rules for Publication of Results of Clinical Study 87
24. Organizational Control for Clinical Study 88
25. Study Period 88
26. Reference 89

[Annexes]
- Annex 1 Lists of Emergency Contacts
- Annex 2 Organizational Control for Clinical Study
- Annex 3 Organization for Clinical Study
- Annex 4 Participating Medical Institutions and Primary Investigators
- Annex 5 Packaging Form of Investigational Device
- Annex 6 Labeling of Investigational Device

[Attached Documents]
- Attached Document 1 Package Insert of Resolute Onyx -

I. Definition of Terms

(1) Abbreviation

Abbreviation	Unabbreviated description
ACC	American College of Cardiology
API	Active Pharmaceutical Ingredient
AHA	American Heart Association
AUC	Area under the blood concentration time curve
BMS	Bare Metal Stent
BVS	Bioresorbable vascular scaffold
CABG	Coronary Artery Bypass Graft
CAG	Coronary Angiography
CCS	Canadian Cardiovascular Society
CI	Clinically Indicated
CK	Creatine Kinase
CK-MB	Creatine Kinase – Myocardial Band isoenzyme
Cr	Creatinine
C_{max}	Maximum whole blood concentration
CTO	Chronic Total Occlusion
DEB	Drug Eluting Balloon
DES	Drug Eluting Stent
eCRF	Electronic Case Report Form
EDC	Electronic Data Capture
ELX1805J	Study device
FFR	Fractional Flow Reserve
FKBP-12	FK Binding Protein-12
GCP	Good Clinical Practice
Hb	Hemoglobin
Ht	Hematocrit
hs-cTn	High Sensitivity Cardiac Troponin
IB	Investigator’s Brochure
IFU	Instructions For Use
ISDN	Isosorbide Dinitrate
IVUS	Intravascular Ultrasound
ITT	Intent-To-Treat
LAD	Left Anterior Descending Artery
LCX	Left Circumflex Artery
LLL	Late Lumen Loss
LVEF	Left Ventricular Ejection Fraction
MACE	Major Adverse Cardiac Events
MI	Myocardial Infarction
MLD	Minimum Lumen Diameter
MSCT	Multi-Slice Computed Tomography
mTOR	mammalian Target Of Rapamycin
NYHA	New York Heart Association
OCT	Optical Coherence Tomography
PCI	Percutaneous Coronary Intervention

Abbreviation	Unabbreviated description
PLLA	Poly-L-Lactide
PLT	Platelet
POBA	Plain Old Balloon Angioplasty
PTE	Per-Treatment Evaluable
QCA	Quantitative Coronary Angiography
RBC	Red Blood Cell
RCA	Right Coronary Artery
RCT	Randomized Clinical Trial
RVD	Reference Vessel Diameter
SECBS	Sirolimus Eluting Coronary Bioadaptor System
STEMI	ST-Segment Elevation Myocardial Infarction
TIMI	Thrombolysis In Myocardial Infarction
TLF	Target Lesion Failure
TLR	Target Lesion Revascularization
TVF	Target Vessel Failure
TVR	Target Vessel Revascularization
$t_{1/2}$	Terminal elimination half-life
T_{max}	Time to reach the maximal whole blood concentration
WBC	White Blood Cell
%DS	% Diameter Stenosis

(2) Term

Term	Definition
ACC/AHA lesion classification	Type A (low risk, high acute success rate)<br>Length of stenosis < 10 mm<br>Concentric lesion<br>Easy-to-reach lesion<br>Mildly bent lesion segment (< 45°)<br>Regular lesion border<br>No (or mild) calcification<br>No complete occlusion<br>Non-ostial lesion<br>No major branch involvement<br>No thrombotic shadow<br>Type B* (moderate risk, moderate acute success rate)<br>*Lesion is classified as "B1" if it meets only one criterion and classified as "B2" if it meets two or more criteria.<br>10 to 20 mm in length of stenosis<br>Eccentric lesion<br>Moderately tortuous at proximal of lesion<br>Moderately bent lesion segment (≥ 45° and < 90°)<br>Irregular lesion border<br>Moderate or severe calcification<br>Complete occlusion with duration of occlusion of less than 3 months<br>Ostial lesion<br>Bifurcation lesions requiring double guidewires<br>Thrombotic shadow<br>Type C (high risk, low acute success rate)<br>Length of stenosis > 20 mm<br>Highly tortuous at proximal of lesion<br>Highly bent lesion segment (≥ 90°)<br>Complete occlusion with duration of occlusion of 3 months or longer<br>Inability to protect major side branch<br>Fragile lesion due to denatured venous graft
Braunwald classification	<Severity>

Term	Definition
(classification of unstable angina pectoris)	Class I: Newly developed severe angina pectoris or aggravated angina pectoris<br>• Angina pectoris developed within the past 2 months and accompanied by attacks frequently occurring at the rate of 3 times a day or more or aggravated angina pectoris accompanied by attacks occurring even with light exertion. No angina pectoris at rest.<br>Class II: Subacute angina pectoris at rest<br>• Angina pectoris at rest with attacks occurring at least once in the past one month and without any attack in the past 48 hours<br>Class III: Acute angina pectoris<br>• Angina pectoris at rest with attacks occurring at least once in the past 48 hours<br><Clinical status><br>Class A: Secondary unstable angina (due to non-cardiac factors such as anemia, fever, hypotension, tachycardia, etc.)<br>Class B: Primary unstable angina (no non-cardiac factors such as those listed in Class A)<br>Class C: Post-infarction unstable angina (unstable angina within two weeks of onset of MI)
CCS (Angina Classification)	None: No symptom of angina pectoris<br>Class I: Daily physical activities (walking, climbing stairs, etc.) do not cause anginal attack. Attacks occur with work, hard or sudden exercise during recreation or prolonged exertion.<br>Class II: Daily physical activities are slightly restricted. Anginal attacks occur while walking fast, climbing stairs quickly and climbing a slope and in situations such as after meal, in cold, in strong wind, under emotional stress or in few hours after rising. Angina if walking more than two blocks on the level and climbing more than one flight of ordinary stairs at a normal pace and in normal conditions.<br>Class III: Daily physical activities are significantly restricted. Attacks occur while walking 50 to 100 m on a flat ground or climbing upstairs at normal speed.<br>Class IV: Attacks occur with any physical activities and even at rest.
Thrombolysis In Myocardial Infarction Classification	TIMI 0: Complete occlusion with no antegrade blood flow and with periphery distal to lesion not visible on imaging.<br>TIMI 1: There is an obvious delay in imaging, and periphery is not visible on imaging.<br>TIMI 2: There is a delay in imaging, but periphery is visible on imaging.<br>TIMI 3: Normal imaging including periphery
de novo Lesion	Previously untreated lesion on native coronary artery
Ostial Lesion	Lesion within 5 mm of origin of coronary artery
Restenotic Lesion	Lesion within a part of blood vessel previously treated with PCI regardless of presence/absence of implanted stent
Target Lesion	Lesion to be treated with allocated study device
Target Vessel	Coronary artery with target lesion (left anterior descending branch, left circumflex, right coronary artery or a major side branches with 2mm or larger diameter)
Abrupt Closure	Acute occlusion<br>New occurrence of severe decrease in blood flow (TIMI0 to 1) in target vessel requiring relief using treatment not defined in protocol including

Term	Definition
	emergency surgery or leading to myocardial infarction and cardiovascular death. It is necessary to show that acute occlusion is related to mechanical dissection of target lesion or target vessel, coronary thrombosis, or severe spasm. No-reflow (due to limitation of blood flow in microvessels) which patency is retained with decreased blood flow in pericardial artery is not considered to be acute occlusion. In addition, occlusions indicating transient decrease in blood flow due to implantation of study device are not considered to be acute occlusion.<br>Subacute occlusion<br>Occlusions occurring within 30 days after study procedure<br>Imminent occlusion<br>Grade B dissection and stenosis of 50% or more in diameter stenosis or dissection of Grade C or higher
Distal Embolization	New, sudden blockage of blood flow at distal of lesion to be treated or filling defect
Device/ Stent thrombosis	For device /stent thrombosis used in this protocol, the ARC-2 definition is used.<br><br>Definite Stent/Scaffold Thrombosis<br>Angiographic confirmation of stent/scaffold thrombosis<br>• The presence of a thrombus that originates in the stent/scaffold or in the segment 5 mm proximal or distal to the stent/scaffold or in a side branch originating from the stented/scaffolded segment and the presence of at least 1 of the following criteria:<br>- Acute onset of ischemic symptoms at rest<br>- New electrocardiographic changes suggestive of acute ischemia<br>- Typical rise and fall in cardiac biomarkers (refer to definition of spontaneous myocardial infarction)<br>Or<br><br>Pathological confirmation of stent/scaffold thrombosis<br>Evidence of recent thrombus within the<br>- stent/scaffold determined at autopsy<br>- Examination of tissue retrieved following<br>- thrombectomy (visual/histology)<br><br>Probable stent/scaffold Thrombosis<br>Regardless of the time after the index procedure, any myocardial infarction that is related to documented acute ischemia in the territory of the implanted stent/scaffold without angiographic confirmation of stent/scaffold thrombosis and in the absence of any other obvious cause.<br><br>Silent stent/scaffold Occlusion<br>The incidental angiographic documentation of stent occlusion in the absence of clinical signs or symptoms is not considered stent thrombosis.
Bleeding Complication	Bleeding events associated with study procedure requiring transfusion or other intervention. Including hematoma and retroperitoneal hemorrhage requiring treatment.
Periprocedural Myocardial	Per ARC-2 Definition<br>Periprocedural Myocardial infarction

Term	Definition
Infarction (MI) Within 48 Hours (ARC-2)	Absolute rise in cardiac troponin (from baseline) ≥ 35 times upper reference limit, plus one (or more) of the following criteria:<br><br>1. New significant* Q waves or equivalent electrocardiographic signs of myocardial ischemia<br>2. Flow-limiting angiographic complications<br>3. New "substantial" loss of myocardium on imaging<br>** Q-wave criteria requires the development of new Q waves ≥40 ms in duration and ≥0.1 mV reduction in ≥2 contiguous leads.*
Periprocedural Myocardial Infarction Within 48 Hours (Modified ARC-2)<br><br>(In case of hs-cTn)	In case of high-sensitivity cardiac troponin:<br><br>Peri-procedural MI is defined as an absolute rise in cardiac troponin (from baseline) ≥ 70 times upper reference limit, plus one (or more) of the following criteria:<br>• New significant Q waves or equivalent electrocardiographic signs of myocardial ischemia<br>• Flow-limiting angiographic complications<br>• New "substantial" loss of myocardium on imaging<br>** Q-wave criteria require the development of new Q waves ≥ 40 ms in duration and ≥ 0.1 mV reduction in ≥2 contiguous leads.*
Significant Periprocedural Myocardial Injury (ARC-2)	Absolute rise in cardiac troponin (from baseline) ≥ 70 times upper reference limit (URL)
Periprocedural Myocardial Infarction (MI) in the absence of Troponins (SCAI)	In the absence of Troponins and only CK-MB, this protocol will follow SCAI definition for peri-procedural MI:<br><br>Absolute rise in CK-MB (from baseline) ≥10x upper limit of normal (ULN), or to ≥5 times ULN with new pathologic Q-waves in ≥2 contiguous leads or new persistent LBBB
Spontaneous MI (2012 3rd Universal Definition of MI)	Detection of a rise and/or fall of cardiac biomarker values [preferably cardiac troponin (cTn)] with at least one value above the 99th percentile URL and with at least one of the following:<br>• Symptoms of ischemia<br>• New or presumed new significant ST-segment-T wave (ST-T) changes or new LBBB<br>• Development of pathological Q waves in the ECG<br>• Imaging evidence of new loss of viable myocardium or new regional wall motion abnormality<br>• Identification of an intracoronary thrombus by angiography or autopsy
Death	Per ARC-2 Circulation 2018; 115: 2635-2650<br>Cardiovascular death:<br>Death resulting from cardiovascular causes. The following categories may be collected.<br>Death caused by or as a result of:

Term	Definition
	1. acute MI<br>2. sudden cardiac, including unwitnessed, death<br>3. heart failure<br>4. stroke<br>5. cardiovascular procedures<br>6. cardiovascular hemorrhage<br>7. other cardiovascular cause<br><br>Non-cardiovascular death<br>Death that is not thought to be the result of a cardiovascular cause. The following categories may be collected:<br>Death resulting from or caused by:<br>1. malignancy<br>2. pulmonary causes<br>3. infection (includes sepsis)<br>4. gastrointestinal causes<br>5. accident/trauma<br>6. other non-cardiovascular organ failure<br>7. other Non-cardiovascular cause<br><br>Undetermined cause of death<br>Death not attributable to any other category because of the absence of any relevant source documents. Such deaths will be classified as cardiovascular for end point determination.
Dissection	Classification of coronary artery dissection [NHLBI (National Heart, Lung, and Blood Institute) classification]<br>Grade A: Passage of contrast agent temporarily causes a small filling defect in vascular lumen.<br>Grade B: Contrast agent along blood vessel disappears with several heartbeats.<br>Grade C: Even after passage of contrast agent, extravascular area is still visible on contrast image.<br>Grade D: Manipulation of contrast agent in anterograde blood flow results in spiral filling defect.<br>Grade E: There is a filling defect due to obstruction delaying passage of contrast agent in peripheral vascular lumen.<br>Grade F: There is a filling defect due to complete occlusion.
Perforation	• Angiographic perforation: Perforation from study procedure confirmed at medical institution or the core laboratory<br>• Clinical perforation: Perforation requiring additional treatment (including closure of perforation and pericardial drainage) resulting in marked pericardial pooling, acute occlusion, myocardial infarction or death<br>• Pericardial hemorrhage/cardiac tamponade<br>• Perforation resulting in cardiac tamponade
No reflow	Decrease of antegrade blood flow is not related to occlusive lesion

Term	Definition
Stroke<br>Cerebrovascular accident (CVA)	Those with sudden onset of rotatory vertigo, numbness, dysphasia, weakness, visual field defect, stuttering, and other local neurological deficiency symptoms due to cerebrovascular accident such as cerebral hemorrhage, cerebral embolism, thrombosis, rupture of aneurysm lasting for more than 24 hours Detailed classifications are as follows.<br>• Ischemic stroke: Acute symptom of local insufficiency in brain, spine or retina caused by infarction in central nervous system tissue<br>• Hemorrhagic stroke: Acute symptom of local or systemic insufficiency in brain or spinal cord caused by nontraumatic hemorrhage in parenchyma, ventricle or subarachnoid<br>• Undetermined stroke: Strokes that are difficult to classify as ischemic or hemorrhagic stroke.<br><br>It is considered to be stroke when symptoms disappear within 24 hours and when the following treatments are conducted.<br>• Pharmacotherapy (thrombolytic agent, etc.)<br>• Non-pharmacotherapy [nerve intervention procedure (intracranial angioplasty, etc.)]
Emergent CABG	Emergency CABG performed for new cardiac ischemia caused by serious dissection associated with implantation of study device, pressure due to occlusion or stent treatment
Bailout	Additional treatment performed for symptoms and findings as follows:<br>・Vascular dissection which requires treatment<br>・Apparent occlusive complication with blood flow decrease in target vessel<br>・Chest pain, changes in ECG with ischemia that do not improve with balloon-dilation, medical therapy or thrombolytic agent<br>・Case which requires unscheduled additional stent to cover target lesion<br>・Case which causes possible clinical complication or confirms unsolvable device malapposition<br>・Any finding which proves device damage
TLF<br>Target Lesion Failure	Hierarchical composite endpoint comprised of:<br>• Cardiovascular death<br>• Myocardial infarction associated with target vessel (Q-wave and non Q-wave)<br>• CI-TLR requiring PCI or CABG
TLR<br>Target Lesion Revascularization	Re-PCI or CABG of the target lesion due to in-segment restenosis or other complications.

Term	Definition
CI-TLR<br>Clinically indicated Target Lesion Revascularization	Hierarchically<br>1. Core laboratory–reported fractional flow reserve ≤ 0.80 or instant wave-free ratio ≤ 0.89<br>2. Site-reported fractional flow reserve ≤ 0.80 or instant wave-free ratio ≤ 0.89<br>3. Quantitative coronary analysis (3 dimensional preferred) diameter stenosis > 50% (based on the average of multiple views) with either recurrent symptoms or positive noninvasive functional test<br>4. Quantitative coronary analysis (3 dimensional preferred) diameter stenosis > 70% (based on the average of multiple views) regardless of other criteria<br>5. Quantitative coronary analysis diameter stenosis > 70% (based on the worst view) regardless of other criteria
TVF<br>Target Vessel Failure	Hierarchical composite endpoint comprised of:<br>• Cardiovascular death<br>• Myocardial infarction associated with target vessel<br>• Target vessel revascularization
TVR<br>Target Vessel Revascularization	Re-PCI or CABG in the target vessel due to restenosis or other complications.
CI-TVR<br>Clinically indicated Target Vessel Revascularization	Revascularization performed for angina symptoms and signs of ischemia attributed to target vessel and % diameter stenosis of 50% or higher (based on QCA) or revascularization performed for % diameter stenosis of target vessel of 70% or higher (based on QCA) even without angina symptoms or signs of ischemia. Physician makes decision on TVR based on clinical findings before CAG. The core laboratory assesses the % diameter stenosis, and it may revise assessment by physician in case of disagreement.
MACE	Cardiovascular death, all myocardial infarction (Q-wave MI and non-Q-wave MI), CI-TLR (PCI or CABG)
In-Segment	Area between 5 mm at proximal end and 5 mm at distal end of implanted study device
In-Stent/Device	Within the boundaries of the implanted study device
Acute Gain	Change in MLD before and after implantation of study device. MLD is measured with QCA from the average of 2 orthogonal views.
MLD	Average of the narrowest part of lumen measured on the QCA images of target lesion, in-stent or in-segment taken in 2 perpendicular directions (if feasible). MLD is visually estimated by physician using CAG and measured by the core laboratory using QCA.
RVD	Average of the target vessel lumen diameters within 10 mm distal and 10 mm proximal of the lesion and is visually estimated during angiography by the Investigator; it is measured during QCA by the Angiographic Core Lab.
(%DS)<br>%Diameter Stenosis	This is calculated using the following equation and the QCA images taken in 2 perpendicular directions.<br>100 x (RVD – MLD)/RVD

Term	Definition
Acute Success<br><br>Device success, Lesion success, Procedure success	<u>Device Success</u><br>% diameter stenosis after implantation of allocated study device in target lesion is less than 30% by QCA (by visual estimation if QCA unavailable).<br><u>Lesion Success</u><br>% diameter stenosis after treatment of target lesion with PCI is less than 30% by QCA (by visual estimation if QCA unavailable).<br><u>Procedure Success</u><br>Lesion success is achieved for all target lesions, and there is no MACE during hospitalization.
Successful Pre-treatment	Mandatory pre-dilatation includes the use of 2 orthogonal views to confirm lesion inclusion and exclusion criteria. Successful pre-dilatation of a minimum of 1 Target Lesion ,defined as no waist in the inflated pre-dilatation (using two orthogonal views) with a pre-dilatation balloon diameter size approximately 0.25 mm smaller than reference vessel diameter but not more than 0.5 mm smaller than the reference vessel diameter. A residual diameter stenosis prior to study device implantation by visual estimate is recommended to be <30%.
Late Lumen Loss	It is calculated by subtracting MLD at follow-up from MLD at immediately after study procedure (after post-dilation of target lesion).
Angiographic Binary Restenosis Rate (ABR)	Percentage of subjects with % diameter stenosis of 50% or higher at time of follow-up using QCA

II. Time, Period and Other Rules

Time, period, etc. specified in this clinical study are defined as follows.

1. Time
(1) A day is defined as 24 hours, from 0:00 to 23:59. 24:00 means 0:00 of the next day.
(2) Initiation of study procedure in RCT trial is defined as time of Randomization. Initiation of study procedure in PK trial is defined as time of insertion of ELX1805.
(3) Completion of study procedure is defined as time of removal of guiding catheter from subject in both RCT trial and PK trial.

2. Definition of period
(1) Starting point of a period is defined as date of occurrence of event, and the day is referred to as Day 0 (start day).
(2) When study procedure spans over 2 days, date of initiation of study procedure is considered to be date of study procedure.
(3) Preoperative Day 1 refers to the day before study procedure, and postoperative Day 1 refers to the following day of study procedure.
(4) Postoperative Month 1 refers to postoperative Day 30, and postoperative Year 1 refers Day 365.

3. Enrollment of subjects
(1) One subject is counted as one case.
(2) If a subject has two target lesions, this is counted as one case, two lesions.

III. Summary of Protocol

<table>
<tr><td>Title</td><td>Randomized Clinical Study of the Sirolimus-Eluting Coronary Bioadaptor System (ELX1805J) Bioadaptor Study</td></tr>
<tr><td>Objective</td><td>The objective of this study is to verify the safety and efficacy of the investigational device (ELX1805J) for the treatment of ischemic heart disease due to de novo, native coronary artery lesions</td></tr>
<tr><td>Study design</td><td>Multicenter, randomized, single-blind study (referred to as Bioadaptor Study). 222 patients will be enrolled.

Up to 2 de novo lesions located in 2 separate native coronary arteries designated as target lesions may be treated. The target lesion(s) must measure between 2.25 mm and 4.0 mm in diameter and ≤ 34 mm in length, to be covered by a single ELX1805J Bioadaptor or a single control device. Patients should not be randomized until after satisfactory pre-dilatation of the target lesion has been performed.

A non-target lesion may be treated if located in separate epicardial vessel (RCA, LCX or LAD) using an approved 'olimus drug eluting stent provided the treatment of this non-target lesion is done prior to the treatment of the target lesion(s), and the treatment of the non-target lesion is considered successful. The segment should be located such that any injury that might occur during intervention can be clearly attributable to that treated segment.

The study is designed to randomly allocate 222 total subjects in a 1:1 ratio (test: control) to achieve a minimum of 202 evaluable subjects. All subjects will receive follow-up clinical assessments at 1, 6 and 12 months and every year for 5 years thereafter. Additionally, Imaging Follow-up with IVUS and IVUS + OCT will be collected in the Imaging subsets.

IVUS Imaging Subset (80pts)
The IVUS imaging subset of approximately 80 patients will undergo angiography and IVUS assessments at baseline and 12 month follow-up.

IVUS + OCT Imaging Subset (20pts)
The IVUS + OCT imaging subset of approximately 20 patients will undergo angiography, IVUS + OCT assessments at baseline and 12 month follow-up.

Single-group study (referred to as PK substudy)
Up to 2 de novo lesions located in 2 separate native coronary arteries designated as target lesions may be treated. The PK study will enroll 8 Japanese subjects and is being conducted to assess the blood pharmacokinetics of Sirolimus eluted from the ELX1805J Bioadaptor implanted in patients. PK measurement will be conducted at pre-treatment, 10 minutes, 30 minutes, 1, 2, 4, 6, 12, 24, 72 hours, and 7 days. In addition, all subjects will undergo clinical follow-up assessments at 1, 6 and 12 months and every year for 5 years thereafter.</td></tr>
<tr><td>Total number of subjects</td><td><table><tr><td>Investigational device (ELX1805J) group</td><td>111subjects</td><td>222 subjects</td></tr></table></td></tr>
</table>

Control device (Resolute Onyx) group	111 subjects	

PK study: 8 subjects (ELX1805J)

Study device

Bioadaptor Study
(1)Investigational device: ELX1805J

Bioadaptor Length (mm)		14	15	18	23	28	32	38
Bioadaptor diameter (mm)	2.25	X		X	X	X	X	X
	2.5	X	-	X	X	X	X	X
	2.75	X	-	X	X	X	X	X
	3.0	X	-	X	X	X	X	X
	3.5	X	-	X	X	X	X	X
	4.0	-	X	X	X	X	X	X

(2) Control device: Resolute Onyx Zotarolimus Eluting Coronary Stent System (Medtronic Japan Co., Ltd.)

Stent length (mm)		15	18	22	30	34	38
Stent diameter (mm)	2.25	X	X	X	X	X	X
	2.5	X	X	X	X	X	X
	2.75	X	X	X	X	X	X
	3.0	X	X	X	X	X	X
	3.5	X	X	X	X	X	X
	4.0	X	X	X	X	X	X

PK study: ELX1805J

Bioadaptor Length (mm)		14	15	18	23	28	32	38
Bioadaptor diameter (mm)	2.25	X		X	X	X	X	X
	2.5	X	-	X	X	X	X	X
	2.75	X	-	X	X	X	X	X
	3.0	X	-	X	X	X	X	X
	3.5	X	-	X	X	X	X	X
	4.0	-	X	X	X	X	X	X

Clinical Endpoints:

Primary Endpoint: Target lesion failure (TLF) assessed at 12 months. TLF is a composite endpoint defined as cardiovascular death, target-vessel MI, and clinically-indicated target lesion revascularization (CI-TLR)

Secondary Endpoints:

Efficacy endpoints

- Acute success rates:
  Lesion success rate, device success rate, procedure success rate

Clinical endpoints

Measured at 30 days, 6 months, 12 months, 2, 3, 4 and 5 years:

- TLF
- Patient Oriented Clinical Endpoint: Overall cardiovascular outcomes from the patient's perspective. This endpoint is a composite endpoint that includes all-cause mortality (cardiac and non-cardiac), stroke, MI (target vessel and non-target vessel) and revascularization (target vessel and non-target vessel)
- A composite of all-cause mortality, MI (target vessel or non-target vessel) and revascularization (target vessel or non-target vessel)

	• Composite of cardiovascular death, target vessel myocardial infarction (TV-MI)*, or clinically-indicated target vessel revascularization (CI-TVR)<br>• Composite of cardiovascular death, stroke, MI (target vessel or non-target vessel) and revascularization (target vessel or non-target vessel)<br>• Composite of cardiovascular death, MI (target vessel or non-target vessel) and revascularization (target vessel or non-target vessel)<br>• Clinically-indicated target lesion revascularization (CI-TLR)TLR<br>• Target vessel revascularization (TVR)<br>• Clinically-indicated TVR (CI-TVR)<br>• Revascularization(target vessel or non-target vessel)<br>• Q-wave MI<br>• Non Q-wave MI<br>• MI (target vessel or non-target vessel)<br>• Target vessel MI<br>• Cardiovascular death<br>• All-cause death<br>• Composite of cardiovascular death or target vessel MIComposite of all-cause death or MI(target vessel or non-target vessel)<br>• Composite of all-cause death, MI(target vessel or non-target vessel), or TVR<br>• Composite of probable or definite stent thrombosis‡<br>• Probable stent thrombosis‡<br>• Definite stent thrombosis‡<br>** Defined as myocardial infarction not clearly attributed to a non-target vessel*<br>*‡ Defined as per the Academic Research Consortium (ARC-2) criteria*
**Imaging Endpoints QCA , IVUS, and OCT**	**QCA endpoints:**<br>• Acute recoil<br>• Late lumen loss (in-stent and in-segment) at 12-month follow-up<br>• Change in vessel angulation from baseline, post-stent and 12-month follow-up<br>• MLD post-procedure and 12 months<br>• % DS post-procedure and 12 months<br><br>**IVUS endpoints:**<br>• Change in mean lumen area from post-procedure to 12-month follow-up<br>• In-stent % neointimal obstruction at 12-month follow-up<br>• In-stent late lumen loss at 12-month follow-up<br>• Acute, persistent and late stent malapposition<br><br>**OCT Endpoints:**<br>• % Strut coverage<br>• Neointimal thickness<br>• Vessel Pulsatility - % change in Lumen Area and Device Area during systole and diastole by stationary OCT<br>• Additional parameters may be assessed
**Treatment Strategy**	• Treatment of up to two *de novo* native coronary artery lesions located in separate epicardial territories able to be covered by a single device<br>• A non-target lesion located in a separate epicardial vessel may be treated with a non-study device. However, this non-target lesion should be treated

	first; and only after successful and optimal treatment may treatment of any target lesion be performed.<br>• The target lesion (s) is to be treated only after all clinical and angiographic inclusion criteria have been met.<br><br>**ELX1805J usage:**<br>• Mandatory pre-dilatation includes the use of 2 orthogonal views to confirm lesion inclusion and exclusion criteria. Successful pre-dilatation of a minimum of 1 Target Lesion, defined as no waist in the inflated pre-dilatation balloon (using two orthogonal views) with a pre-dilatation balloon diameter size approximately 0.25 mm smaller than reference vessel diameter but not more than 0.5 mm smaller than the reference vessel diameter. A residual diameter stenosis prior to study device implantation by visual estimate is recommended to be <30%.<br>• Patients with Grade A or B dissections after pre-dilatation that can be covered by a single study device may be treated per the implantation procedure described in Procedure for Implantation below.<br>• Lesions with pre-dilatation dissections Grade C or higher or Grade A or B dissections requiring treatment that cannot be covered by a single ELX 1805J device are excluded from the study<br>• Using either visual assessment or on-line QCA, select the appropriate size stent diameter and length. If the correct size is not available the patient should not be included in the study<br>• Select a stent diameter equal to or greater than the mean reference vessel diameter (RVD). **Mean RVD should not be greater than 4.0mm and Dmax should not be larger than 4.25mm**<br>• The stent length should cover the lesion and at least 2mm of healthy vessel on either side (healthy to healthy).<br>• Expand stent to at least nominal pressure and not greater than RBP<br>• Post-dilatation at operator's discretion as is the use of intra-vascular imaging techniques for patients not included in the IVUS subset. If post dilatation is performed, post-dilate with a non-compliant balloon and hold pressure for at least 30 seconds unless prevented by chest pain/ECG changes to ensure adequate stent apposition in all stent segments.<br>• Goal is 10% or less and no more than 15% residual diameter stenosis (DS)<br><br>**ZES usage:**<br>• Pre-dilatation should be performed according to the manufacturer IFU<br>• Patients with Grade A or B dissections after pre-dilatation that can be covered by a single study device may be treated per the implantation procedure described in Procedure for Implantation below.<br>• Lesions with pre-dilatation dissections Grade C or higher or Grade A or B dissections requiring treatment that cannot be covered by a single ZES device are excluded from the study

<table>
<tr>
<td></td>
<td>

Target lesion length should be ≤ 34 mm by visual estimate and have at least 2 mm of healthy vessel on either side of the lesion for coverage by a single ZES.
Expand ZES according to the manufacturer IFU
Post-dilate according to the manufacturer IFU
Goal is 10% or less and no more than 15% residual diameter stenosis (DS)

QCA/IVUS Sub-study

For those patients included in the IVUS sub-study, perform IVUS to assess stent expansion and apposition

Target in-stent cross sectional lumen area by IVUS should be no less than as recommended by the AVIO Trial (Chieffo, et al)[1]:

<table>
<tr><th>Stent/stent size</th><th>Target MLA</th></tr>
<tr><td>• 2.5 mm</td><td>• No less than 4.0mm²</td></tr>
<tr><td>• 3.0 mm</td><td>• No less than 6.0 mm²</td></tr>
<tr><td>• 3.5 mm</td><td>• No less than 8.0 mm²</td></tr>
<tr><td>• 4.0 mm</td><td>• No less than 10 mm²</td></tr>
</table>

Following IVUS (sub-study) or as appropriate, post-dilate again if needed with a non-compliant balloon and ensure that no clinically significant stent malapposition or uncovered dissection is present.

Bailout treatment

Bailout treatment may be conducted using up to one additional study device from the assigned treatment group sufficient to cover the affected area utilizing the shortest available study device. The devices must overlap by at least 1 - 2 mm. If additional stents or other sizes are needed, any non-study, approved DES stent incorporating an “olimus” drug and cobalt chromium metal or bare metal stent should be used.

Dual Antiplatelet Therapy (DAPT)

DAPT as per current ESC guidelines for PCI (Neumann et al, Eur Hear J 2018).

Stable coronary artery disease: For DAPT naïve patients, a loading dose for clopidogrel and aspirin 6-12 hour prior to the procedure but no less than 2 hours prior to the procedure.

ACS: loading dose of a potent $P2Y_{12}$ inhibitor (clopidogrel, ticlopidine, prasugrel, or ticagrelor) and aspirin.

DAPT therapy should be continued for a minimum of 6 months for stable patients and 12 months for ACS patients unless there are contraindications

</td>
</tr>
<tr>
<td>Key Inclusion Criteria</td>
<td>
Patients who meet all of the following criteria are eligible:

Patient must be ≥ 20 years of age.
Patient must have evidence of myocardial ischemia (e.g., stable or unstable angina, silent ischemia, positive functional study or electrocardiogram (ECG) changes consistent with ischemia)
Patients who are able to take dual anti-platelet therapy for 1 year following the index procedure and anticoagulants prior to/during the index procedure

</td>
</tr>
</table>

	• The subject is an acceptable candidate for Percutaneous Transluminal Coronary Angioplasty (PTCA), stenting, and emergent Coronary Artery Bypass Graft (CABG) surgery.<br>• The subject or subject's legally authorized representative has been informed of the nature of the study and agrees to its provisions and has provided written informed consent as approved by the Institutional Review Board or Ethics Committee of the respective clinical site.<br>• Women of childbearing potential with a negative pregnancy test within 7 days and women who are not pregnant or nursing<br>• Patient must agree to undergo all clinical study required follow up visits, angiograms, and imaging testing<br>• Patient must agree not to participate in any other clinical research study for a period of one year following the index procedure<br><br>**Angiographic inclusion criteria- Target Lesion/Vessel**<br><br>Confirmed in QCA assessment:<br>• Target lesion(s) must be de novo and located in a native coronary artery with a vessel mean diameter of ≥ 2.25 and ≤ 4.0 mm assessed<br><br>Confirmed by visual assessment:<br>• Target lesion(s) must be in a major artery or branch with a visually estimated stenosis of ≥ 50% and < 100% with a TIMI flow of >1. When two target lesions are treated, they must be located in separate major epicardial vessels<br>• The visually estimated target lesion length is ≤ 34 mm and must be able to be covered by a single 14/15/18/23/28/32/38 mm ELX1805J stent and have at least 2 mm of healthy vessel on either side, OR<br>• The visually estimated target lesion length is ≤ 34 mm and must be able to be covered by a single 15/18/22/30/34/38 mm ZES stent respectively and have at least 2 mm of healthy vessel on either side<br>• The lesion(s) must be successfully pre-dilated prior to enrollment<br>• Mandatory pre-dilatation includes the use of 2 orthogonal views to confirm lesion inclusion and exclusion criteria. Successful pre-dilatation of a minimum of 1 Target Lesion, defined as no waist in the inflated pre-dilatation balloon (using two orthogonal views) with a pre-dilatation balloon diameter size approximately 0.25 mm smaller than reference vessel diameter but not more than 0.5 mm smaller than the reference vessel diameter. A residual diameter stenosis prior to study device implantation by visual estimate is recommended to be <30%.<br>• Percutaneous intervention of lesions in a non-target vessel if:<br>o Not part of a another clinical investigation<br>o ≥ 30 days prior to the study index procedure<br>o ≥ 6 months after the study index procedure (planned)<br>• Percutaneous intervention of lesions located in the target vessel if:<br>o Not part of a clinical investigation<br>o ≥ 6 months prior to the study index procedure<br>o ≥ 12 months after the study index procedure (planned)<br>o Previous intervention was distal to and >10 mm from the target lesion<br><br>**Additional inclusion Criteria for PK study**<br>Patients participating in PK study may be treated with only ELX1805J

**Key Exclusion Criteria**	Patients must not have any of the following:<br>• The patient was diagnosed with an acute myocardial infarction within the past 72 hours and the CK and CKMB have not returned to normal (or cTn >15x ULN) and the patient is experiencing clinical symptoms indicative of ongoing ischemia<br>• Patient has a known hypersensitivity or contraindication to aspirin, both heparin and bivalirudin, clopidogrel, prasugrel or ticagrelor, cobalt, nickel, chromium, molybdenum, PLLA polymers or contrast sensitivity that cannot be adequately pre-medicated<br>• Patients with a history of allergic reaction or serious hypersensitivity to drugs exhibiting interactions with sirolimus, zotarolimus, everolimus, tacrolimus, temsirolimus, biolimus and other rapamycin, derivatives or analogues) or similar drugs<br>• Elective surgery is planned within the first 6 months after the procedure that will require discontinuing either aspirin or clopidogrel or other P2Y12 inhibitors<br>• Patient presenting with chronic (permanent) atrial or ventricular arrhythmia or current unstable ventricular arrhythmias<br>• Patient has a known left ventricular ejection fraction (LVEF) < 30%<br>• Patient has received a heart or other organ transplant or is on a waiting list for any organ transplant<br>• Patient has a malignancy that is not in remission.<br>• Patient is receiving immunosuppression therapy other than steroids and has known immunosuppressive or autoimmune disease (e.g. human immunodeficiency virus, systemic lupus erythematosus etc.)<br>• Patient is receiving chronic anticoagulation therapy (e.g., heparin, coumadin) that cannot be stopped and restarted according to local hospital standard procedures<br>• Patient has a platelet count < 100,000 cells/mm3 or > 700,000 cells/mm3, a WBC of < 3,000 cells/mm3, or documented or suspected to have cirrhosis of Child-Pugh ≥ Class B within 7 days before study procedure<br>• Patient has known renal insufficiency (e.g., serum creatinine level of more than 2.5 mg/dL within 7 days before study procedure, or patient on dialysis)<br>• Patient has a history of bleeding diathesis or coagulopathy or will refuse blood transfusions<br>• Patient has had a cerebrovascular accident (CVA) or transient ischemic neurological attack (TIA) within the past six months<br>• Patient has had a significant GI or urinary bleed within the past six months<br>• Patient has severe symptomatic heart failure (i.e., NYHA class IV)<br>• Patient has a medical condition that precludes safe 6 French sheath insertion<br>• Patient has other medical illness or known history of substance abuse (alcohol, cocaine, heroin etc.) that may cause non-compliance with the clinical study plan, confound the data interpretation or is associated with a limited life expectancy (i.e., less than one year)<br>• Patient is already participating in another clinical research study which has not reached the primary endpoint (long-term follow-up is not an exclusion)

	• Other patients whom primary investigator or subinvestigator determined to be ineligible for this clinical study<br><br>**Angiographic exclusion criteria**<br>• Patients with bypass graft to the target vessel or lesion is located in a bypass graft<br>• Patients with stent implanted within 10 mm of proximal or distal end of target lesion<br>• Patients with a target lesion involving a bifurcation of which the side branch will be jailed by the struts and:<br>• Side branch > 2.5 mm in diameter<br>• Side branch requires planned predilatation (including Kissing Balloon Technique), or Side branch has an ostial lesion or lesion with > 50% stenosis<br>• Patients suspected or confirmed with the QCA analysis of having stenotic lesion of more than 50% in target vessel in addition to target lesion<br>• Patients with target lesion in ostia located within 5 mm of origin of LAD, LCX or RCA<br>• Patients with stenotic lesion in left main trunk<br>• Patients with target lesion that is a chronic total occlusion (CTO) or ≤ TIMI 1 coronary flow in the target vessel<br>• Patients with target vessel that contains thrombus as indicated in pre-procedure angiographic, IVUS or OCT images<br>• Excessive tortuosity ≥ two 45° angles or extreme angulation (≥ 90°) proximal to or within the target lesion<br>• Patients with target vessel that has moderate to severe calcification that prevents complete angioplasty balloon (POBA with non-compliant balloon, or scoring balloon,) inflation or requires other devices such as rotational atherectomy, rotablator.<br>• Patients with dissection of Grade A or B that cannot be covered (including 2 mm distal to the dissection) with a single study device or with dissection of Grade C or higher<br>• Patients with 2 or more target lesions on 1 branch or target lesions on 3 branches that need to be treated during study procedure<br>• Target lesion involves a myocardial bridge<br><br>**Additional Exclusion Criteria for PK study**<br>• Patients with following criteria<br>o Patient with PCI within 180 days before study procedure<br>o Patient with plan to have staged PCI within 90 days after study procedure<br>o Patients who have non-target lesion
**Primary Analytical Population**	The primary endpoint analysis will be performed using the Intent-to-Treat (ITT) population.<br><br>Additional analysis will be performed using the Modified Intention-to-Treat (MITT) population defined as those patients who receive the assigned study device without major protocol violations

Observation Schedule

Observation period	Before study procedure	Pre-procedure to discharge		1 month	6 months	12 months	2, 3, 4 and 5 years
		Day 0		Day 30 (± 7 days)	Day 180 (± 30 days)	Day 365 (± 30 days)	(±30 days)
	Hospital visit	During study procedure	Completion of study procedure	TEL	Hospital visit /TEL	*Hospital visit	Hospital visit /TEL
Subject demographics	X						
Informed consent	X						
Enrollment		X					
12-lead ECG	X[2]		X[4]				
Left ventricular ejection fraction(LVEF)	X[1]						
Clinical laboratory test: WBC, Plt, and Creatinine	X[2]						
Clinical laboratory test: CK, CK-MB (or troponin)	X[3]		X[5]				
Clinical laboratory test: pregnancy test (women of childbearing potential)	X[2]						
Assessment of angina pectoris	X		X	X	X	X	X
Angiography (QCA)		X	X			X[6]	
IVUS/OCT		X[6]	X[6]			X[6]	
Concomitant medications	X[7]	X[8]	X[7]	X[7]	X[7]	X[7]	X[9]
Adverse events/malfunctions		X	X	X	X	X	X[10]

*For subjects not in the imaging subset, a virtual visit at the 12 month visit is acceptable if the patient is unable to return to the office due to COVID or other restrictions

1. Within 30 days before study procedure,
2. Within 7 days before study procedure,
3. Within 72 hours before study procedure (within 24 hours if there is any sign of AMI)
4. Conducted between 12 hours and 24 hours after study procedure, but if a patient is discharged within 12 hours after study procedure, conducted before its discharge.
5. Within 12 to 24 hours after study procedure (In cases of value higher than upper limit of institutional reference value, measurements are made 3 times at 8 hours interval or in compliance with its institutional method (physician's discretion)
6. Imaging subset patients. Complete clinical assessment before imaging.
7. Antiplatelet, anticoagulant, drugs related to heart disease
8. Drugs related to study procedure
9. Antiplatelet medications only
10. Following the 12 month visit, only the following AEs will be recorded: Bioadaptor/stent thrombosis rate, serious adverse events, malfunctions, all cardiovascular events regardless of seriousness or device relationship, all study device-related events and events for which the relationship to the study device is unknown, all unanticipated adverse device effects, and all CVAs (cerebrovascular accident)

Blood draws schedule in PK study (Conducted in selected site previously)

Observation period	Before study procedure	After study procedure														
		10 min		30 min		1 hr	2 hrs	4 hrs	6 hrs	12 hrs	24 hrs		72 hrs	7 Days		
Allowance		(±2 Min		(±4 Min)		(±8 Min)	(±15 Min)	(±30 Min)	(±30 Min)	(±30 Min)	(±2 Hrs)		(±6 Hrs)	(±24 Hrs)		
Blood test (PK study)	X	X		X		X	X	X	X	X	X		X	X		

1.0 Background and Rationale

1.1 Literature Review

The use of stenting for the treatment of atherosclerotic, de novo lesions in native coronary arteries has been shown to be a safe and effective treatment. However, the issue of restenosis, resulting either from angioplasty or stent placement, is believed by many to be one of the most significant problems in the field of interventional cardiology.[2,3,4]

The primary method used today to treat restenosis is the use of coronary stents coated with anti-proliferative drugs which allow localized delivery of the drug to the specific lesion site. Studies evaluating drug eluting stent systems loaded with anti-proliferative agents such as sirolimus, sirolimus analogs or paclitaxel, have shown success of these devices in reducing the amount of neointimal hyperplasia following stent implantation. Additionally, these drug eluting stents have been able to reduce the need of repeat revascularization procedures in patients.[5,6,7,8]

Next generation sirolimus-eluting stents with biodegradable polymer coatings have been recently approved in both Europe and Japan. Clinical data from multiple large-scale studies have demonstrated the safety and effectiveness of these devices including: Orsiro Sirolimus Eluting Coronary Stent System (Biotronik AG, Bülach, Switzerland), MiStent Sirolimus Eluting Absorbable Polymer Coronary Stent System (MiCell Technologies Inc., Durham, NC), and the BioMime Sirolimus Eluting Coronary Stent System (Meril Life Sciences Pvt. Ltd., Gujarat, India).[9,10,11,12,13,14]

Elixir Medical has two CE mark approved metallic DES: the DESyne® and the DESyne® BD Novolimus Eluting Coronary Stent Systems. Both were studied in randomized clinical trials and were shown to be non-inferior and superior to the control device (Medtronic Endeavor Zotarolimus Eluting Coronary Stent System (Medtronic, Santa Rosa, CA) for the primary endpoint of late lumen loss and both Elixir stents showed low 5-year clinical results.[15,16]

While the restenosis rates have been significantly reduced following the use of metal-based drug eluting stents (DES), the stent is a permanent implant and prevents the normal vessel motion (expansion and contraction) due to the metallic caging of the vessel. One solution for this was the introduction of fully bioresorbable stents or scaffolds. These devices are able to locally deliver drug to the lesion area, and due to the material, the scaffold was able to bioabsorb over time and promised to allow vascular restoration including lumen enlargement and vasomotion.

The Abbott Vascular BVS scaffold (ABSORB) was the first drug eluting fully bioabsorbable scaffold to be evaluated for feasibility and safety. The BVS scaffold is a

PLLA scaffold with a topcoat of a mixture of poly (D-lactide) (PDLLA) and Everolimus, an analog of sirolimus. Everolimus is approved for use on the Xience V Everolimus Eluting Scaffold as well as an immunosuppressant to prevent organ rejection following transplant and is marketed as Certican® (Novartis Pharmaceuticals Corporation). The scaffold was evaluated in numerous clinical studies as has received both CE Mark approval as well as FDA approval. There were however, questions about the long-term safety with an increase in device oriented cardiac events driven by an increase in target vessel MI and scaffold thrombosis as compared to the control Xience stent.[17] It is speculated that the long bioresorption time of this scaffold could be one main culprit.

In contrast to the ABSORB scaffold, Elixir Medical designed and studied a second generation scaffold. The DESolve® Novolimus Eluting Bioresorbable Scaffold System (NEBCSS) is comprised of a poly-L-lactic-acid (PLLA) based bioresorbable scaffold, coated with a matrix of the novel drug Novolimus (a sirolimus metabolite) and a polylactide-based polymer. Of primary significance is the shorter degradation/resorption profile of the polymer backbone, shown by in-vitro and in-vivo preclinical testing, to have approximately 90% degradation (reduction in molecular weight) in six months and near complete (70%) resorption (reduction in polymer mass) at one year. In studies with the DESolve Scaffold, the scaffold thrombosis rates have been very low and the long-term clinical results excellent.[18]

However, bioresorbable scaffolds have fallen out of favor to a certain degree as they demand more rigorous pre-dilatation, scaffold sizing and post-dilatation techniques. Therefore, Elixir Medical has designed the next generation of DESyne and DESyne BD stents incorporating expansion segments into the bioadaptor pattern. The expansion segments are designed to disengage in approximately 6 months to allow bioadaptive remodeling and restore more normal vessel movement (expansion and contraction) in the implanted region similar to the DESolve bioresorbable scaffold, thereby performing like a metallic DES for vessel support with the benefit of performing like the DESolve scaffold for vessel motion. The DynamX Sirolimus Eluting Coronary Bioadaptor System (DynamX SECBS) is being evaluated in this clinical study

1.1.1 Novolimus and Biodegradable Polymer Coating in Clinical Studies

The safety and efficacy of Novolimus (metabolite of sirolimus) on a drug eluting metallic stent was demonstrated in six larger studies; two randomized clinical trials and three registries enrolling a total of 466 patients. The devices evaluated were the Elixir DESyne Novolimus Eluting Coronary Stent System, the DESyne BD Novolimus Eluting Coronary Stent System or the DESolve Novolimus Eluting Coronary Scaffold System; all of these devices have received CE Mark approval. The dose of Novolimus was the same for all devices (approx. 5mcg per mm of stent length). The polymer topcoat of a poly-lactide based polymer being used on the ELX1805J is the same as used in the EXCELLA II Study Phase 2 and DESolve Nx Study.

1.1.2 EXCELLA II Phase 1 Randomized Control Study Summary

The EXCELLA II Randomized Clinical Trial (RCT) was a multi-center, randomized clinical trial designed to demonstrate non-inferiority of the DESyne NECSS to a control, the commercially available Medtronic Endeavor ZECSS. This trial enrolled 210 patients in a 2:1 randomization scheme who met the inclusion and exclusion criteria. Patients were recruited from 21 sites in Europe (Belgium, Germany, Poland, and the Netherlands), New Zealand and Australia. The primary endpoint of the study was the in-stent late lumen loss at 9 months. Key secondary endpoints included the Device-oriented Composite Endpoints (DoCE), defined as cardiac death, myocardial infarction (MI) not clearly attributable to a non-intervention vessel, and clinically-indicated target lesion revascularization, at 1, 6, 9, and 12 months and annually to 5 years. Intravascular ultrasound (IVUS) in a sub-set of patients included an assessment of the in-stent volumetric

neointimal burden and percent volume obstruction. An assessment of stent thrombosis was included in accordance with the ARC definitions.[19]

Angiographic data at 9 months were available in 186 patients (204 lesions). The Reference Vessel Diameter (RVD), MLD and %DS pre- and post-procedure were all comparable between both study arms. At 9 months, the primary endpoint of mean in-stent LLL was significantly lower for DESyne NECSS compared to the Endeavor ZECSS, (0.11 ± 0.32 mm vs. 0.63 ± 0.42 mm, non-inferiority $p < 0.0001$, superiority $p < 0.0001$).

IVUS was conducted in a subset of 65 patients. A total of 62 patients were analyzed (40 DESyne and 22 Endeavor) at baseline including 70 lesions (46 DESyne and 24 Endeavor). Between baseline and follow-up in this subset, patients treated with the Endeavor Stent had a significantly larger vessel volume index at follow-up indicating more neointimal hyperplasia. There were no cases of late acquired incomplete stent apposition for either stent.

Hierarchical and non-hierarchical clinical outcomes through 270 days, which represents the primary endpoint follow-up period, showed no significant difference between stent groups in the device orientated composite endpoint (DESyne NECSS 2.9% vs. Endeavor ZECSS 5.6%, (-2.8% [-8.8%, 3.3%], p = 0.45) or its individual components of cardiac death, target vessel MI and CI-TLR. Clinical follow-up was performed annually from 1 – 5 years and results are summarized in **Table 1**.

In summary, this non-inferiority randomized study not only met its primary endpoint, but also demonstrated superiority of the DESyne NECSS as compared to the Endeavor ZECSS in terms of in-stent late loss (0.11 ± 0.32 mm vs. 0.63 ± 0.42 mm, non-inferiority $p < 0.0001$, superiority $p < 0.0001$). Although the study was not powered to demonstrate statistical differences in clinical events between the test and control arms, there was a significant reduction in DoCE for the DESyne stent as compared to the Endeavor stent at 60 months.. These long-term clinical follow-up data demonstrate the safety of the DESyne stent.

Table 1: EXCELLA II Device Oriented Composite Endpoint through 60 Months (1770 days)

	DESyne (N = 139 pt)	Endeavor (N = 71 pt)	P-value
**Hierarchical Subject Counts**			
**DoCE**	7.9% (11/139)	19.7% (14/71)	0.02
**Cardiac Death**	2.9% (4/139)	4.2% (3/71)	0.69
**Target Vessel MI**	2.9% (4/139)	7.0% (5/71)	0.170
**Q-wave**	2.2% (3/139)	1.4% (1/71)	1.00
**Non Q-wave**	0.7% (1/139)	5.6% (4/71)	0.05
**CI TLR**	2.2% (3/139)	8.5% (6/71)	0.06
**CABG**	0.0% (0/139)	2.8% (2/71)	0.11
**PCI**	2.2% (3/139)	5.6% (4/71)	0.23
**Stent Thrombosis***			
**- Definite**	0.7% (1/139)	1.4% (1/71)	1.00
**- Probable**	1.4% (2/139)	0.0% (0/71)	1.00
**- Definite + Probable**	2.2% (3/139)	1.4% (1/71)	1.00

Note: Numbers are % (counts/sample size).
*Stent Thrombosis definitions:
Definite stent thrombosis is considered to have occurred by either angiographic or pathological confirmation;
Probable stent thrombosis is considered to have occurred after intracoronary stenting in the following cases:

- cardiac and unexplained death within the first 30 days,
- irrespective of the time after the index procedure, MI (target or non-target vessel) that is related to documented acute ischemia in the territory of the implanted stent without angiographic confirmation of stent thrombosis and in the absence of any other obvious cause

This study was followed by a Continued Access Enrollment and a Post Market Clinical Follow-up (PMCF) study, which were confirmatory multi-center, non-randomized studies designed to provide additional clinical-only evaluation of the DESyne Stent. The EXCELLA Continued Access Enrollment enrolled 99 patients between April 2009 and October 2009 with all patients receiving the DESyne Stent. Patients were recruited from 6 sites in Europe and Brazil. The primary endpoint of the study was the Device-oriented Composite Endpoints (DoCE), defined as cardiac death, MI not clearly attributable to a non-intervention vessel, and clinically-indicated target lesion revascularization, at 1, 6, 9, and 12 months and annually to 5 years. An assessment of stent thrombosis was included in accordance with the ARC definitions. Clinical follow-up was based on an intention-to-treat basis. The EXCELLA PMCF Study enrolled 57 patients between February 2014 and May 2014 with all patients receiving the DESyne Stent. Patients were recruited from 4 sites in Jordan and Europe. The primary endpoint of the study was the Device-oriented Composite Endpoints (DoCE), defined as cardiac death, MI not clearly attributable to a non-intervention vessel, and clinically-indicated target lesion revascularization, at 1, 9, 12, and 24 months. An assessment of stent thrombosis was included in accordance with the ARC definitions. The results of both studies are summarized in Table 2. Overall, clinical events were low and similar to the EXCELLA II Phase 1 randomized cohort of patients.

Table 2: Summary of Clinical Events for EXCELLA Continued Access and EXCELLA PMCF – DESyne NECSS

Event	9 months	12 months	24 months	36 months	48 months	60 months
**EXCELLA Continued Access**						
Cumulative DoCE	2.0% (2/99)	3.0% (3/99)	4.0% (4/99)	5.1% (5/99)	6.1% (6/99)	7.1% (7/99)
Cardiac Death	0.0% (0/99)	0.0% (0/99)	0.0% (0/99)	1.0% (1/99)	2.0% (2/99)	2.0% (2/99)
Target Vessel MI	1.0% (1/99)	1.0% (1/99)	1.0% (1/99)	1.0% (1/99)	1.0% (1/99)	1.0% (1/99)
CI TLR	1.0% (1/99)	2.0% (2/99)	3.0% (3/99)	3.0% (3/99)	3.0% (3/99)	4.0% (4/99)
Stent Thrombosis (Definite)	0.0% (0/99)	0.0% (0/99)	0.0% (0/99)	0.0% (0/99)	1.0% (1/99)	1.0% (1/99)
**EXCELLA PMCF (Hierarchical Events)**						
DoCE	1.8% (1/57)	1.8% (1/57)	5.3% (3/57)	--	--	--
Cardiac Death	0.0% (0/57)	0.0% (0/57)	3.5% (2/57	--	--	--
Target Vessel MI	0.0% (0/57)	0.0% (0/57)	0.0% (0/57)	--	--	--
CI- TLR	1.8% (1/57)	1.8% (1/57)	1.8% (1/57)	--	--	--
Stent Thrombosis (Definite)	0.0% (0/57)	0.0% (0/57)	0.0% (0/57)	--	--	--

1.1.3 EXCELLA II Phase 2 Randomized Control Study Summary

The EXCELLA II Phase 2 RCT was a multi-center, randomized clinical trial designed to demonstrate non-inferiority of the DESyne BD NECSS to a control, the commercialized Medtronic Endeavor ZECSS. The EXCELLA II Phase 2 study randomized 151 patients to treatment with DESyne BD NECSS (n = 120) and Endeavor ZECSS (n = 31) between 10 September 2010 and 20 January 2011. Patients were recruited from 10 sites in Belgium, Germany, Poland, and Brazil. There were two patients who were deregistered following randomization due to failure to meet the angiographic inclusion and exclusion criteria. Three additional patients withdrew their consent to participate further in the study; the data from these patients was therefore not used for any analysis. Thus clinical data from a total of 146 patients is was available for analysis: 115 patients in the DESyne BD group and 31 in the Endeavor group.

Patients were analyzed for the primary endpoint of in-stent late lumen loss assessed by QCA at 6 months as well as the secondary endpoints including: Device-orientated Composite Endpoints (DoCE) defined as: cardiac death, myocardial infarction (MI) not clearly attributable to a non-intervention vessel, and clinically-indicated target lesion revascularization at 1, 6, 9, and 12 months and annually through 5 years, clinically-indicated Target Lesion Revascularization (TLR), and clinically-indicated Target Vessel Revascularization (TVR) as well as other clinical and imaging endpoints. A subset of patients underwent IVUS evaluation including in-stent volumetric neointimal burden and percent (%) neointimal obstruction at 6 months.

Angiographic data at baseline were available on 112 patient (127 lesions) for the DESyne BD group and 31 patients (38 lesions) for the Endeavor group. Angiographic data at 6 months were available in 107 patients (119 lesions) for the DESyne BD arm and 31 patients (38 lesions) in the Endeavor arm. At 6 months, the primary endpoint of mean in-stent LLL was significantly lower for the DESyne BD NECSS compared to the Endeavor ZECSS (0.12 ± 0.15 mm vs. 0.67 ± 0.47 mm). Based on the QCA data, the DESyne BD Stent is considered non-inferior and superior (non-inferiority $p < 0.001$, superiority $p < 0.001$) to the control Endeavor Stent thus meeting the study primary endpoint of in-stent late lumen loss.

Intravascular ultrasound imaging was performed at baseline and at the 6-month follow-up on a subset of 51 lesions. The percent neointimal volume at 6 months was $3.6 \pm 4.2\%$ for the DESyne BD stent group and $20.7 \pm 14.2\%$ for the Endeavor stent group with a p-value of < 0.001. There were 2 minor late Incomplete stent apposition (ISA) in the DESyne BD group and zero for the Endeavor group ($p = 0.99$).

Hierarchical clinical outcomes 60 months showed no significant difference between study arms in the modified intent to treat (MITT) (defined as those patients receiving a study stent) and are summarized in **Table3**.

This non-inferiority randomized study not only met the primary endpoint, but also demonstrated superiority of the DESyne BD NECSS as compared to the Endeavor ZECSS in terms of in-stent late loss. In addition, the rates of adverse cardiac events were low and comparable between both stents through the entire 60-month study. These long-term clinical follow-up data support the safety of the DESyne BD stent.

Table 3: EXCELLA II Phase 2 Device Oriented Composite Endpoint and Stent Thrombosis at 60 Months (MITT set)

Characteristic	DESyne BD (n = 112)	Endeavor (n = 31)	P value
**Hierarchical DoCE**	8.0% (9/112)	9.7% (3/31)	0.72
**Cardiac Death**	2.7% (3/112)	0.0% (0/31)	> 0.99
**MI (target vessel)**	0.9% (1/112)	0.0% (0/31)	> 0.99
**Q-wave**	0.0% (0/112)	0.0% (0/31)	No P-value
**Non Q-wave**	0.9% (1/112)	0.0% (0/31)	> 0.99
**CI TLR**	4.5% (5/112)	9.7% (3/31)	0.37
**Stent Thrombosis***	0.0% (0/112)	0.0% (0/31)	No P-value
**Definite**	0.0% (0/112)	0.0% (0/31)	No P-value
**Probable**	0.0% (0/112)	0.0% (0/31)	No P-value

1.1.4 DESolve Nx Study Summary

The DESolve Nx study was a multi-center, clinical trial designed to demonstrate the safety and performance of the DESolve NEBCSS. Patients were analyzed for the principal effectiveness endpoint of in-scaffold late lumen loss assessed by QCA at 6 months as well as the primary safety endpoint of Major Adverse Cardiac Events (MACE) defined as: cardiac death, myocardial infarction (MI) not clearly attributable to a non-intervention vessel, and clinically-indicated target lesion revascularization through 6 months. Additional secondary endpoints include MACE, clinically-indicated Target Lesion Revascularization (TLR), and clinically-indicated Target Vessel Revascularization (TVR) assessed at 1, 6, 9, and 12 months and annually through 5 years. Lesions were evaluated for angiographic endpoints at 6 months which included: in-lesion late lumen loss (LLL), percent diameter stenosis (% DS), minimal lumen diameter (MLD) post procedure and at 6 months, angiographic binary restenosis (≥ 50%), and stent thrombosis (ST).

In a subset of patients, additional imaging endpoints using multiple modalities were evaluated. IVUS evaluation at baseline and follow-up included in-scaffold volumetric neointimal burden and percent (%) neointimal obstruction at baseline and 6 months. Additionally, Optical Coherence Tomography (OCT) descriptive analysis of lesion/vessel morphometry and scaffold strut composition in patients was performed at baseline and 6 months. This subset of patients also underwent imaging analysis at 12 months using multi-slice computed tomography (MSCT). In two separate, single center sub-studies, patients underwent imaging follow-up (angiography, IVUS and OCT at 18 and 36 months to demonstrate the scaffold/lesion characteristics at longer-term follow-up.

Results

The DESolve Nx study enrolled 126 patients between 2 November 2011 and 14 June 2012. Clinical data are available on 126 patients. Angiographic imaging data are available at follow-up on 113/126 patients; IVUS and OCT data are available on 40 and 38 patient subsets respectively and are based on paired and include only those patients receiving a study device. Baseline patient demographics and angiographic characteristics are typical for a study of this nature including the average patient age of 62 years and percentage of male patients (68.3%).

Imaging Results

Angiographic data at baseline and 6-month follow-up were available on 126 and 113 patients respectively. At the 6-month follow-up, the mean in-scaffold late lumen loss (LLL) was 0.20 ± 0.32 mm and in-segment (LLL) 0.21 ± 0.31 mm.

Paired intravascular ultrasound imaging analyzed at baseline and at the 6-month follow-up was available in 40 patients. At 6 months, the neointimal % volume obstruction, which

indicates the amount of neointimal hyperplasia along the length the scaffold, was 5.13 ± 4.19%. There were no cases of late malapposition in the paired analysis. Importantly, the IVUS data demonstrated both significant lumen and scaffold area growth at 6 months indicating initial vascular restoration. The lumen area change from baseline to 6-month follow-up was 5.93 mm^2 to 6.51 mm^2 and the scaffold area change from baseline to 6-month follow-up was 5.94 mm^2 to 6.87 mm^2 both were statistically significant ($p < 0.001$).

Paired optical coherence tomography (OCT) imaging analyzed at baseline and at the 6-month follow-up was available in 38 subset patients. At 6 months, the frequency of covered struts per patient was 98.79 ± 1.69% demonstrating excellent scaffold coverage by neointima. The analysis of this neointimal coverage of the struts showed a mean neointimal thickness of 0.10 ± 0.03 mm. Similar to the IVUS results, there was evidence of significant scaffold growth between baseline and the 6 month follow-up from 7.04 mm^2 to 8.17 mm^2 ($p < 0.001$).

Multi-slice computed tomography (MSCT) was performed at 12 months and was available in 41 subset patients. The average minimum in-scaffold lumen diameter at 12 months was 2.2 ± 0.4 mm and the minimum lumen area was 4.3 ± 1.4 mm^2 and showed good patency of the treated segments.

At 18 months, a single center in Brazil performed follow-up in the majority of the enrolled patients who were requested to undergo imaging follow-up by angiography, IVUS and OCT. A total of 19/28 patients returned for angiography, 18/28 patients underwent IVUS and 20/28 patients underwent OCT imaging. In these patients, the late lumen loss was numerically similar albeit statistically different to the data at 6 months (0.23 ± 0.33 vs. 0.29 ± 0.34 mm $p = 0.01$). By IVUS, the mean lumen gain seen at 6 months was maintained at 18m however, strut level analysis was not possible as the struts were no visible by IVUS. Finally by OCT, virtually all struts were covered by a thin layer of neointima (99.98% coverage and 0.2 ± 0.05 μm neointimal thickness).

At 36 months, a single center in Belgium performed follow-up in the majority of the enrolled patients who were requested to undergo imaging follow-up by angiography, IVUS and OCT. A total of 19/22 patients returned for angiography, 18/28 patients underwent IVUS and 20/28 patients underwent OCT imaging. In these patients, the paired late lumen loss was statistically similar to the data at 6 months (0.12 ± 0.14 vs. 0.22 ± 0.33 mm; $p = 0.20$). By IVUS, the mean lumen gain seen at 6 months was still maintained at 36m however, strut level analysis was not possible as the struts were not visible by IVUS. Finally by OCT, struts were no longer visible by OCT and the vessel showed no signs of the scaffold.

Clinical Results

The hierarchical composite clinical endpoint of MACE through 60 months for the modified intent-to-treat (MITT) group, inclusive of those patients receiving a study scaffold in the target lesion, is summarized in **Table 4**. Of note, there was no acute, sub-acute, late or very late definite scaffold thrombosis reported for the entirety of the study follow-up period.

Table 4: DESolve Nx Clinical Outcomes

Hierarchical Events 0 to 1800 days, n (%)	6 Months (N = 122)*	12 Months (N = 122)*	24 Months (N = 122)*	36 Months (N = 122)*	48 Months (N = 122)*	60 Months (N = 122)*
**Major Adverse Cardiac Events**	4 (3.3%)	7 (5.7%)	9 (7.4%)	10 (8.2%)	11 (9.0%)	11 (9.0%)
**Cardiac Death****	1 (0.8%)	2 (1.6%)	3 (2.5%)	4 (3.3%)	4 (3.3%)	4 (3.3%)
**Target vessel MI*****	1 (0.8%)	1 (0.8%)	1 (0.8%)	1 (0.8%)	2 (1.6%)	2 (1.6%)
**Q-wave MI**	0 (0.0%)	0 (0.0%)	0 (0.0%)	0 (0.0%)	0 (0.0%)	0 (0.0%)
**Non-Q-wave MI**	1 (0.8%)	1 (0.8%)	1 (0.8%)	1 (0.8%)	2 (1.6%)	2 (1.6%)
**Clinically Indicated TLR**	2 (1.6%)	4 (3.3%)	5 (4.1%)	5 (4.1%)	5 (4.1%)	5 (4.1%)
**Definite Stent Thrombosis**[+]	0 (0.0%)	0 (0.0%)	0 (0.0%)	0 (0.0%)	0 (0.0%)	0 (0.0%)

* Modified Intent to Treat = those patients in which a scaffold was implanted in target lesion
**One death with probable ST based on ARC, scaffold undersized as assessed by IVUS; one death with suspected pulmonary embolus with right heart failure, non-scaffold related; one death due to non-target vessel occlusion and PCI, non-scaffold related
***MI during follow up attributed to multi-modality imaging procedure
+ ARC-defined

The DESolve Nx pivotal trial was successful in demonstrating safety and efficacy of the DESolve Scaffold. In addition, the DESolve scaffold demonstrated lumen and scaffold growth at 6 months by IVUS. At 18 and 36 months, these excellent results were maintained with continued neointimal suppression, and at 36 months completed resorption of the scaffold as evidenced by OCT imaging. The DESolve scaffold, results demonstrated a low 60-month MACE rate at 9.0% with no reported definite scaffold thrombosis through 5 years.

This study was followed by a Post Market Clinical Follow-up (PMCF) study, which was a confirmatory multi-center, non-randomized study designed to provide additional clinical-only evaluation of the DESolve scaffold. Additionally, a post market study was also conducted on a thinner (120 µm) version of the DESolve scaffold, the DESolve Cx scaffold. The primary endpoint of both studies was MACE, defined as cardiac death, MI not clearly attributable to a non-intervention vessel, and clinically-indicated target lesion revascularization at follow-up. Follow-up was scheduled for 1, 6, and 12 months and annually for a minimum of 3 years. The DESolve PMCF trial enrolled 100 patients with all patients receiving the DESolve Scaffold. Patients were recruited from 10 sites in Germany and Italy. The DESolve Cx trial has enrolled 50 patients with all patients receiving the DESolve Cx Scaffold. Patients were recruited from 3 sites in Belgium and Brazil. Clinical results from both studies are summarized in **Table 5**.

Table 5: Summary of Clinical Events for DESolve PMCF and DESolve Cx Trials

Hierarchical Events	30 Days	6 Months	12 Months	24 Months
**DESolve PMCF**				
MACE	1.0% (1/100)	2.0% (2/100)	3.0% (3/100)	4.0% (4/100)
Cardiac Death	0.0% (0/100)	0.0% (0/100)	0.0% (0/100)	0.0% (0.0%)
MI	1.0% (1/100)	1.0% (1/100)	1.0% (1/100)	1.0% (1/100)
TLR	0.0% (0/100)	1.0% (1/100)	2.0% (2/100)	3.0% (3/100)
Definite ST†*	1.0% (1/100)	1.0% (1/100)	1.0% (1/100)	1.0% (1/100)
**DESolve Cx**				
MACE	0.0% (0/50)	0.0% (0/50)	0.0% (0/50)	Pending
Cardiac Death	0.0% (0/50)	0.0% (0/50)	0.0% (0/50)	Pending
MI	0.0% (0/50)	0.0% (0/50)	0.0% (0/50)	Pending
TLR	0.0% (0/50)	0.0% (0/50)	0.0% (0/50)	Pending
Definite ST†*	0.0% (0/50)	0.0% (0/50)	0.0% (0/50)	Pending

† In accordance with ARC definition

* Incomplete coverage of a proximal lesion resulting in acute closure

These results are consistent with the results from the DESolve Nx study.

1.1.5 DynamX Novolimus Eluting Coronary Bioadaptor System Clinical Experience

The DynamX Novolimus Eluting Coronary Bioadaptor System is currently being evaluated in a clinical study designed to enroll up to 50 patients in Europe requiring treatment of a single, de novo lesion ≤ 24 mm in length located in a vessel > 2.5 mm and ≤ 3.5 mm in diameter. Clinical follow-up will be conducted in all patients at 30 days and 6 months, and annually through 3 years. All patients will have the baseline angiography procedure analyzed. Two imaging sub-studies will be conducted with imaging follow-up at 6 months or 9 months. The principal endpoint of the study is TLF at 6 months. Enrollment has completed and 2 year clinical and imaging follow-ups are underway with the first few patients showing excellent bioadaptor patency and no reported bioadaptor-related clinical events.

1.2 Non-Clinical Studies

ELX1805J System has been evaluated in non-clinical safety studies including *in vivo* animal studies using pig coronary artery model. The studies have shown that ELX1805J and delivery system were safe and reliable and that they could be used clinically. Other non-clinical studies including *in vivo* animal studies using pig coronary artery model have been performed with the DynamX Novolimus Eluting Coronary Bioadaptor System and the Cypher Sirolimus Eluting Coronary Stent System, which have similarities with the ELX1805J System, providing additional support of the safety of the study device. The results of non-clinical studies of Sirolimus and the bioadaptor are described in the investigator's brochure.

1.3 Risk Assessment

The risks associated with the use of the ELX1805J SECBS have been assessed. There is publically available *in vivo* and clinical information regarding the safety and the known mechanism of action of the drug Sirolimus supporting the safety of its use in drug eluting coronary stents. Additionally biocompatibility testing of the components of the bioadaptor and biodegradable polymer coatings demonstrate safety. In vitro device verification testing of the ELX1805J has also demonstrated safety of the device per industry standard drug eluting stent testing. Non-clinical studies with the ELX1805J SECBS and similar devices have demonstrated a safe and effective method for reducing neointimal hyperplasia in the animal model and clinical setting. Multicenter studies including the EXCELLA II Phase 1, EXCELLA II Phase 2 and DESolve Nx pivotal clinical

trials support the safety and efficacy of the ELX1805J. Further, DESyne Novolimus Eluting Coronary Stents and DESolve family of scaffolds are commercially available and distributed globally in many countries, including members of the European Union.   The data from the in vivo and in vitro testing and clinical studies are described in the investigator's brochure.   These data therefore suggest that the ELX1805J Bioadaptor is safe for use in the Bioadaptor Study.

2. Study Objective

The objective of this study is to verify efficacy and safety of investigational device (ELX1805J) for ischemic heart diseases caused by de novo stenotic lesion(s) in a native coronary artery. In this study, the Resolute Onyx family of drug-eluting stents is used as control device.

3. Clinical Study Plan

3.1 Type of Study

Confirmatory study

3.2 Study Design

3.2.1 Multicenter Randomized Single-blind Study (referred to as the Bioadaptor Study)

It is allowed to treat up to 2 *de novo* lesions located in 2 separate native coronary arteries as target lesion(s), and the study is designed to randomly allocate 222 subjects in a 1:1 ratio (test:control) . An additional 222 subjects from the European Bioadaptor Studies will be pooled for the primary endpoint. The analysis population consists of all randomized patients in the Bioadaptor RCT study and the first 222 randomized patients enrolled in the European Bioadaptor Studies that meet inclusion/exclusion criteria of the Bioadaptor RCT.

In addition, it is allowed to treat one lesion in a non-target vessel using device other than study device as non-target lesion, and it is required to treat non-target lesion and achieve lesion success before initiation of treatment of target lesion. Subjects receive follow-up assessments at 1, 6 and 12 months and every year for 5 years thereafter.   **Note:** When treating a non-target lesion with a DES, the DES shall have an "olimus" type of drug coating.

3.2.2 Single-group PK Study (referred to as the PK study)

It is allowed to treat up to 2 de novo lesions located in 2 separate native coronary arteries as target lesion, and the study is allocated 8 subjects. PK study is conducted to assess blood pharmacokinetics of Sirolimus eluted from ELX 1805J Bioadaptor implanted in Japanese patients. PK measurement is conducted at pre-treatment, 10 minutes, 30 minutes, 1, 2, 4, 6, 12, 24, 72 hours and 7 days. In addition, all subjects receive follow-up assessments at 1, 6 and 12 months and every year for 5 years thereafter.

3.3 Efficacy Endpoints and Safety Endpoints

For endpoints, evaluation index for coronary stent system or evaluation index for prognosis of treatment of coronary artery disease are specified as described below. Evaluation indices of the clinical study previously conducted and "Handling of application for approval of coronary stent" (PFSB/ELD Notification No. 0904001, issued on September 4, 2003) were referred.

3.4 Primary Endpoint

3.4.1 Primary Endpoint of Multicenter Randomized Single-blind Study

TLF Rate at 12 months after study procedure, [Rationale]

TLF is a composite rate defined as "cardiovascular death, myocardial infarction (MI: Q-wave and non-Q-wave) associated with target vessel, and CI-TLR requiring percutaneous intervention (PCI) or coronary artery bypass graft (CABG)" and is useful for overall assessment of clinical symptoms of patients. It is standard practice and a commonly used method in clinical studies to designate TLF as the primary endpoint for evaluation of new coronary stent systems. Since TLF is the most direct and important endpoint for confirmation of non-inferiority in comparison of DES and drug-eluting bioresorbable scaffold, TLF is defined as the primary endpoint for this study.

3.4.2 Primary endpoint of PK study

Characterization of the pharmacokinetic profile (e.g., Cmax, AUC, $T_{1/2}$)

[Rationale]
This endpoint is defined to analyze pharmacokinetic parameters of ELX1805J.

3.5 Secondary Endpoint

3.5.1 Secondary Endpoints of multicenter randomized single-blind study

(1) Efficacy endpoints

- Acute success rates:
  Lesion success rate, device success rate, procedure success rate

(2) Imaging endpoints

QCA endpoints:

- Acute recoil
- Late lumen loss (in-stent and in-segment) at 12-month follow-up
- Change in vessel angulation from baseline, post-stent and 12-month follow-up
- MLD post-procedure and 12 months
- % DS post-procedure and 12 months

IVUS endpoints:

- Change in mean lumen area from post-procedure to 12-month follow-up
- In-stent % neointimal obstruction at 12-month follow-up
- In-stent late lumen loss at 12-month follow-up
- Acute, persistent and late stent malapposition

OCT endpoints:

- % Strut coverage
- Neointimal thickness
- Vessel Pulsatility - % change in Lumen Area and Device Area during systole and diastole by stationary OCT
- Additional parameters may be assessed

(3) Clinical endpoints:

Measured at 30 days, 6 months, 12 months, 2, 3, 4 and 5 years:

- TLF
- Patient Oriented Clinical Endpoint: Overall cardiovascular outcomes from the patient's perspective. This endpoint is a composite endpoint that includes all-cause mortality (cardiac and non-cardiac), stroke, MI (target vessel and non-target vessel), and revascularization (target vessel and non-target vessel)
- A composite of all-cause mortality, MI (target vessel or non-target vessel) and revascularization (target vessel and non-target vessel)
- Composite of cardiovascular death, target vessel myocardial infarction (TV-MI)*, or clinically-indicated target vessel revascularization (CI-TVR)
- Composite of cardiovascular death, stroke, MI (target vessel and non-target vessel) and revascularization (target vessel and non-target vessel)
- Composite of cardiac death, MI (target vessel and non-target vessel) and revascularization (target vessel and non-target vessel)
- CI-TLR
- TLR
- Target vessel revascularization (TVR)
- Clinically-indicated TVR (CI-TVR)
- Revascularization(target vessel and non-target vessel)
- Q-wave MI
- Non Q-wave MI
- MI (target vessel and non-target vessel)
- Target vessel MI
- Cardiovasculardeath
- All-cause death
- Composite of cardiac death or target vessel MI
- Composite of all-cause death or MI (target vessel and non-target vessel)
- Composite of all-cause death, MI (target vessel and non-target vessel), or TVR
- Composite of probable or definite stent thrombosis‡
- Probable stent thrombosis‡
- Definite stent thrombosis‡

** Defined as myocardial infarction not clearly attributed to a non-target vessel*
‡ Defined as per the Academic Research Consortium (ARC-2) criteria

[Rationale]
Acute success rates (lesion success rate, device success rate, procedure success rate) were selected since they are indices to indicate initial therapeutic effect of implantation of study devices. Angiographic and IVUS endpoints are indices to indicate long-term therapeutic effect of implantation of the study devices. In addition, Death, MI, TLR, TVR, and TVF/CI-TVR were selected since they indicate therapeutic effect of the study devices and prognosis after implantation of the study devices.

(4) Safety endpoints

Verification of endpoints at 1, 6 and 12 months after study procedure and every year until 5 years after study procedure

- Device/stent thrombosis rate (for ELX1805J and Resolute Onyx)
- Adverse events, serious adverse events, malfunctions
- All cardiovascular events regardless of seriousness or device relationship
- All study-device related events and events for which the relationship to the study device is unknown
- All unanticipated adverse device effects
- All CVAs (cerebrovascular accident)

Note: Primary assessment of device/stent thrombosis is conducted using the ARC-2 definition. Thrombosis associated with MI is evaluated using only the per protocol definition of MI. For definitions, see (2) Terms of "I. Definition of Terms" (page 4).

[Rationale]
Since device thrombosis relates to occurrence of coronary artery diseases and indicates prognosis after implantation of Bioadaptor/stent, this is defined as the safety endpoint. Adverse events and serious adverse events are selected as safety endpoints to evaluate safety of the clinical study comprehensively. In addition, malfunctions were selected as safety endpoints to evaluate events that could be caused by study devices.

In addition, these endpoints are listed as the items to be evaluated in "Handling of application for approval of coronary stent" (PFSB/ELD Notification No. 0904001, issued on September 4, 2003).

3.5.2 Secondary Endpoints of PK study

Pharmacokinetic parameters at multiple time points after study procedures

3.6 Number of Subjects and Rationale

3.6.1 Number of subjects and rationale of Multicenter randomized single-blind study

The null hypothesis for this study is that the ELX1805J arm will have a 12-month TLF rate that exceeds that of the control stent arm by at least a pre-specified margin of δ (delta). The alternative hypothesis is that the ELX1805J arm will have a 12-month TLF rate that is no more than that of the control stent, or exceeds that of the control stent but by less than δ. Rejection of the null hypothesis indicates non-inferiority of investigational device as compared to control device for TLF rate at 12 months.

To calculate the appropriate rate of TLF, the Sponsor has investigated using peer reviewed journals, the results of clinical studies in previous approved DES and drug eluting bioresorbable scaffolds. Based on these results and proposed inclusion/exclusion criteria of this study, the TLF Rate at 12 months after study procedure is 9.0% for both the ELX1805J arm and the control arm, and non-inferiority margin of δ is 8.6%

Specifically, the null hypothesis (H_o) and the alternative hypothesis (H_a) can be expressed as below:

H_o: $\pi_A \geq \pi_C + \delta$ H_o: $\pi_A \geq \pi_C + 8.6\%$
H_a: $\pi_A < \pi_C + \delta$ H_a: $\pi_A < \pi_C + 8.6\%$

Here, π_A is a true TLF Rate of investigational device, and π_C is a true TLF Rate of control device.

To have a power of 90% in test for non-inferiority for endpoints that follows binomial distribution, a total of 400 patients are required. Tests are conducted using normal approximation of binomial distribution.

Assuming that the rate of lost to follow-up for 12 months after study procedure is 10%, a total number of subjects required is 444 subjects with 222 subjects in investigational device group and 222 subjects in control device group.

3.6.2 Number of subjects and rationale of Single-group PK study

8 subjects were defined as number of subjects to achieve the objective for the PK study.

3.7 Methods for Randomization and Blinding

3.7.1 Randomization

For randomization, block randomization using random block sizes is used, and subjects are stratified at each medical institution. Randomization will be done after successful treatment of the non-target lesion (if any) and successful pre-dilatation of the target lesion (or the first target lesion if there are two target lesions) and vessel sizing.

The subject is considered to be successfully enrolled in this study and considered in the intent-to-treat (ITT)population at the point of randomization. ITT population consists of subjects randomized regardless of conduct of implantation of study device. These subjects will be followed for the duration of the study.

The population evaluable for treatment (Per-Treatment Evaluable: PTE) consists of subjects that received implantation of study device only without any serious deviation from the protocol (implantation in left main trunk, stenting in saphenous veins graft, acute myocardial infarction, etc.).

3.7.2 Method of Blinding

This clinical study is single-blind study.
Since the investigational device is different from control device in its shape and visibly distinguishable, the primary investigator, subinvestigator, clinical research coordinator and study device manager cannot be blinded.

On the other hand, for subjects, it is stated not to disclose allocated device in the briefing document, and subjects are blinded by not informing them about allocated device. In addition, source medical records should refer to the “Study Stent”, and the CEC will adjudicate all events, blinded to the device deployed. Information regarding the use of either the study or the control device for treatment and that they will not be informed of the used device is described in the Informed Consent.

3.7.3 Control and Storage of Records of Randomization

Records of randomization are controlled and stored by the person responsible for allocation and enrollment at the subject enrollment center independent from the sponsor and medical institution.

3.7.4 Control Procedure for Emergency Key Codes

Since the sponsor, primary investigator, subinvestigator, clinical research coordinator and study device manager are not blinded in this clinical study, emergency key is not generated.

4. Selection of Subjects

4.1 Consent of Subjects

4.1.1 Method for Informed Consent

Once the patient is determined to be eligible for this clinical study, the primary investigator or subinvestigator provides the patient with an explanation on the background, treatment, benefits, disadvantages, etc. of this clinical study. Only patients to whom explanation is provided using the informed consent document approved by institutional review board and from whom consent is obtained in writing prior to participation in clinical study may participate. Patients for whom consent is not obtained in writing are considered ineligible

for this clinical study. Only if patients cannot write informed consent document, it is possible to be signed by designated legal representative.

Subjects for whom this consent procedure was conducted are listed in the screening list.

(1) Primary investigator or subinvestigator adequately explains the items listed in "4.1.2 Contents of Informed Consent Document" below to patient using the informed consent document. Sufficient time to consider participation in clinical study should be given, and after verification that patient understand its contents, consent for participation in clinical study is obtained in writing with free will of patient.

(2) On the informed consent form, both primary investigator or subinvestigator who provided explanation to patient and patient who received explanation sign and seal or sign and date. In addition, when clinical research coordinator provided supplementary explanation, the clinical research coordinator concerned signs and seals or signs and dates.

(3) A duplicate copy of signed and sealed or signed and dated informed consent form and informed consent document are given to subject, and the original copy of informed consent form is retained by primary investigator or subinvestigator in accordance with the regulation of each medical institution.

(4) Upon obtainment of consent from subject, primary investigator or subinvestigator enters date of consent and subject identification code on the subject enrollment document (subject screening list).

(5) When primary investigator or subinvestigator obtains any information that may affect subject's decision on continuation of participation in clinical study, he/she promptly conveys the information concerned to subject, confirms willingness for continuous participation in clinical study and records the fact that the information concerned is provided and decision of subject on medical record. If there is a method specified by medical institution, the method is followed.

4.1.2 Contents of Informed Consent Document

(1) Statement describing: Clinical study involves research.
(2) Study Objective

(3) Name, title, contact information of primary investigator

(4) Study method (details of conduct of clinical study, inclusion/exclusion criteria for subjects, randomization, blinding to treatment device).

(5) Expected effects of study device for health of subjects (if effects of study device can`t be expected, its content should be described) and expected disadvantages for subject

(6) Items related to other therapies

(7) Expected duration of the subject's participation in the study

(8) Subject can refuse or withdraw participation to clinical study at any time

(9) Refusal or withdrawal of consent does not result in any disadvantageous treatment of subject or loss of benefits that subject could receive with participation in clinical study

(10) Handling of study device in case of withdrawal from clinical study after participation to clinical study

(11) Clinical research associate, auditor, institutional review board and regulatory authority etc. are allowed to access source medical records under condition of that confidentiality of subject is protected

(12) Confidentiality of subject is protected

(13) Contact information at medical institution in case of health damage

(14) When health damage occurs, proper treatment is conducted for subject

(15) Items related to compensation for health damage

(16) Type of institutional review board that reviews and evaluates suitability of clinical study, items of review and evaluation in its institutional review board, items related to its institutional review board etc.

(17) Necessary items related to this study

It should be clearly stated that data on symptoms and examinations before informed consent are used in this clinical study.

4.1.3 Revision of Informed Consent Document

If it is determined that revision of the informed consent document is needed, the primary investigator promptly consults with the sponsor or designee and revises informed consent document.

The primary investigator submits the revised informed consent document to the head of medical institution and obtains approval from the institutional review board in advance of its use.

When consent is to be obtained again using the revised informed consent document, the primary investigator or subinvestigator obtains consent from subject in writing in accordance with the procedures defined in "4.1.1 Method for Informed Consent".

4.2 Considerations for Informed Consent

During informed consent, it should be ensured that subject is informed of the facts that despite a follow-up period of 5 years after study procedure, the application for manufacturing and marketing approval in Japan will be filed based on data for 12 months after study procedure and that when the application is approved during follow-up period of 5 years after study procedure, the clinical study will be continued as post-marketing study.

4.3 Target Disease

Ischemic heart disease caused by stenotic, *de novo* lesions developed in coronary arteries

4.4 Inclusion Criteria

4.4.1 General Inclusion Criteria

Patients who meet all of the following criteria are eligible:

(1) Patient must be ≥ 20 years of age.

(2) Patient must have evidence of myocardial ischemia (e.g., stable or unstable angina, silent ischemia, positive functional study or electrocardiogram (ECG) changes consistent with ischemia)

(3) Patients who are able to take dual anti-platelet therapy for 1 year following the index procedure and anticoagulants prior to/during the index procedure.

(4) The subject is an acceptable candidate for Percutaneous Transluminal Coronary Angioplasty (PTCA), stenting, and emergent Coronary Artery Bypass Graft (CABG) surgery.

(5) The subject or subject's legally authorized representative has been informed of the nature of the study and agrees to its provisions and has provided written informed consent as approved by the Institutional Review Board or Ethics Committee of the respective clinical site.

(6) Women of childbearing potential with a negative pregnancy test within 7 days and women who are not pregnant or nursing

(7) Patient must agree to undergo all clinical study required follow up visits, angiograms, and imaging testing

(8) Patient must agree not to participate in any other clinical research study for a period of one year following the index procedure

[Rationale]

(1): This was specified assuming that patient aged 20 years or older is capable of making decision on participation to this clinical study.

(2)-(4): These were specified as conditions to identify subjects for this clinical study.

(5): This was specified to respect and consider free will of subjects and ethics of the clinical study.

(6-8): This was specified for considerations for safety of subjects.

4.4.2 Angiographic Inclusion Criteria

Target lesion vessel must meet the following criteria with confirmation using QCA assessment (online or visual):

(9) Target lesion(s) must be de novo and located in a native coronary artery with a vessel mean diameter of ≥ 2.25 and ≤ 4.0 mm assessed

Target lesion vessel must meet the following criteria with confirmation using visual assessment:

(10) Target lesion(s) must be in a major artery or branch with a visually estimated stenosis of ≥ 50% and < 100% with a TIMI flow of > 1. When two target lesions are treated, they must be located in separate major epicardial vessels

(11) The visually estimated target lesion length is ≤ 34 mm and must be able to be covered by a single 14/15/18/23/28/32/38 mm ELX1805J stent and have at least 2 mm of healthy vessel on either side

Or

(12) The visually estimated target lesion length is ≤ 34 mm and must be able to be covered by a single 15/18/22/30/34/38 mm ZES stent respectively and have at least 2 mm of healthy vessel on either side

(13) The lesion(s) must be successfully pre-dilated prior to enrollment

(14) Mandatory pre-dilatation includes the use of 2 orthogonal views to confirm lesion inclusion and exclusion criteria Successful pre-dilatation of a minimum of 1 Target Lesion, defined as no waist in the inflated pre-dilatation balloon (using two orthogonal views) with a pre-dilatation balloon diameter size approximately 0.25 mm smaller than reference vessel diameter but not more than 0.5 mm smaller than the reference vessel diameter. A residual diameter stenosis prior to study device implantation by visual estimate is recommended to be <30%.Percutaneous intervention of lesions in a non-target vessel if:

- o Not part of a another clinical investigation

- o ≥ 30 days prior to the study index procedure
- o ≥ 6 months after the study index procedure (planned)

(15) Percutaneous intervention of lesions located in the target vessel if:

- o Not part of a clinical investigation
- o ≥ 6 months prior to the study index procedure
- o >12 months after the study index procedure (planned)
- o Previous intervention was distal to and >10 mm from the target lesion

[Rationale]

(9-10): These were specified as conditions to identify subjects for this clinical study. In addition, "2011 ACC/AHA/SCAI Guideline for Percutaneous Coronary Intervention" was referred.
(11-12): These were specified based on sizes of study devices.
(13-15): These were specified for considerations for safety of subjects.

4.4.3 Additional inclusion Criteria for PK study

Patients who meet the following criteria are eligible:

(16) Patients participating in PK study may be treated with only ELX1805J

[Rationale]
(16): This is specified for the purpose of PK study.

4.5 Exclusion Criteria

4.5.1 General Exclusion Criteria

Patients who meet any of the following are ineligible:

(1) The patient was diagnosed with an acute myocardial infarction within the past 72 hours and the CK and CKMBhave not returned to normal (or cTn >15x ULN) and the patient is experiencing clinical symptoms indicative of ongoing ischemia

(2) Patient has a known hypersensitivity or contraindication to aspirin, both heparin and bivalirudin, clopidogrel, prasugrel or ticagrelor, cobalt, nickel, chromium, molybdenum, PLLA polymers or contrast sensitivity that cannot be adequately pre-medicated

(3) Patients with a history of allergic reaction or serious hypersensitivity to drugs exhibiting interactions with sirolimus, zotarolimus, everolimus, tacrolimus, temsirolimus, biolimus and other rapamycin, derivatives or analogues) or similar drugs

(4) Elective surgery is planned within the first 6 months after the procedure that will require discontinuing either aspirin or clopidogrel or other P2Y12 inhibitors.

(5) Patient presenting with chronic (permanent) atrial or ventricular arrhythmia or current unstable ventricular arrhythmias

(6) Patient has a known left ventricular ejection fraction (LVEF) < 30%

(7) Patient has received a heart or other organ transplant or is on a waiting list for any organ transplant

(8) Patient has a malignancy that is not in remission.

(9) Patient is receiving immunosuppression therapy other than steroids and has known immunosuppressive or autoimmune disease (e.g. human immunodeficiency virus, systemic lupus erythematosus etc.)

(10) Patient is receiving chronic anticoagulation therapy (e.g., heparin, coumadin) that cannot be stopped and restarted according to local hospital standard procedures.

(11) Patient has a platelet count < 100,000 cells/mm3 or > 700,000 cells/mm3, a WBC of < 3,000 cells/mm3, or documented or suspected to have cirrhosis of Child-Pugh ≥ Class B within 7 days before study procedure

(12) Patient has known renal insufficiency (e.g., serum creatinine level of more than 2.5 mg/dL within 7 days before study procedure, or patient on dialysis)

(13) Patient has a history of bleeding diathesis or coagulopathy or will refuse blood transfusions

(14) Patient has had a cerebrovascular accident (CVA) or transient ischemic neurological attack (TIA) within the past six months

(15) Patient has had a significant GI or urinary bleed within the past six months

(16) Patient has severe symptomatic heart failure (i.e., NYHA class IV)

(17) Patient has a medical condition that precludes safe 6 French sheath insertion

(18) Patient has other medical illness or known history of substance abuse (alcohol, cocaine, heroin etc.) that may cause non-compliance with the clinical study plan, confound the data interpretation or is associated with a limited life expectancy (i.e., less than one year)

(19) Patient is already participating in another clinical research study which has not reached the primary endpoint (long-term follow-up is not an exclusion)

(20) Other patients whom primary investigator or subinvestigator determined to be ineligible for this clinical study

[Rationale]

(1-20, 22): These were specified for considerations for safety and protection of subjects.
(21): These were specified since non-study treatments affect the results of clinical study.

4.5.2 Angiographic Exclusion Criteria

Patients who meet any of the following are ineligible:

(21) Patients with bypass graft to the target vessel or lesion is located in a bypass graft

(22) Patients with stent implanted within 10 mm of proximal or distal end of target lesion

(23) Patients with a target lesion involving a bifurcation of which the side branch will be jailed by the struts and:
(24) Side branch > 2.5 mm in diameter
(25) Side branch requires planned predilatation (including Kissing Balloon Technique), or Side branch has an ostial lesion or lesion with > 50% stenosis

(26) Patients suspected or confirmed with the QCA analysis of having stenotic lesion of more than 50% in target vessel in addition to target lesion

(27) Patients with target lesion in ostia located within 5 mm of origin of LAD, LCX or RCA

(28) Patients with stenotic lesion in left main trunk

(29) Patients with target lesion that is a chronic total occlusion (CTO) or ≤ TIMI 1 coronary flow in the target vessel

(30) Patients with target vessel that contains thrombus as indicated in pre-procedure angiographic, IVUS or OCT images

(31) Excessive tortuosity ≥ two 45° angles or extreme angulation (≥ 90°) proximal to or within the target lesion–

(32) Patients with target vessel that has moderate to severe calcification that prevents complete angioplasty balloon (POBA with non-compliant balloon, or scoring balloon,) inflation or requires other devices such as rotational atherectomy, rotoblator.

(33) Patients with dissection of Grade A or B that cannot be covered (including 2mm distal to the dissection) with a single study device or with dissection of Grade C or higher

(34) Patients with 2 or more target lesions on 1 branch or target lesions on 3 branches that need to be treated during study procedure

(35) Target lesion involves a myocardial bridge

[Rationale]
(23-29): These were specified since efficacy of PCI has not been verified adequately for this vascular morphology.

(30-35): These were specified for considerations for safety and protection of subjects.

4.5.3 Additional Exclusion Criteria for PK study

Patients who do not meet the following criteria are eligible for the PK study:

(36) Patients with following criteria

a) Patient with PCI within 180 days before study procedure

b) Patient with plan to have staged PCI within 90 days after study procedure

c) Patients who have non-target lesion

[Rationale]
(36): These are specified for clarifying scope of PK study data.

5. Handling of Study Devices

5.1 Name of Study Devices

5.1.1 Multicenter randomized single-blind study

Investigational device: ELX1805J
Control device: Resolute Onyx Coronary Stent System Family (Medtronic Japan Co., Ltd.)

5.1.2 Single-group PK study

Investigational device: ELX1805J

5.2 Shape and Structure of Study Devices

5.2.1 ELX1805J

5.2.1.1 Overview

The ELX1805J SECBS is comprised of the following main components: the platform (called Bioadaptor herein), the PLLA-based polymer topcoat containing the anti-proliferative drug Sirolimus, the PLLA-based polymer basecoat, and the delivery system. The Bioadaptor is crimped onto the balloon of the delivery system. This delivery system is the same delivery system used for the DESyne X2 Novolimus Eluting CSS. Preclinical testing on the ELX1805J SECBS, both *in vivo* and *in vitro*

in addition to *in vivo* and *in vitro* on components and devices with similarities to the ELX1805J SECBS, support both feasibility and safety. Further information regarding the pre-clinical and clinical data can be found in the Investigator's Brochure. A detailed description of the ELX1805J SECBS is provided below.

ELX1805J is fabricated from a CoCr alloy material. The 6 or 8-crown device design incorporates expansion segments within the device pattern designed to disengage after *in vivo* degradation of the bioresorbable polymer coatings. The ELX1805J SECBS is coated with a biodegradable basecoat and topcoat polymers that degrade over 6 - 9 months during which the polymer is converted to carbon dioxide and water and removed during the Krebs Cycle. The basecoat is a biodegradable polylactide-based polymer that is the same polymer that is used for the DESolve scaffold platform. The topcoat is a biodegradable polylactide-based polymer that is the same polymer used in the drug coating of the DESolve scaffold and DESyne BD NECSS. The topcoat incorporates Sirolimus, in a dose of approximately 7 mcg per mm of device length. The total weight of the drug and topcoat polymer and the ratio of the two determine the release profile of the drug. The degradation of the polymer coatings allows the disengagement of the expansion segments, which was demonstrated in *in vivo* evaluations in a porcine animal. After disengagement of the expansion segments, there is a potential to allow the vessel to return to natural vessel motion (i.e. expansion and contraction). This outcome may be similar to that observed with the CE Mark approved DESolve Novolimus Eluting Bioresorbable Coronary Scaffold System.

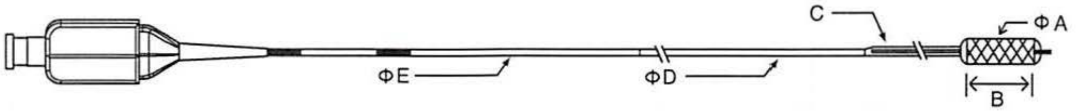

Structural Diagram of ELX1805J (Schematic)

Overview of ELX1805J

Item	Specification
Bioadaptor Diameter [A]	2.25, 2.5, 2.75, 3.0, 3.5, 4.0 mm
Bioadaptor Length [B]	14, 15†, 18, 23, 28, 32, 38 mm
Distal Outer Shaft outer diameter [C]	0.89 mm
Intermediate Shaft outer diameter [D]	0.85 mm
Hypotube outer diameter [E]	0.65 mm
Nominal inflation pressure	10 atm (1,013 kPa)
Maximum inflation pressure	16 atm (1,621 kPa)
Effective length of catheter	140 mm
Minimum inner diameter of guiding catheter	*5 Fr (0.058 inch)

* ≥ 6Fr guide catheter is required for the core lab QCA analysis. A 5Fr guide extension catheter with a 6Fr outer guide catheter is allowed.
†15 mm Bioadaptor Length applies to 4.0 mm diameter only.

5.2.1.2 Design of ELX1805J

Similar to the CE Mark approved DESyne and DESyne BD Novolimus Eluting CSS, the ELX1805J is fabricated from CoCr alloy material. The 6 or 8-crown device design

incorporates expansion segments within the device pattern designed to disengage after *in vivo* degradation of the bioresorbable polymer coatings.

5.2.1.3 ELX1805J Polymer Coatings

The ELX1805J is coated with biodegradable basecoat and topcoat polymers that degrade over 6 - 9 months during which the polymer is converted to carbon dioxide and water and removed during the Krebs Cycle. The basecoat is a biodegradable polylactide-based polymer that is the same polymer that is used for the DESolve scaffold platform. The topcoat is a biodegradable polylactide-based polymer that is the same polymer used in the coating of the DESolve scaffold and DESyne BD NECSS. The topcoat incorporates Sirolimus, in a dose of approximately 7 mcg per mm of device length. The total weight of the drug and topcoat polymer and the ratio of the two determine the release profile of the drug wherein, for the ELX1805J SECBS the majority of the drug Sirolimus (≥ 70%) is released in approximately 4 weeks.

5.2.1.4 Sirolimus

The active pharmaceutical ingredient on the ELX1805J is Sirolimus (also known as Rapamycin). Sirolimus is a macrocyclic lactone produced by Streptomyces hygroscopius. The nominal dosage of Sirolimus on the ELX1805J is approximately 7 mcg per mm of device length. Sirolimus inhibits lymphocyte activation and proliferation that occurs in response to antigenic and cytokine (Interleukin [IL]-2, IL-4, IL-7, and IL-15) stimulation by a mechanism that is distinct from that of other immunosuppressants. Sirolimus also inhibits antibody production. In cells, Sirolimus binds to the immunophilin, FK Binding Protein-12 (FKBP-12), to generate an immunosuppressive complex. Unlike cyclosporine and tacrolimus, the sirolimus: FKBP-12 complex has no effect on calcineurin activity. Rather, this complex binds to and inhibits the activation of a specific cell cycle regulatory protein called the mammalian Target of Rapamycin (mTOR). mTOR is a key regulatory kinase and its inhibition by Sirolimus suppresses cytokine-driven T-cell proliferation, inhibiting the progression from the G1 to the S phase of the cell cycle.

Sirolimus is a well-studied, well-characterized drug approved globally for chronic use in transplant patients. Additionally, Sirolimus is the drug substance incorporated into the CYPHER DES (Cordis Corporation, Miami Lakes, FL), a globally approved device for treatment of patients with ischemic heart disease, marketed between 2002 and 2011.

The drug Sirolimus used on the ELX1805J bioadaptor is being evaluated in this study, has been added to the Elixir family of drug eluting stents/bioadaptors for the following reasons:
1) Sirolimus is widely used in both pharmaceutical and medical devices products;
2) Sirolimus has global acceptance for drug eluting stents from physicians and regulatory authorities; 3) Sirolimus is the base drug for the metabolite Novolimus with which Elixir has extensive clinical experience with excellent results and therefore expects similar results.

5.2.1.5 ELX1805J Delivery System

The ELX1805J Delivery System is identical to the DESyne X2 NECSS in delivery system design and materials. The Delivery System provides a means for carrying the bioadaptor through the coronary vasculature to the desired location and expanding the bioadaptor through inflation of the balloon. The Delivery System is comprised of standard materials with a stainless steel hypotube proximally, a nylon blend shaft distally and nylon blend balloon. Two radiopaque balloon markers mark the working length of the balloon and reflect the expanded bioadaptor length. The radiopaque markers aid in accurately positioning the bioadaptor and delivery system during the implantation and as necessary post-deployment dilation. There are two non-radiopaque markers on the proximal shaft of the delivery system indicating when the distal tip of the catheter exits the tip of a brachial or femoral guiding catheter, respectively. The delivery system is designed to

accommodate a 0.014-inch or smaller diameter guide wire.

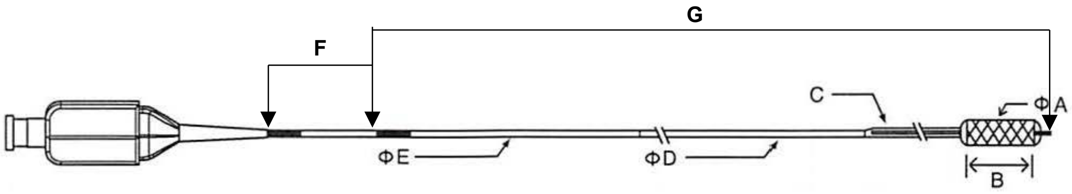

Structural Diagram of ELX1805J (Schematic)

Item	Specification
Bioadaptor Diameter [A]	2.25, 2.5, 2.75, 3.0, 3.5, 4.0 mm
Bioadaptor Length [B]	14, 15†, 18, 23, 28, 32, 38 mm
Distal Outer Shaft Outer Diameter [C]	0.89 mm
Intermediate Shaft Outer Diameter [D]	0.85 mm
Hypotube outer diameter [E]	0.65 mm
Femoral to Brachial Marker Distance [F]	10 cm
Brachial Marker to Distal Tip Distance [G]	90 cm

†15 mm Bioadaptor Length applies to 4.0 mm diameter only.

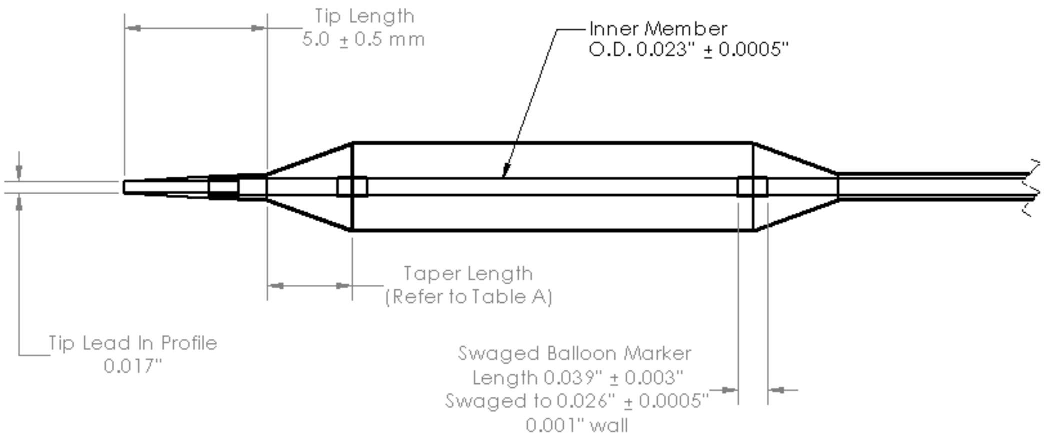

Table A	
**Balloon Diameter**	**Taper Length**
2.25 mm	2.25 ± 0.5 mm
2.5 mm	2.5 ± 0.5 mm
2.75 mm	2.75 ± 0.5 mm
3.0 mm	3.0 ± 0.5 mm
3.5 mm	3.5 ± 0.5 mm
4.0 mm	4.0 ± 0.5 mm

Delivery System Balloon Detail

5.2.1.6 Sizes of ELX1805J used in this study

Length of Device (mm)		14	15	18	23	28	32	38
Diameter of Device (mm)	2.25	X		X	X	X	X	X
	2.5	X	-	X	X	X	X	X
	2.75	X	-	X	X	X	X	X
	3.0	X	-	X	X	X	X	X
	3.5	X	-	X	X	X	X	X
	4.0	-	X	X	X	X	X	X

5.2.1.7 Configurations

ELX1805J has the following primary components.

(1) CoCr Bioadaptor
(2) Rapid exchange (RX) type delivery system
(3) Bioresorbable PLLA-based polymer coatings (basecoat and top coat)
(4) An anti-proliferative drug, Sirolimus (API)

5.2.2 Resolute Onyx Coronary Stent System

5.2.2.1 Overview

Resolute Onyx Coronary Stent System (hereinafter referred to as Resolute Onyx) consists of a cobalt alloy stent that is mounted onto the balloon of the delivery system. The stent is coated with Parylene C, BioLinx and Zotarolimus.

5.2.2.2 Sizes of Resolute Onyx Stents Used in this Clinical Study

Stent length (mm)		15	18	22	30	34	38
Stent diameter (mm)	2.25	X	X	X	X	X	X
	2.5	X	X	X	X	X	X
	2.75	X	X	X	X	X	X
	3.0	X	X	X	X	X	X
	3.5	X	X	X	X	X	X
	4.0	X	X	X	X	X	X

5.2.2.3 Configurations

Resolute Onyx has the following 4 primary components.

(1) Cobalt alloy stent
(2) Rapid exchange (RX) type delivery system
(3) Parylene C and BioLinx polymer coatings
(4) An anti-proliferative drug, Zotarolimus (API)

5.3 Packaging/Labeling of Study Devices

5.3.1 Overview

For investigational devices, those manufactured and packaged at the sponsor in accordance with procedures for manufacturing and quality control of study device are used.

In addition, on the outer package and the outer carton of investigational device, a label listing the following information is affixed.

5.3.2 Information on Outer Package and Outer Carton of Investigational Device

(1) Statement describing: The device is for investigational use.
(2) Name and address of sponsor

(3) Identification of investigation device: ELX1805J
(4) Manufacturing number or manufacturing code
(5) Shelf life
(6) Storage conditions and specifications

5.3.3 Packaging Form of Investigational Device

The packaging form of investigational device is described in Annex 5.

5.3.4 Labeling of Investigational Device

An example of label on investigational device is shown in Annex 6.

5.4 Control of Study Devices

5.4.1 Issuance of Procedures Document for Control of Study Devices

After completion of all study start up activities including the execution of the clinical study contract with the medical institution, the sponsor promptly issues the procedures document describing storage condition, shelf life, usage and handling of study device, and instructions on storage/control and their records (study device control procedures) to the medical institution.

5.4.2 Control of Investigational Device

Study device manager prepares study device control form for receipt, inventory, usage of investigational device for each subject, and return of unused study device to the sponsor or alternative disposal in accordance with the study device control procedures.

5.4.2.1 Storage Conditions

Devices should be stored at ≤ 25°C.

5.4.2.2 Control Method

The study device control procedures are followed.

5.4.2.3 Delivery and Retrieval

Soon after all study start up activities with the medical institution are completed, including the execution of the clinical trial contract, the sponsor delivers study devices to medical institution for the clinical study. Delivery is documented using the form specified by the sponsor.

The sponsor retrieves unused investigational devices from study device manager upon completion of registration of subjects at medical institution. Retrieval is documented using the form specified by the sponsor.

5.4.3 Control of Control Device

5.4.3.1 General Rules on Handling of Control Device

In this clinical study, previously approved medical device (Resolute) is used as control device in a single-blind manner without modification of labeling of medical device, etc. in the medical institution.

Study device manager prepares study device control form for receipt, inventory, and return of unused study device to the sponsor or alternative disposal in accordance with the study device control procedures. The study sites/medical institutions will manage the Resolute Onyx device supplies without sponsor oversight and the device will be used in accordance with the IFU/IB. The sites/medical institutions will maintain records of device usage for the study subjects and will follow institution’s procedures pertaining to commercially approved devices/devices on consignment.

Handling, storage condition, usage, shelf life, and the descriptions on the package insert of medical device concerned and on medical device concerned or its package (including inner package) are followed (Partial Revision of "Guidance on "Ministerial Ordinance on Standards for Clinical Study of Medical Device", PFSB Notification No. 0404-1 issued on April 4, 2013).

5.4.3.2 Delivery of Control Device (transfer of medical device concerned)
As a general rule, medical device is transferred to study device manager at time of use.

5.4.3.3 Preparation of Control Record of Control Device
The study device control procedures are followed per the device instructions for use.

5.5 Labeling of Shelf Life

Shelf life is listed on outer carton. Device should not be used after expiration date of shelf life.

6. Study Method

6.1 Method and Items of Observation

6.1.1 Verification of Eligibility

Primary investigator or subinvestigator verifies that subject consented to participate in clinical study meets both general and angiographic inclusion criteria and does not meet either general or angiographic exclusion criteria.

6.1.2 Medications Prior to the Procedure

DAPT regimen is per current ESC guidelines for PCI (Neumann et al, Eur Hear J 2018).

Stable coronary artery disease: For DAPT naïve patients, a loading dose for clopidogrel and aspirin 6-12 hour prior to the procedure but no less than 2 hours prior to the procedure.
ACS: loading dose of a potent $P2Y_{12}$ inhibitor (clopidogrel, ticlopidine, prasugrel, or ticagrelor) and aspirin.

6.1.3 Assessment Prior to the Study Procedure

Observation/test items to be assessed at medical institution before study procedure are listed below.

Informed consent	Date of informed consent
Date of hospitalization	Date of hospitalization
Verification of eligibility	General/angiographic inclusion criteria, general/angiographic exclusion criteria
Demographic information	Date of birth, gender
History of cardiac diseases	Status and timing of PCI, status and timing of treatment of MI<br>Status and timing of CABG, presence/absence of CHF
Past history	Presence/absence and timing of onset of cerebral infarction
Risk factors for ischemic heart diseases	Smoking status, presence/absence of diabetes, treatment (none, pharmacotherapy, insulin therapy, exercise therapy), presence/absence of dyslipidemia, presence/absence of hypertension, presence/absence of family history of coronary artery disease

Complications (other than risk factors for ischemic heart diseases)	Presence/absence of complication, name of disease
Assessment of angina pectoris	CCS classification, Braunwald classification, silent myocardial ischemia (method of verification)
12-lead ECG	To be conducted within 7 days before study procedure, date of procedure
Clinical laboratory test	Blood sampling within 7 days before study procedure [WBC, Plt, Cre], women of childbearing potential [pregnancy test, urine or blood], date of blood sampling, measured value.
Cardiac enzymes	Blood sampling within 72 hours before study procedure (measurement within 24 hours if AMI is suspected) [CK, CK-MB or troponin], date and time of blood sampling, Presence/absence and classification of acute myocardial infarction
LVEF	To be evaluated by the most recent imaging exam (i.e., echocardiogram, ventriculogram, MUGA, etc.) within 30 days before study procedure, measured date, measured value
Concomitant medications	Use of aspirin and thienopyridine-based antiplatelets, dose, anticoagulants, drugs for cardiac disease<br>DAPT regimen is per current ESC guidelines for PCI (Neumann et al, Eur Hear J 2018).<br>Stable coronary artery disease: For DAPT naïve patients, a loading dose for clopidogrel and aspirin 6-12 hour prior to the procedure but no less than 2 hours prior to the procedure.<br>ACS: loading dose of a potent $P2Y_{12}$ inhibitor (Clopidogrel, ticlopidine, prasugrel, or ticagrelor) and aspirin.

6.1.3.1 Confirmation of Location of Target Lesion

As for confirmation of location of target lesion and coronary angiography (CAG), see section 7.2.

6.1.3.2 Verification of Angiographic Criteria, Enrollment and Allocation of Cases

Primary investigator or subinvestigator verifies that subject meets inclusion criteria for angiography and does not meet exclusion criteria for angiography. After successful pre-dilation of target lesion, randomization is conducted via the study EDC database. If there is a non-target lesion, allocation is conducted after achievement of lesion success for the non-target lesion and successful pre-dilation of target lesion. When allocation is conducted, the subject is considered as one case regardless of the number of lesions treated.

6.1.4 During Study Procedure

Observation/test items to be assessed during study procedure are listed below.

Study device	ELX1805J (investigational device)<br>Resolute Onyx (control device)
Date of study procedure	Date of study procedure, start time and end time of study procedure, person who performed study procedure
Number of lesions	Number of lesions
Lesion number	AHA number

Percutaneous access	By femoral approach, upper arm approach or radial artery approach
Treatment of non-target lesion	Presence/absence of non-target lesion, AHA number<br>Name/size of treatment device<br>Lesion success/failure
Treatment of target lesion<br>(before implantation of study device)	AHA number, reference blood vessel diameter, rate of stenosis, lesion length<br>Size of guiding catheter<br>Presence/absence of pre-dilation, name/size/maximum inflation pressure of device<br>Residual stenosis after pre-dilation, TIMI flow classification, presence of dissection, angiographic complications, chest pain
Treatment of target lesion<br>(implantation of study device)	Number of study devices used<br>Name/manufacturing number/size/maximum inflation pressure/expansion time of study device<br>(In cases of implantation of multiple devices) Presence/absence of overlap
Treatment of target lesion<br>(after implantation of study device)	Presence/absence of post-dilation, name/size/maximum inflation pressure of device<br>Residual stenosis, TIMI flow classification<br>[Other treatment conducted for target lesion]<br>Lesion, Device, and Procedure success/failure
Perioperative complications	Events that occurred from insertion of study device into guiding catheter to completion of study procedure
Operational failure, etc.	Presence/absence of malfunctions of study device and details if any
Adverse events	Events which meet protocol definition and serious adverse events that occurred from insertion of study device into guiding catheter to completion of study procedure
Concomitant medications	Presence/absence of concomitant use of drugs during procedure, type and dose of drugs used<br>Presence/absence of use of heparin and dose, coronary injection of coronary vasodilator (nitro, ISDN), type and dose of contrast agent

6.1.4.1 Implantation Procedure of Study Devices

As for implantation procedure of study device, see sections 7.4 to 7.5.

6.1.5 After Study Procedure to Discharge

Observation/test items to be assessed after completion of study procedure and by discharge.

Assessment of angina pectoris	CCS classification, Braunwald classification, silent myocardial ischemia (method of verification)
12-lead ECG	Assessments are conducted within 12 to 24 hours after study procedure. If a subject is discharged before 12 hours, assessment is conducted before discharge.

Cardiac enzymes	Blood is collected within 12 to 24 hours after study procedure [CK, CK-MB or Troponin]. If a subject is discharged before 12 hours, blood is collected prior to discharge.<br><br>＊When CK or CK-MB exceeds upper limits of institutional reference value, measurements are conducted three times at an interval of 8 hours until cardiac enzyme shows clear decrease or returns to institutional reference value, or measurements are conducted according to each institutional rule (at doctors' discretion).
Adverse events	Events which meet protocol definition and serious adverse events that occurred after study procedure and by discharge
Concomitant medications	Type of antiplatelet, anticoagulant and other drugs used concomitantly for cardiac diseases

6.1.6 Observation Items for Follow-Up

6.1.6.1 Follow-Up at 1 Month after Study Procedure (Day 30 ± 7 days)

Observation/test items to be assessed at medical institution at 1 month after study procedure are listed below. (contact must be conducted to patients within the specified allowance period as possible.)

Hospital visit	Status of hospital visit by subject, date of visit, reason if visit was not made by subject
Assessment of angina pectoris	CCS classification, Braunwald classification, silent myocardial ischemia (method of verification)
Adverse events	Events which meet protocol definition and serious adverse events that occurred after discharge and by follow-up visit at 1 month after study procedure
Concomitant medications	Type of antiplatelet, anticoagulant and other drugs used concomitantly for cardiac diseases

6.1.6.2 Follow-Up at 6 Months after Study Procedure (Day 180 ± 30 days)

Observations/tests to be conducted at hospital visit or via telephone at 6 months after study procedures are listed below. (contact must be conducted to patients within the specified allowance period as possible.)

Hospital visit or Telephone contact	Status of hospital visit/telephone contact by subject, date of follow-up, reason if visit was not made by subject
Assessment of angina pectoris	CCS classification, Braunwald classification, silent myocardial ischemia (method of verification)
Adverse events	Events which meet protocol definition and serious adverse events that occurred after follow-up visit at 1 month after study procedure and by follow-up visit at 6 months after study procedure
Concomitant medications	Type of antiplatelet, anticoagulant and other drugs used concomitantly for cardiac diseases

6.1.6.3 Follow-Up at 12 Months after Study Procedure (Day 365 ± 30 days)

Observation/test items to be assessed at medical institution at 12 months after study procedure are listed below. (Its contact must be conducted to patients within the specified allowance period as possible.)

Hospital visit	Status of hospital visit by subject, date of visit, reason if visit was not made by subject
Assessment of angina pectoris	CCS classification, Braunwald classification, silent myocardial ischemia (method of verification)
Imaging Subset	Complete clinical assessment evaluation and input result into CRF before conducting imaging assessment
Adverse events	Events which meet protocol definition and serious adverse events that occurred after follow-up visit at 6 months after study procedure and by follow-up visit at 12 months after study procedure
Concomitant medications	Type of antiplatelet, anticoagulant and other drugs used concomitantly for cardiac diseases

6.1.6.4 Follow-Up at 2 to 5 Years (± 30 days) after Study Procedure

Observations/tests to be conducted at hospital visit or via telephone every year after 12 months from study procedures (year 2 to 5) are described below. (Its contact must be conducted to patients within the specified allowance period as possible.)

Hospital visit	Status of hospital visit/telephone contact visit by subject, date of visit, reason if visit was not made by subject
Assessment of angina pectoris	CCS classification, Braunwald classification, silent myocardial ischemia (method of verification)
Adverse events	Following events which meet protocol definition and all serious adverse events that occurred since previous follow-up<br>• Device/stent thrombosis rate<br>• Adverse events, serious adverse events, malfunctions<br>• All cardiovascular events regardless of seriousness or device relationship<br>• All study device-related events and events for which the relationship to the study device is unknown<br>• All unanticipated adverse device effects<br>• All CVAs (cerebrovascular accident)
Concomitant medications	Antiplatelet drugs

6.1.6.5 Records of Completion/Discontinuation of Study

Completion of 5 years of FU	Status of completion of 5 years of FU
In cases of discontinuation, reason	Lost to follow-up, death, withdrawal of consent, others, etc.

6.1.7 Measurement of plasma drug concentration for PK Study

6.1.7.1 Measurement period

Blood draws are conducted at below measurement period

- Before study procedure
- 10 (± 2 min) and 30 (± 4 min) minutes after study procedure
- 1 (± 8 min), 2 (± 15 min), 4 (± 30 min), 6 (± 30 min), and 12 (± 30 min) hours after study procedure
- 24 (± 2 hour) and 72 (± 6 hour) hours after study procedure
- 7 (± 24 hour) days after study procedure

6.1.7.2 Measurement method and storage method of PK sample

It should be followed by procedure separately.

6.1.8 Information on Adverse Events Related to Endpoints

In cases of death, myocardial infarction, CI-TLR (CABG, PCI), CI-TVR (CABG, PCI), bleeding complications and vascular complications, name of event, occurrence date, severity, seriousness, treatment, outcome and relationship etc. in every event are entered into applicable pages of e-CRF.

In addition, since adverse events mentioned above are evaluated by the clinical event committee using source documents*, medical institution masks information that identifies subject and provides the sponsor with a copy of document.

* Source Document: Summary at discharge, ECG data, laboratory test data, catheter record/PCI record, angiographic data (CD-ROM), death certificate/autopsy certificate etc.

Subjects who have anginal symptoms will undergo proper clinical or angiographic follow-up any time it is clinically indicated. ECGs shall be recorded for any suspected ischemic episode. Clinically-indicated angiography should be preceded by angina pectoris assessment (e.g. worsening of angina status, ECG changes, a positive stress test or evidence of silent ischemia etc.). Primary investigator or subinvestigator should attempt to indicate whether or not the subject's clinical status would warrant revascularization in advance of observing angiography.

Repeat Revascularization

Physicians should measure and report fractional flow reserve measurement or instant wave free ratio measurement **prior** to performing a repeat revascularization. QCA including two orthogonal views of the target lesion and target vessel are required prior to revascularization of a lesion .

The Cines should be stored and retrievable for CEA, monitoring, and Core lab analysis.

The results of the Angiographic Core Laboratory will be used for data analysis for lesion identification and characteristics.

6.2 Flowchart of Clinical Study

Procedures from obtainment of informed consent to completion of assessment of study device are shown in diagram below.

Screening

- Screening of subject
  Considering inclusion and exclusion criteria, etc., potential subjects are selected as candidates of study.

↓

Informed Consent
Verification of eligibility
Pre-procedure observation

- Explanation of clinical study, informed consent
- Verification of Eligibility
  Verification for general inclusion criteria and general exclusion criteria
- Verification of concomitant medications (antiplatelet therapy)
  Aspirin: maintenance dose is administered before study procedure in accordance with package insert
  Clopidogrel or Prasugrel： in accordance with package insert, maintenance dose is administered before study procedure, or loading dose is administered before initiation of study procedure (If ticlopidine is in use, it is allowed to continue to take ticlopidine at physician's discretion.)
- Pre-procedure observation
  Assessment of angina pectoris
  Clinical laboratory test*1
  CK, CK-MB or troponin*2, 12-lead ECG*1, LVEF*3

*1: Within 7 days before study procedure
WBC, Plt, Cre, pregnancy test for women of childbearing potential
*2: Within 72 hours before study procedure
(within 24 hours if there is any sign of AMI)
*3: Within 30 days before study procedure

↓

During study procedure

- Verification by on-line QCA before implantation of device (assessment of lesion)
- Verification of eligibility for angiographic selection/exclusion criteria (confirmation of lesion success if there is non-target lesion)
- Successful pre-dilation of site of target lesion → **Randomization via EDC database**
- Implantation of study device
- Verification by QCA after implantation of study device
- Post dilatation at operator's discretion as is the use of intra-vascular imaging techniques
- IVUS imaging or IVUS + OCT performed for subset subjects
- Observation for concomitant medications, perioperative complications and adverse events

↓

After Study Procedure to Discharge
(Note that blood test is conducted at 10, and 30 minutes, 1, 2, 4, 6, 12, 24, and 72 hours, 7 days after study procedure if it is PK study

Assessment of angina pectoris
12-lead ECG*4
CK, CK-MB (or troponin)*5
Verification of acute success
Observation for concomitant medications and adverse events

*4: Conducted within 12 to 24 hours after study procedure. If a patient is discharged before 12 hours after procedure, measurement shall be conducted before its discharge.
*5: Within 12 to 24 hours after study procedure (In cases of a value higher than upper limit of institutional reference value, measurements are made 3 times at 8 hours interval or measurements are conducted according to each institutional rule (at doctors' discretion))

↓

1 month after study procedure
(hospital visit or TEL)

Assessment of angina pectoris
Observation for concomitant medications and adverse events

↓

**6 months after study procedure (hospital visit or TEL)**	Assessment of angina pectoris<br>Observation for concomitant medications and adverse events	
**12 months after study procedure (*hospital visit – if the subject is not in the imaging subset, a remote visit is acceptable)**	Assessment of angina pectoris<br>Subset imaging conducted using QCA and IVUS or QCA + IVUS + OCT<br>Observation for concomitant medications and adverse events	6: The investigator must document the results of the clinical assessment in the CRF before proceeding to the imaging follow up assessment
**Subsequently, every year until 5 years after study procedure (hospital visit or TEL)**	Assessment of angina pectoris<br>Observation for concomitant medications and adverse events*<br>*Only cardiovascular events (regardless of seriousness or device relationship), study device-related events and events for which the relationship to the study device is unknown, unanticipated adverse device effects and CVAs (cerebrovascular accident) after the one year visit.	

7. Usage of Study Devices

7.1 Preparation of Study Procedures

Before use of study device, sterile package of study device should be checked carefully that it is not damaged or opened, and shelf life should be checked before opening package. Study device should be removed carefully from dispenser and checked carefully for bending, distortion or any other damage. Device should not be used if there is any damage. Guide wire with an outside diameter of 0.014 inch or smaller and guiding catheter of 6 Fr or larger should be used

7.2 Coronary Angiography and Confirmation of Location of Target Lesion

Primary investigator or subinvestigator conducts coronary angiography before implantation of study device and determines size and implantation location of study device for proper treatment of target lesion. To determine eligibility of subject, the QCA analysis is performed for diameter of reference vessel before randomization. In addition, when it is allocated for investigational device, Mean reference vessel diameter (RVD) and Dmax is measured by QCA analysis after pre-dilation. After confirming success of pre-dilation, based on mean RVD (QCA analysis) and length of lesion (visual observation), size of study device is determined. As for method for CAG, the guidelines on angiography prescribed separately are followed.

In addition, CAG data collected at medical institutions are analyzed centrally at the core laboratory, and transfer of CAG data between medical institutions and the core laboratory is conducted in accordance with procedures defined separately.

Core laboratory reviews and confirms if the subject information meet the inclusion and exclusion criteria for angiography defined in the protocol. The operators who do not comply with the criteria should be informed and requested to take preventive actions so that they can comply with the procedures for angiography at follow-up observation and clinical evaluation.

7.3 Treatment of Non-Target Lesion during Study Procedure

If non-target lesion is present on non-target vessel, it is treated before treatment of target lesion with previously approved devices other than the study device, and it is verified that non-target lesion is treated successfully without any perioperative complication.

As for successful treatment, refer to definitions below. When treating non-target lesion with a DES, the DES shall have an ‘olimus family drug coating. No other restriction is specified for non-target lesion.

[Definition of successful treatment of non-target lesion]

- final diameter stenosis ≤ 10% with final TIMI-3 flow
- no residual dissection
- no transient or sustained angiographic complications (e.g., distal embolization, side branch closure)
- no chest pain lasting ≥ 5 minutes
- no ST segment elevation or depression lasting ≥ 5 minutes

7.4 Treatment of Target Lesion

7.4.1 Pre-Dilatation of Target Lesion for Treatment

Pre-dilatation

Pre-dilatation is mandatory including the use of 2 orthogonal views to confirm lesion inclusion and exclusion criteria. Successful pre-dilatation of a minimum of 1 Target Lesion, defined as no waist in the inflated pre-dilatation balloon (using two orthogonal views) with a pre-dilatation balloon diameter size approximately 0.25 mm smaller than reference vessel diameter but not more than 0.5 mm smaller than the reference vessel diameter. A residual diameter stenosis prior to study device implantation by visual estimate is recommended to be <30%. It is strongly recommended to use a slower inflation technique (1 atm per second) and at the final pressure, hold pressure for at least 30 seconds unless prevented by chest pain/ECG changes

- Successful pre-dilatation of the lesion is mandatory prior to the patient receiving the study device
- Dissection
  - Patients with Grade A or B dissections after pre-dilatation that can be covered by a single study device may be treated per the implantation procedure described in Procedure for Implantation below.
  - Lesions with pre-dilatation dissections Grade C or higher or Grade A or B dissections requiring treatment that cannot be covered by a single study device (ELX 1805J or control device) are excluded from the study.

7.4.2 Procedure for Implantation of ELX1805J Device in Target Lesion

Implantation of assigned device

In order to eliminate or mitigate peri-stent effects, special care needs to be taken to minimize injury to areas adjacent to the stent boundaries. Therefore the following guidelines need to be followed:

- Following successful predilatation administer 100 – 200 mcg of intracoronary nitroglycerin, or isosorbide dinitrate (dose is per the administering physician)to accurately assess vessel size and evidence of dissections
- Using either visual assessment or on-line QCA, select the appropriate size assigned device diameter (QCA) and length (visual). If the correct size is not available or the patient does not meet all clinical and angiographic inclusion criteria including a vessel taper of 0.5 mm or greater proximal to distal in the segment to be treated, the patient should not be included in the study
- Select a device diameter equal to or greater than the mean reference vessel diameter (MRVD) or no less than 0.25 mm smaller than Dmax. Dmax should not be larger than 4.25 mm. The stent length should cover the lesion and at least 2mm of healthy vessel on either side (healthy to healthy). For appropriate size of the ELX1805J device for lesion length, see table below.
- The assigned device is inserted into blood vessel and guided along the guide wire to the site of implantation. Once the system has been inserted into the guiding catheter, timing of the procedure should be started.
- Expand assigned device to at least nominal pressure and up to RBP (not to exceed RBP) to ensure good apposition throughout the stented segment. It is recommended to use a slower inflation technique (1 atm per second) and at the final pressure, hold pressure for at least 30 seconds unless prevented by chest pain/ECG changes.

Lesion length	ELX1805J size		
≤ 10 mm	2.25	x	14 mm
	2.5	x	14 mm
	2.75	x	14 mm
	3.0	x	14 mm
	3.5	x	14 mm
	4.0	x	15 mm
≤ 14 mm	2.25	x	18 mm
	2.5	x	18 mm
	2.75	x	18 mm
	3.0	x	18 mm
	3.5	x	18 mm
	4.0	x	18 mm
≤ 19 mm	2.25	x	23 mm
	2.5	x	23 mm
	2.75	x	23 mm
	3.0	x	23 mm
	3.5	x	23 mm
	4.0	x	23 mm
≤ 24 mm	2.25	x	28 mm
	2.5	x	28 mm
	2.75	x	28 mm
	3.0	x	28 mm
	3.5	x	28 mm
	4.0	x	28 mm
≤ 28 mm	2.25	x	32 mm
	2.5	x	32 mm
	2.75	x	32 mm
	3.0	x	32 mm
	3.5	x	32 mm
	4.0	x	32 mm
≤ 34mm	2.25	x	38 mm
	2.5	x	38 mm
	2.75	x	38 mm
	3.0	x	38 mm
	3.5	x	38 mm
	4.0	x	38 mm

- Post-dilatation at operator's discretion as is the use of intra-vascular imaging techniques for patients not included in the IVUS or IVUS + OCT subset. If post dilatation is performed, post-dilate with a non-compliant balloon and hold pressure for at least 30 seconds unless prevented by chest pain/ECG changes to ensure adequate stent apposition in all stent segments.
- Goal is 10% or less and no more than 15% residual DS
- Do not expand the Bioadaptor > 0.5 mm above the nominal labeled diameter per the IFU.
- Dissection
    - Bailout treatment may be conducted using up to one additional study device from the assigned treatment group sufficient to cover the affected area utilizing the shortest available study device. The devices must

overlap by at least 1 - 2 mm. If additional stents or other sizes are needed, any non-study, approved DES stent incorporating an "olimus" drug and cobalt chromium metal or bare metal stent should be used.

7.4.3 Procedure for Implantation of Control Device in Target Lesion

Implantation of control device

- Control device is implanted in accordance with the Instructions for Use (IFU) of control device.
- The pre-dilatation balloon should be sized to approximately 0.5mm less than the MRVD and slowly and gradually inflated to 0.25 mm less than MRVD. Ensure full balloon expansion in two orthogonal views (no waist observed in both orthogonal views and lumen diameter no less than 0.5 mm smaller than the vessel diameter).
- Following successful pre-dilatation, intracoronary nitroglycerin or isosorbide dinitrate should be administered (as appropriate for patient safety) to ensure vessel diameters meet inclusion/exclusion criteria and for device selection and sizing.
- To perform the QCA measurements accurately, guiding catheter of 6 Fr or larger should be used. When any resistance is felt at any time between lesion access and removal of delivery system, delivery system and guiding catheter should be removed together.
- For coverage with a single investigational device, the length of target lesion in QCA assessment should be 34 mm or shorter, and a single device should cover at least additional 2 mm at both proximal and distal ends of lesion extending from healthy vessel to healthy vessel. For appropriate size of control device for lesion length, see table below.
- For RBP and the stent maximum expansion pressure, IFU should be followed.

Length of lesion (visual estimation)	**Resolute Onyx**
≤ 10 mm	2.25 x 15 mm<br>2.5 x 15 mm<br>2.75 x 15 mm<br>3.0 x 15 mm<br>3.5 x 15 mm<br>4.0 x 15 mm
≤ 14 mm	2.25 x 18 mm<br>2.5 x 18 mm<br>2.75 x 18 mm<br>3.0 x 18 mm<br>3.5 x 18 mm<br>4.0 x 18 mm
≤ 18 mm	2.25 x 22 mm<br>2.5 x 22 mm<br>2.75 x 22 mm<br>3.0 x 22 mm<br>3.5 x 22 mm<br>4.0 x 22 mm
≤ 24 mm	2.25 x 30 mm<br>2.5 x 30 mm<br>2.75 x 30 mm<br>3.0 x 30 mm<br>3.5 x 30 mm<br>4.0 x 30 mm

Length of lesion (visual estimation)	**Resolute Onyx**
≤ 28mm	2.25 x 34 mm<br>2.5 x 34 mm<br>2.75 x 34 mm<br>3.0 x 34 mm<br>3.5 x 34 mm<br>4.0 X 34 mm
≤ 34mm	2.25 x 38 mm<br>2.5 x 38 mm<br>2.75 x 38 mm<br>3.0 x 38 mm<br>3.5 x 38 mm<br>4.0 x 38 mm

- Post-dilate according to the manufacturer IFU.
- Goal is 10% or less and no more than 15% residual DS.
- Dissection
    - Bailout treatment may be conducted using up to one additional study device from the assigned treatment group sufficient to cover the affected area utilizing the shortest available study device. The devices must overlap by at least 1 - 2 mm. If additional stents or other sizes are needed, any non-study, approved DES stent incorporating an "olimus" drug and cobalt chromium metal or bare metal stent should be used.

7.4.4 Intervention for Multiple Target Lesions

During study procedure, up to 2 lesions in different epicardial vessels may be treated with the assigned study device. When there are multiple TLF, the primary endpoint, in a single case, it is counted per case (counted as single incidence).

Where there are two de novo lesions of size that can be covered with one study device on different blood vessels, they are treated as described below.

(1) Culprit lesion (lesion with higher % diameter stenosis) is selected as the target lesion to be treated first and called the first target lesion. After success of pre-dilation is verified, subject is randomized, and the assigned study device is implanted. If successfully treated, treatment of the second target lesion can be initiated. If pre-dilation is not successful, the lesion is treated as a non-target lesion and the second lesion is now treated as the target lesion (the patient is counted as having a single lesion).

[Definition of successful treatment of the target lesion]

- o Residual stenosis of 15% or less in visual observation
- TIMI-3 flow (visual assessment by operator)
- no dissection of higher than Grade C and able to be covered by a single assigned device
- no angiographic complication (e.g., distal embolization)

- no chest pain lasting 5 minutes or longer
- no elevation or decline in the ST-segment lasting for 5 minutes or longer.

- When lesion success was not achieved for the first target lesion (treatment failure), the primary investigator or subinvestigator should perform all necessary treatment to achieve the best angiographic results or clinical results. This treatment includes additional balloon dilation, implantation of stent (allocated study device is recommended) and administration of thrombolytic drug.

As described above, when implantation of additional stent or other treatment was necessary and/or adequate treatment results were not achieved for the first target lesion, the second target lesion may be treated with previously approved devices at discretion of primary investigator or subinvestigator. However, this lesion is no longer considered to be target lesion.

(2) Treatment of the second target lesion with allocated study device is initiated after achievement of lesion success for the first target lesion without any additional treatment.

7.4.5 IVUS Imaging Subset

- In the IVUS subset, IVUS should be performed after optimal stent placement has been obtained (physician's assessment based on angiographic results). If IVUS indicates that stent placement is not optimal and additional post-dilatation is performed, IVUS imaging should be repeated after optimal stent placement has been obtained based on angiography. The final images will be used for data analysis. Angiography and IVUS should be performed following the core laboratory protocols. Angiography and IVUS will be conducted again at 12-month follow-up in the subset.
- Target in-stent cross sectional lumen area by IVUS should be no less than as recommended by the AVIO Trial (Chieffo, et al)[1]:

Device Diameter	Target MLA
• **2.5 mm**	• **No less than 4.0 mm^2**
• **3.0 mm**	• **No less than 6.0 mm^2**
• **3.5 mm**	• **No less than 8.0 mm^2**
• **4.0 mm**	• **No less than 10 mm^2**

- Following IVUS, post-dilate again as necessary (if needed) with a non-compliant balloon and ensure that no clinically significant stent malapposition is present or uncovered dissection.

7.4.6 IVUS + OCT Imaging Subset

- In the IVUS + OCT subset, IVUS + OCT should be performed after optimal stent placement has been obtained (physician's assessment based on angiographic results). If IVUS or OCT indicates that stent placement is not optimal and additional post-dilatation is performed, OCT imaging should be repeated after optimal stent placement has been obtained based on angiography. The final images will be used for data analysis. Angiography and IVUS+ OCT should be performed following the core laboratory protocols. Angiography and IVUS + OCT will be conducted again at 12-month follow-up in the subset.

7.4.7 Flow Chart Intervention

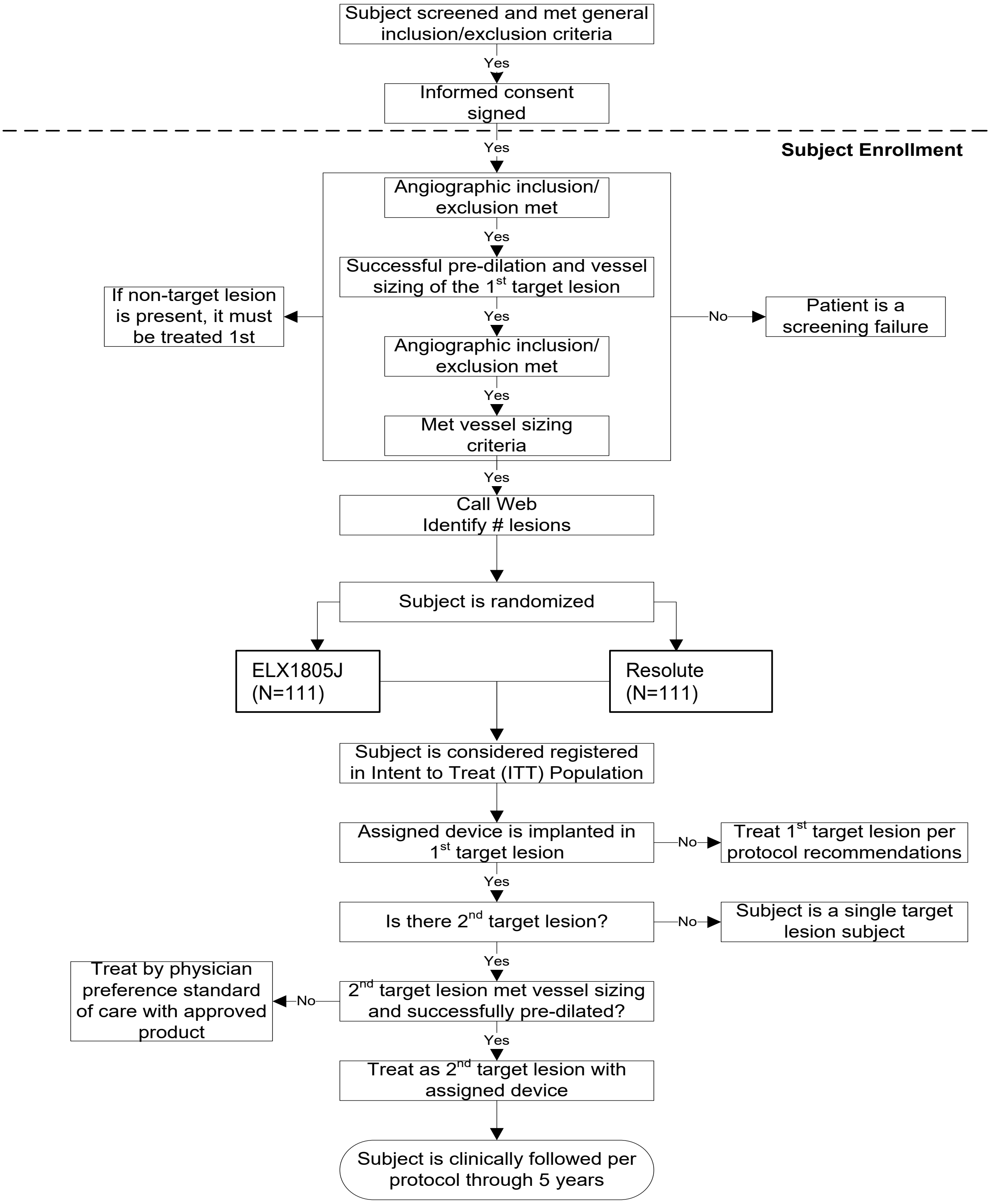

Single Vessel Treatment

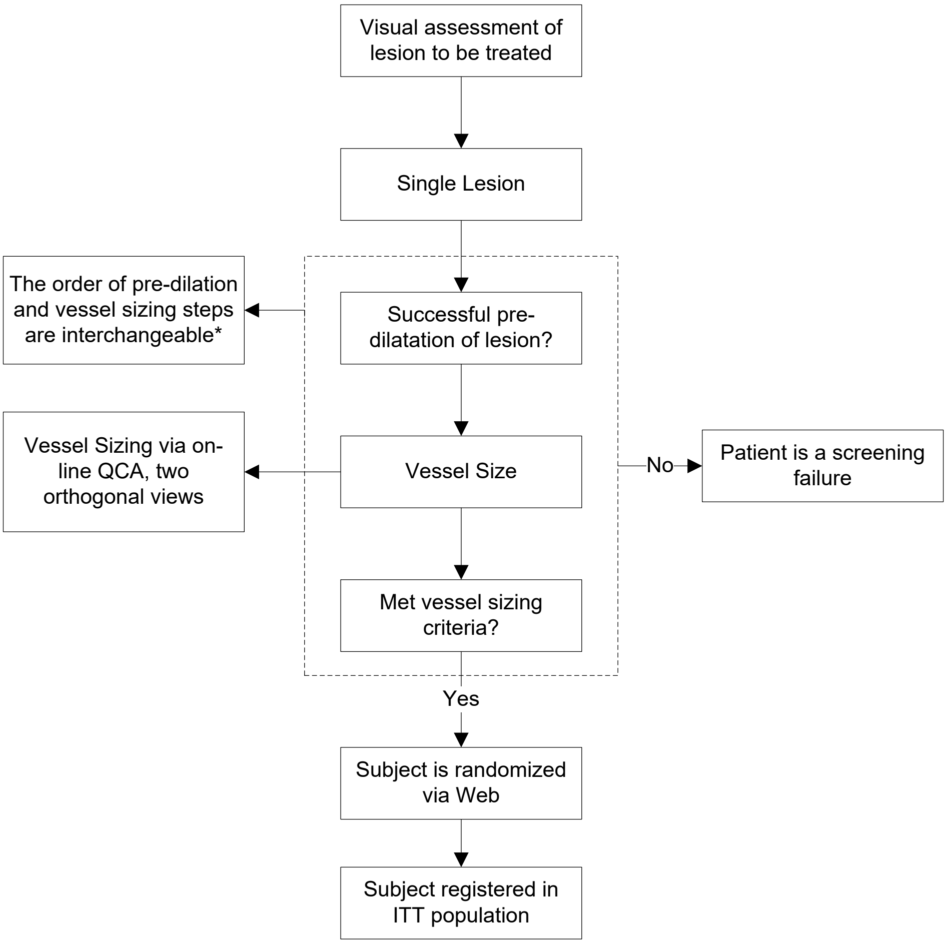

* The lesion must be successfully pre-dilated <u>and</u> meet vessel sizing criteria prior to randomizing the subject.

Two Vessel Treatment

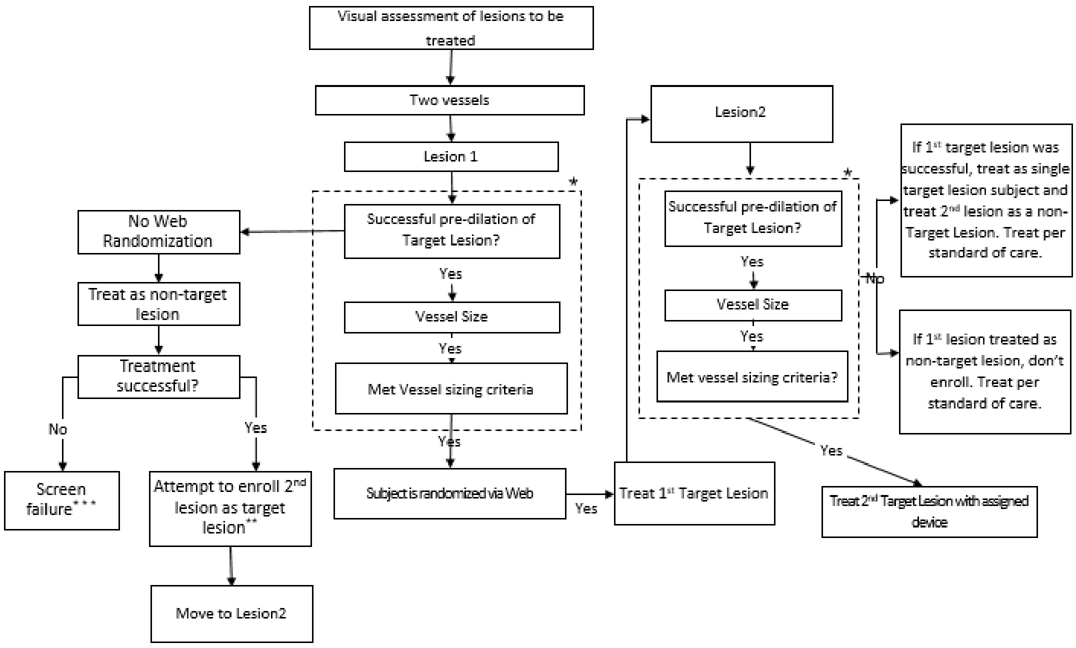

*The order of pre-dilation and vessel sizing steps are interchangeable
**If 1st lesion is a non-target lesion, follow single vessel treatment flow.
***If treatment of non-target lesion is unsuccessful, the patient is a screen failure.

7.5 Bailout Procedure Immediately after Implantation

Procedures for Bailout and Incomplete Coverage
If any of the followings is observed in patient, bailout procedure may be performed.

- Vascular dissection which requires treatment
- Apparent occlusive complication with blood flow decrease in target vessel
- Chest pain, changes in ECG with ischemia that do not improve with balloon-dilation, medical therapy or thrombolytic agent cannot recover
- Case which requires unscheduled additional stent to cover target lesion
- Case which causes possible clinical complication or confirms unsolvable device malapposition
- Any finding which proves device damage

To cover the entire study device as bailout procedure, previously approved DES with an “olimus" family of drugs is used, and for coverage of edge of study device, an allocated single study device with the shortest length that covers lesion sufficiently can be used. In addition, devices should overlap for 1 to 2 mm. If a suitable size variation of the study device is not available or if additional stents are required, previously approved DES with an “olimus" family of drugs can be used.

When treating a dissection, the device should be able to cover 2 mm distal to the dissection. After treatment, coronary angiography should be conducted to confirm appropriate coverage. Use of different types of stents in the same blood vessel should be avoided unless it is necessary for safety of subject. In this procedure, crossover to the other study device is not allowed.

7.6 Completion of Study Procedure and Management after Study Procedure

Additional heparin should be administered to subject as necessary after study procedure is completed. Treatment after study procedure (ACT monitoring, removal of sheath) and

management of subjects after study procedure are conducted in accordance with procedures at each medical institution. Previously approved hemostatic device may be used at discretion of primary investigator or subinvestigator.

MRI scans should not be performed on patient’s post-device/stent implantation until the device/stent has been completely endothelialized, > 8 weeks.

8. Drugs, Therapies and Devices Used Concomitantly

8.1 Drugs, Therapies and Devices Used Concomitantly

8.1.1 Concomitant Drugs

Before study procedure	DAPT as per current ESC guidelines for PCI (Neumann et al, Eur Hear J 2018).<br>Stable coronary artery disease: For DAPT naïve patients, a loading dose for clopidogrel and aspirin 6-12 hour prior to the procedure but no less than 2 hours prior to the procedure.<br>ACS: loading dose of a potent $P2Y_{12}$ inhibitor (clopidogrel, ticlopidine, prasugrel, or ticagrelor) and aspirin.	
During study procedure	Heparin	Standard administration method at medical institution is followed.
	nitroglycerin or isosorbide dinitrate	To be administered prior to vessel sizing; standard administration method at medical institution is followed.
After study procedure	DAPT therapy should be continued for a minimum of 6 months for stable patients and 12 months for ACS patients unless there are contraindications.	

Remarks:
- In cases of adverse drug reaction, antiplatelet may be switched at physician's discretion. In addition, depending on health status of subject, dose adjustment or temporary suspension is allowed. Primary investigator and subinvestigator refer to the attached document of concomitant drugs about an expected adverse reaction.
- In cases of use of 2 or more study devices for a single lesion, dissection, poor distal blood flow, unacceptable residual stenosis or thrombus, antiplatelet or anticoagulant may be added at discretion of physician.

8.1.2 Drugs that May not be Used Concomitantly

While no specific clinical data are available, drugs like tacrolimus which act through the same binding protein (FKBP) may interfere with the efficacy of Sirolimus or Rapamycin-type drugs. Drug interaction studies have not been performed. Rapamycin-type drugs are metabolized by CYP3A4. Strong inhibitors of CYP3A4 (e.g. ketoconazole) might cause increased Sirolimus exposures to levels associated with systemic effects, especially if multiple bioadaptors are deployed. Systemic exposure of Sirolimus or Rapamycin-type drugs should also be taken into consideration if the patient is treated concomitantly with systemic immunosuppressive therapy. Grapefruit may potentially interfere with the metabolism of Sirolimus.

8.1.3 Drugs that Should be Administered Carefully in Concomitant Use

Those drugs listed on the instruction manual of investigational device and the package inserts of control device (Resolute Onyx) should be administered carefully when they are used concomitantly.

8.1.4 Devices and Therapies that May not be Used Concomitantly

Devices (rotablator, etc.) used for procedures other than those allowed for target lesion (e.g., semi-compliant or non-compliant balloon/scoring balloon/cutting balloon).

8.1.5 Drugs that May be Used Concomitantly

Drugs that primary investigator or subinvestigator determined to be necessary for treatment of underlying disease or complication may be used concomitantly.

8.2 Verification of Antiplatelet Therapy

After study procedure, in addition to treatment with 2 antiplatelet drugs, optimal treatment with statin or beta-blocker should be conducted. Up to 12 months after the clinical trial procedure, dosing condition of anticoagulant, antiplatelet drug and heart disease related drugs are observed. After one year, dosing condition of only aspirin and antiplatelet drugs are observed.

9. Adverse Events

9.1 Basic Items

In cases of adverse events, appropriate medical care should be provided to subject to ensure safety of subject. By defining procedures for evaluation of adverse events and handling of serious adverse events, organizational control to ensure proper notifications to the sponsor and medical institutions, proper decision on continuation or discontinuation of clinical study and safety of subjects should be established.
In addition, primary investigator or subinvestigator records all adverse events in e-CRF and verifies safety of subject through treatment and laboratory tests conducted as needed even after completion of clinical study, until outcome of adverse event is known.

9.2 Definitions

Adverse events	Adverse Event is any untoward medical occurrence, unintended disease, or untoward clinical signs (abnormal laboratory findings(including abnormal vital signs)) in subjects, which does not necessarily have a causal relationship with study device.
Serious adverse events	Among adverse events, those listed below are considered to be serious adverse events, and serious adverse events that occurred during study period are verified.<br>(1) Fatal<br>(2) Risk of death<br>(3) Hospitalization or prolongation of hospitalization<br>Exception: Hospitalization for test, social hospitalization for convenience of subject, etc.<br>(4) Disability<br>(5) Risk of disability<br>(6) Congenital anomaly<br>(7) Other serious cases in addition to those listed above
Malfunctions	Failure of the study device to perform in accordance with its intended purpose when used in accordance with the instructions for use or clinical investigation protocol. Malfunctions may or may not occur with adverse events.

9.3 Actions to be Taken for Adverse Events

9.3.1 Actions to be Taken in Cases of Occurrence of Adverse Events

9.3.1.1 Actions to be Taken by Primary Investigator in Cases of Adverse Events

When primary investigator or subinvestigator becomes aware of occurrence of adverse event, he/she should promptly provide subject with appropriate treatment and report to the sponsor/In country clinical care-taker as needed.

In addition, treatment and laboratory tests should be conducted as needed to ensure safety of subject. For adverse events (malfunctions) for which causal relationship with the study device cannot be ruled out, treatment and laboratory tests should be conducted as needed to ensure safety of subject even after completion of clinical study until outcome is determined.

9.3.1.2 Actions to be Taken by Primary Investigator in Cases of Serious Adverse Events

When primary investigator or subinvestigator becomes aware of occurrence of serious adverse event, he/she should immediately provide subject with appropriate treatment and promptly report the details to the sponsor/In-country clinical care-taker. In addition, primary investigator reports the details to the sponsor/In-country clinical care-taker in writing. For reporting to head of medical institution in writing, procedures and format specified at medical institution should be followed. If there is no defined format for report at medical institution, the uniform form 14 "Report of Serious Adverse Event and Malfunction" should be used.

In addition, treatment and laboratory tests should be conducted as needed to ensure safety of the subject. In addition, for adverse events that also had a study device malfunction, treatment and laboratory tests should be conducted as needed to ensure safety of subject even after completion of clinical study until outcome is determined.

For malfunction of investigational device without serious adverse event, investigator or subinvestigator shall report the details to the sponsor/in-country clinical care-taker in writing.

In this clinical study, in cases of malfunction of control device, primary investigator, in addition to the procedures described above, promptly reports to Medtronic Japan Co., Ltd. in accordance with procedures defined for handling of (previously approved) medical device.

9.3.1.3 Actions to be Taken by Sponsor/In-country clinical care-taker

The sponsor/In-country clinical care-taker evaluates handling of information, actions to be taken and continuation of clinical study for serious adverse events reported for investigational device by primary investigator. In such cases, the sponsor/In-country clinical care-taker seeks opinion on adverse event concerned from medical expert as needed.

In addition, if serious adverse event reported for investigational device by primary investigator falls under provisions for emergency report, it should be promptly reported to regulatory authority.

9.3.1.4 Provisions on Emergency Report

When an adverse event reported by primary investigator falls under adverse events described in Article 274-2 "Reporting of adverse reactions, etc. of clinical study of Medical Device" applied under Pharmaceuticals and Medical Devices Law, the sponsor/In country clinical care-taker files an emergency report to Pharmaceuticals and Medical Devices

Agency for the adverse event concerned by due dates listed below. Reporting due date is determined using the date of obtainment of information as Day 0.

Predictability	Seriousness	Domestic cases	Overseas cases
Unanticipated	It may lead to death and death	Individual (within 7 days)<br><br>Periodically (every year)	Individual (within 7 days)<br><br>Periodically (every year)
	Other serious	Individual (within 15 days)<br><br>Periodically (every year)	Individual (within 15 days)<br><br>Periodically (every year)
Known	It may lead to death and death	Individual (within 15 days)<br><br>Periodically (every year)	Individual (within 15 days)<br><br>Periodically (every year)
	Other serious	Periodically (every year)	Periodically (every year)
Device Failure	Risk of severe cases and the like are generated	Individual (within 30 days)<br><br>Periodically (every year)	Individual (within 30 days)<br><br>Periodically (every year)

9.4 Assessment of Adverse Events

9.4.1 Record of Adverse Events

For adverse events, the following information should be recorded as detailed as possible.
(1) Name of adverse event
(2) Date of onset
(3) Severity
(4) Seriousness
(5) Treatment
(6) Outcome
(7) Date of outcome
(8) Causal relationship with study procedure
(9) Causal relationship with study device

9.4.2 Severity

Severity of adverse events is classified into 3 grades as described below.

Assessment	Assessment criteria
Mild	There is no issue in performing daily activities.
Moderate	There are issues in performing daily activities.
Severe	Daily activities cannot be performed.

9.4.3 Outcome

For outcome of adverse events, one of the following 7 categories is selected.

Assessment	Assessment criteria
Complete recovery	Complete recovery to conditions before onset of adverse event
Alleviation	Near recovery to conditions before onset of adverse event
Recovery with sequela	Recovery but with sequela
No recovery	Almost no change in adverse event

Assessment	Assessment criteria
Worsening	Increase in severity of adverse event
Death	Death of patient
Unknown	Lost to follow-up before meeting any of the above assessments

9.4.4 Causal Relationship

Causal relationships with study procedure and study device are classified into 5 grades as described below. In addition, adverse events assessed as "Possibly related", "Probably related" and "Clearly related" are considered to be malfunction.

Assessment	Assessment criteria
Not related	Adverse events not related to use of study device at all. Adverse events caused by underlying disease, complication, other medical device or drug
Unlikely related	Those barely related or unrelated to study device chronologically and/or adverse events for which there is more likely cause
Possibly related	Adverse events for which causal relationship between study device and event cannot be ruled out chronologically but correlation is considered to be low and which may be caused by condition of subject or concomitant treatment
Probably related	Adverse events for which causal relationship exists chronologically or which occur less frequently with cessation of use / removal of study device or which cannot be explained rationally with health condition of subject
Clearly related	Adverse events for which causal relationship exists chronologically and which occur less frequently with cessation of use or reoccur with resumption of use

9.5 Operational Check of Study Device during Study Procedure

When operational defect is observed at any time during the study procedure including: visual inspection before insertion of study device into vessel, operational check, approach to target lesion using delivery system, implantation of device or removal of deflated balloon, the primary investigator or subinvestigator, if there is no regulation on report form at medical institution, records necessary information on the uniform form 14 "Report of Serious Adverse Event and Malfunction" and reports to head of medical institution and the sponsor/In country clinical care-taker. When it is possible to retrieve malfunctioned investigational device, study device, along with other procedure equipment (guiding catheter, guide wire, etc.) should also be returned to the sponsor/in country clinical care-taker.

If any operational defect is observed during visual inspection before insertion into blood vessel and during operational check, study device should never be inserted into body. For continuation of treatment of subject, a new study device should be used.

10. Items to Ensure Safety of Clinical Study

10.1 Basic Items

For enrollment of subjects, it should be verified that they meet inclusion criteria and do not meet exclusion criteria before determination of eligibility for enrollment to avoid enrollment of subjects for whom safety cannot be assured.

During clinical study, subjects should be followed carefully for their health status, and efforts should be made to collect and communicate safety information relevant to investigational device or control device.

10.2 Adverse Events Expected for Use of Investigational Device

Expected malfunctions and adverse events of ELX1805J are listed below.

10.2.1 Expected Malfunctions and Adverse Events

Potential malfunctions and adverse events associated with use of investigational device and study procedure include those listed below.

**Adverse events which may be associated with the implantation of a coronary device/stent or PTCA procedure include (in alphabetical order):**
• Abrupt Closure
• Access site complications which may require surgical repair
• Allergic reaction (hypersensitivity to polylactide, polyglycolide, or polycaprolactone based polymers, or platinum, or iridium)
• Aneurysm, pseudoaneurysm
• Angina pectoris
• Arteriovenous fistula
• Cardiac arrest
• Cardiac Arrhythmia
• Cardiac Tamponade
• Cardiac, Pulmonary, Renal failure or insufficiency
• Coronary artery injury, which may require surgical repair or retreatment
• Coronary artery/vessel spasm
• Death
• Dissection, perforation, or rupture of coronary artery
• Drug Reactions to antiplatelet/ anticoagulation agents/contrast media
• Embolism (air, tissue, device or thrombus)
• Emergent or non-emergent CABG
• Failure to deliver the device to the intended site
• Hemorrhage /Hemorrhage complications/ hematoma, including those requiring transfusions
• Hypotension/hypertension
• Infection and pain at insertion site
• Inflammation or infection
• Myocardial ischemia and/or infarction
• Occlusion of the artery
• Pericardial effusion
• Peripheral ischemia
• Pulmonary edema
• Restenosis of treated vessel
• Device migration, embolization, breakage, or misplacement
• Stroke/ Transient Ischemic Attack/cerebrovascular accident
• Thrombosis (acute, subacute, late)

10.3 Adverse Drug Reactions and Adverse Events Related to Study Devices

10.3.1 Adverse Drug Reactions Expected for Zotarolimus

Adverse drug reactions and their frequency of occurrence associated with zotarolimus (API) contained in Resolute Onyx are described in Attached Document 1.

10.3.2 Adverse Drug Reactions Expected for Sirolimus

Sirolimus (API) contained in ELX1805J is a known pharmaceutical active ingredient. Sirolimus is a sufficiently studied and analyzed compound, and has been approved for long-term use in transplant and cancer patients worldwide.

**The following additional side effects/complications may be associated with, but not limited to the use of Sirolimus or Rapamycin-type drugs**
• Acne
• Diarrhea or constipation
• Headache
• Increased blood pressure
• Increased cholesterol or triglyceride levels
• Insomnia
• Nausea /vomiting
• Rash
• Sore or weak muscles or joints
• Tremor
• Upper respiratory or urinary tract infections
• Water retention

10.4 Adverse Drug Reactions Expected for Concomitant Drugs

In this clinical study, to prevent device thrombosis occurring after implantation of study device, antiplatelet therapy using two drugs is given. For both drugs, adverse drug reactions may occur with long-term administration after study procedure. Particularly, with thienopyridine-based antiplatelets, occurrence of serious adverse drug reaction is indicated in the package insert, and there is a warning to conduct blood test every 2 weeks for initial 2 months of administration.
Regarding adverse drug reactions expected with use of concomitant drugs and their frequency of occurrence, refer to the attached document.

10.5 Provision of New Information

When the sponsor obtains new information related to safety of this clinical study, the sponsor promptly notifies head of medical institution and primary investigator in writing. Primary investigator or subinvestigator explains new information to subjects, and primary investigator revises informed consent document as needed.

10.6 Compensation for Health Damage

When any responsibilities for compensation for health damage to subject caused by this clinical study arise, the sponsor/ In Country Clinical Caretaker and medical institution hold discussion to determine handling thereof.

11. Evaluation of Efficacy

11.1 Multicenter randomized single-blind study

11.1.1 Primary Endpoint

The primary endpoint of this clinical study is TLF at 12 months after study procedure. TLF is a hierarchical composite endpoint consisting of the following:

- Cardiovascular death
- MI associated with target vessel (Q-wave or non-Q-wave)
- CI-TLR with PCI or CABG

11.1.2 Secondary Endpoints

(1) Efficacy endpoints

- Acute success rates:
  Lesion success rate, device success rate, procedure success rate

(2) Imaging endpoints

QCA endpoints:

- Acute recoil
- Late lumen loss (in-stent and in-segment) at 12-month follow-up
- Change in vessel angulation from baseline, post-stent and 12-month follow-up
- MLD post-procedure and 12 months
- % DS post-procedure and 12 months

IVUS endpoints:

- Change in mean lumen area from post-procedure to 12-month follow-up
- In-stent % neointimal obstruction at 12-month follow-up
- In-stent late lumen loss at 12-month follow-up
- Acute, persistent and late stent malapposition

OCT endpoints:

- % Strut coverage
- Neointimal thickness
- Vessel Pulsatility - % change in Lumen Area and Device Area during systole and diastole by stationary OCT
- Additional parameters may be assessed

(3) Clinical endpoints:

Measured at 30 days, 6 months, 12 months, 2, 3, 4 and 5 years:

- TLF
- Patient Oriented Clinical Endpoint: Overall cardiovascular outcomes from the patient's perspective. This endpoint is a composite endpoint that includes all-cause mortality (cardiac and non-cardiac), stroke, MI (target vessel and non-target vessel), and revascularization (target vessel and non-target vessel)
- A composite of all-cause mortality, MI (target vessel or non-target vessel), and revascularization (target vessel and non-target vessel)
- Composite of cardiovascular death, target vessel myocardial infarction (TV-MI)*, or clinically-indicated target vessel revascularization (CI-TVR)
- Composite of cardiovascular death, stroke, MI (target vessel and non-target vessel) and revascularization (target vessel and non-target vessel)
- Composite of cardiovascular death, MI (target vessel and non-target vessel) and revascularization (target vessel and non-target vessel)
- CI-TLR
- TLR
- Target vessel revascularization (TVR)
- Clinically Indicated TVR (CI-TVR)
- Revascularization(target vessel and non-target vessel)
- Q-wave MI
- Non Q-wave MI
- MI (target vessel and non-target vessel)
- Target vessel MI
- Cardiovascular death
- All-cause death

- Composite of cardiovascular death or target vessel MI
- Composite of all-cause death or MI (target vessel and non-target vessel)
- Composite of all-cause death, MI (target vessel and non-target vessel), or TVR
- Composite of probable or definite stent thrombosis‡
- Probable stent thrombosis‡
- Definite stent thrombosis‡

** Defined as myocardial infarction not clearly attributed to a non-target vessel*
‡ Defined as per the Academic Research Consortium (ARC-2) criteria

11.1.3 Method for Evaluation of Efficacy

11.1.3.1 Primary Endpoint

Since TLF is a composite rate defined as "cardiovascular death, MI associated with target vessel and performance of CI-TLR", occurrence of these is verified using assessment of angina pectoris, 12-lead ECG, CAG, CK, CK-MB (or troponin), clinical symptoms, observation of adverse events and/or performance of revascularization.

11.1.3.2 Secondary endpoints

(1) Acute success

Device Success rate, Lesion Success rate, and Procedure Success rate between Study group and Control group will be evaluated.

Device success:	% diameter stenosis after implantation of allocated study device in target lesion is less than 30% by QCA (by visual estimation if on-line QCA is unavailable)
Lesion success:	% diameter stenosis after treatment of target lesion with PCI is less than 30% by QCA (by visual estimation if on-line QCA is unavailable)
Procedure success:	Lesion success is achieved for all target lesions, and there is no MACE during hospitalization.

(2) Clinical endpoints:

Measured at 30 days, 6 months, 12 months, 2, 3, 4 and 5 years:

- TLF
- Patient Oriented Clinical Endpoint: Overall cardiovascular outcomes from the patient's perspective. This endpoint is a composite endpoint that includes all-cause mortality (cardiac and non-cardiac), stroke, MI (target vessel and non-target vessel), and revascularization (target vessel and non-target vessel)
- A composite of all-cause mortality, MI (target vessel or non-target vessel), and revascularization (target vessel and non-target vessel)
- Composite of cardiovascular death, target vessel myocardial infarction (TV-MI)*, or clinically-indicated target vessel revascularization (CITVR)
- Composite of cardiovascular death, stroke, MI (target vessel and non-target vessel) and revascularization (target vessel and non-target vessel)
- Composite of cardiovascular death, MI (target vessel and non-target vessel) and revascularization (target vessel and non-target vessel)
- CI-TLR
- TLR
- Target vessel revascularization (TVR)
- Clinically-indicated TVR (CI-TVR)
- Revascularization (target vessel and non-target vessel)
- Q-wave MI
- Non Q-wave MI

- MI (target vessel and non-target vessel)
- Target vessel MI
- Cardiovascular death
- All-cause death
- Composite of Cardiovascular death or target vessel MI
- Composite of all-cause death or MI (target vessel and non-target vessel)
- Composite of all-cause death, MI (target vessel and non-target vessel), or TVR
- Composite of probable or definite stent thrombosis‡
- Probable stent thrombosis‡
- Definite stent thrombosis‡

** Defined as myocardial infarction not clearly attributed to a non-target vessel*
‡ Defined as per the Academic Research Consortium (ARC-2) criteria

11.1.4 Method for Analysis of Endpoints for Efficacy

All endpoints are evaluated for each study device.

<u>11.1.4.1 Primary Endpoint</u>

For TLF (TLF rate at 12 months after study procedure in analysis), non-inferiority of investigational device comparing to control device.

<u>11.1.4.2 Secondary Endpoints</u>

Acute success:
For device success, lesion success and procedure success, each success rate is evaluated.

For clinical endpoints, rate is evaluated at 30 days, 6 months, 12 months, 2, 3, 4 and 5 years for the following:

- TLF
- Patient Oriented Clinical Endpoint: Overall cardiovascular outcomes from the patient's perspective. This endpoint is a composite endpoint that includes all-cause mortality (cardiac and non-cardiac), stroke, MI (target vessel and non-target vessel), and revascularization (target vessel and non-target vessel)
- A composite of all-cause mortality, MI (target vessel or non-target vessel) and revascularization (target vessel and non-target vessel)
- Composite of cardiovascular death, target vessel myocardial infarction (TV-MI)*, or ischemia-driven target vessel revascularization (ID-TVR)
- Composite of cardiovascular death, stroke, MI (target vessel or non-target vessel) and revascularization (target vessel and non-target vessel)
- Composite of cardiovascular death, MI (target vessel or non-target vessel) and revascularization (target vessel and non-target vessel)
- CI-TLR
- TLR
- Target vessel revascularization (TVR)
- Clinically-indicated TVR (CI-TVR)
- Revascularization (target vessel and non-target vessel)
- Q-wave MI
- Non Q-wave MI
- MI (target vessel or non-target vessel)
- Target vessel MI
- Cardiovascular death
- All-cause death
- Composite of Cardiovascular death or target vessel MI
- Composite of all-cause death or MI (target vessel or non-target vessel)

- Composite of all-cause death, MI (target vessel or non-target vessel), or TVR
- Composite of probable or definite stent thrombosis‡
- Probable stent thrombosis‡
- Definite stent thrombosis‡

** Defined as myocardial infarction not clearly attributed to a non-target vessel*
‡ Defined as per the Academic Research Consortium (ARC-2) criteria

11.2 Single-group PK study

11.2.1 Primary Endpoint

Characterization of the pharmacokinetic profile (e.g., Cmax, AUC, $T_{1/2}$)

11.2.2 Secondary endpoints

Secondary endpoints of this study are pharmacokinetic parameters at multiple time points after study procedure

11.3 Record and Timing of Evaluation of Efficacy

All observations and tests related to evaluation of efficacy are recorded in e-CRF. In addition, for timing and acceptable range, see sections 6.1 to 6.2.

12. Evaluation of Safety

12.1 Safety Endpoints

The following endpoints at 1, 6 and 12 months after study procedure and every year thereafter until 5 years after study procedure will be evaluated.

- Device/stent thrombosis rate (for ELX1805J and Resolute Onyx)
- Adverse events, serious adverse events, malfunctions

12.2 Method for Evaluation of Safety

All observations and tests related to evaluation of safety are recorded in e-CRF.

12.2.1 Device/Stent Thrombosis Rate

Device/stent thrombosis occurring after study procedure is checked.

Primary assessment of device/stent thrombosis is conducted using the ARC-2 definition. Thrombosis associated with MI is only evaluated using the per protocol definition of MI. For definition, see (2) Terms of "I. Definition of Terms" (page 7).

12.2.2 Adverse Events, Serious Adverse Events

Adverse events and serious adverse events (see section "9.2 Definitions") occurring during study period are verified.

12.2.3 Malfunctions

Malfunctions (see section "9.2 Definitions") occurring during study period are verified.

12.3 Method for Analysis of Endpoints for Safety

12.3.1 Device/Stent thrombosis Rate

Device/stent thrombosis rate in investigational group and stent thrombosis rated in control device group is reported.

12.3.2 Adverse Events, Serious Adverse Events

Types and rate of adverse events and serious adverse events in both groups are reported.

12.3.3 Malfunctions

Types and rate of malfunctions in both groups are reported.

12.4 Record and Timing of Evaluation of Safety

All test results related to evaluation of safety and adverse events are recorded in e-CRF. In addition, for timing and acceptable range, see sections 6.1 to 6.2.

13. Discontinuation/Suspension of Subject

13.1 Discontinuation Criteria for Subject and Rationale and Procedure for Discontinuation

13.1.1 Discontinuation Criteria

(1) Subject requested discontinuation (withdrawal of consent)

(2) Other cases where primary investigator or subinvestigator determined that study needs to be discontinued

(3) Subject was lost to follow-up*

Even when a subject meets discontinuation criteria, if investigational device is implanted, the subject should be followed up for outcome as much as possible. However, only assessments conducted by time of discontinuation are considered to be a part of clinical study.

* If the subject misses one or more visit, it is counted as a missed visit. Patients should not be considered lost to follow up until the end of the study.

13.1.2 Rationale for Discontinuation Criteria

These criteria were specified for considerations for subjects' right and for safety and ethics in continuation of evaluation of study device.

13.1.3 Procedure for Discontinuation

When subject is found to meet criteria for discontinuation of clinical study after enrollment to this clinical study, primary investigator or subinvestigator notifies the subject of the fact, and assessment of study device is discontinued.
For discontinued subjects, observations and tests should be performed as much as possible at time of discontinuation, and assessment should be conducted at time of discontinuation. In addition, subjects whom study was discontinued due to adverse event should be followed-up until outcome of adverse event is determined as much as possible. When study device is implanted, cause of discontinuation should be followed up as much as possible even after discontinuation.

14. Discontinuation/Suspension of Clinical Study

14.1 Partial Discontinuation/Suspension of Clinical Study

The sponsor/In country clinical care-taker may partially discontinue or suspend clinical study in the following cases. In cases of partial discontinuation or suspension, the sponsor/In country clinical care-taker notifies head of medical institution and primary investigator of discontinuation or suspension.

(1) Review for continuation by institutional review board decided to discontinue/suspend clinical study.

(2) The sponsor/In country clinical care-taker determined to discontinue/suspend clinical study due to serious or continuous violation of GCP or protocol of this clinical study.

(3) The sponsor/In country clinical care-taker determined to discontinue/suspend clinical study for other reasons.

14.2 Discontinuation/Suspension of Entire Clinical Study

The sponsor/In country clinical care-taker may discontinue or suspend clinical study in the following cases. In cases of discontinuation or suspension, the sponsor/In country clinical care-taker notifies head of medical institution and primary investigator of discontinuation or suspension.

(1) The sponsor/In country clinical care-taker determined to discontinue/suspend clinical study due to serious adverse event, etc.

(2) The sponsor/In country clinical care-taker discontinued development of investigational device.

15. Statistical Analysis

15.1 Analysis population

15.1.1 ITT population

The ITT population consists of all randomized patients enrolled in the Bioadaptor RCT study and the first 222 randomized patients enrolled in the European Bioadaptor RCT Study that meet this study’s inclusion/exclusion criteria. Subjects enrolled but not randomized will not be included in the analysis.

15.1.2 Per-Treatment Evaluable population

The population evaluable for treatment (Per-Treatment Evaluable: PTE) consists of subjects that underwent implantation of assigned study device only without any serious deviation from the protocol (implantation in left main trunk, stenting in saphenous veins graft, acute myocardial infarction, etc.).

15.1.3 Subgroup Analysis

Formal assessment with subgroup analysis is not planned. Data of primary endpoint and secondary endpoints may be indicated per the subgroups listed below.

- Gender (male/female)
- Status of diabetes (all diabetes, insulin-dependent diabetes mellitus, non-insulin-dependent, non-diabetes)
- Age (≥ 65 years old vs. <65 years old)
- Single lesion vs. multiple lesions
- Single blood vessel vs. multiple vessels
- 1 study device vs. 2 study devices (excluding bailout)

For all subgroups, if there is any interaction between subgroup and treatment group, it should be reported.

15.2 Method of Statistical Analysis

Details of statistical analysis are defined in statistical analysis plan.

Statistical analysis plan will be prepared by enrollment of the first case.

15.2.1 Efficacy Evaluation

15.2.1.1 Primary Endpoint

TLF rate at 12 months after study procedure

When there are multiple TLF, the primary endpoint, it is counted per case (counted as single incidence).

15.2.1.2 Analysis of Power for Test of Non-Inferiority in TLF at 1 Year, Primary Endpoint

Subjects will be randomly allocated to either investigational device group or control device group at1:1 ratio and enrolled using single-blind, block randomization and subjects are stratified at each medical institution.

The objective of this clinical study is to show non-inferiority. In this non-inferiority study, the primary endpoint is TLF, and this is defined as a hierarchal composite rate consisting of cardiovascular death, MI associated with the target vessel (Q-wave or non-Q-wave), or CI-TLR at 12 months after the study procedure. In order to determine the best estimate (point estimate) of TLF for the Bioadaptor trial, a review of publications and peer-reviewed journal articles to assess the TLF results from DES and drug eluting scaffold clinical trials of marketed devices was completed. Based on these results and proposed inclusion/exclusion criteria of this study, the TLF Rate at 12 months after study procedure is 9.0% for both the ELX1805J arm and the control arm, and non-inferiority margin of δ is 8.6%

The null hypothesis for this study is that the ELX1805J arm will have a 12-month TLF rate that exceeds that of the control device arm by at least a pre-specified margin of δ (delta). The alternative hypothesis is that the ELX1805J arm will have a 12-month TLF rate that is no more than that of the control device, or exceeds that of the control device but by less than δ. Rejection of the null hypothesis will signify that the ELX1805J is *non-inferior* to the control device with regard to 12-month TLF. Specifically, the null hypothesis (Ho) and the alternative hypothesis (Ha) can be expressed as below:

H_o: $\pi_A \geqq \pi_C + \delta$　　　　H_o: $\pi_A \geqq \pi_C + 8.6\%$

H_a: $\pi_A < \pi_C + \delta$　　　　H_a: $\pi_A < \pi_C + 8.6\%$

Here, πA is a true TLF rate of investigational device, and πC is a true TLF rate of control device.
To have a power of 90% in test for non-inferiority for endpoints that follows binomial distribution, a total of 400 patients are required. Tests are conducted using normal approximation of binomial distribution.

Assuming that the rate of lost to follow-up for 12 months after study procedure is 10%, a total number of subjects required is 444 subjects with 222 subjects in investigational device group and 222 subjects in control device group.

15.2.1.3 Analysis of Primary Endpoint

Based on non-inferiority test for primary endpoint that follows binomial distribution, in addition to difference between TLF rates at 12 months for 2 groups (investigational device - control device), one-sided 95% confidence interval of the difference in TLF is calculated. Tests are conducted using normal approximation of binomial distribution. When the upper limit of this confidence interval exceeds 8.6% (delta of non-inferiority), the null hypothesis

is rejected, confirming non-inferiority of investigational device compared to control device for TLF rate at 12 months. This primary analysis will be conducted using the ITT population. In addition, Kaplan-Meier curve for time till first event considered to be TLF is created. If multiple TLF occurred in same case, occurrence date of the first TLF is considered to be the occurrence date of TLF.

15.2.1.4 Analysis of Baseline variables

A table of clinically relevant baseline variables is created, and these variables are compared between subjects allocated to investigational device group and control device group. Categorical variables are tested using appropriate contingency table analysis (normal approximation or chi-square approximation), and continuous variables are tested using unpaired Student's t test or Wilcoxon rank sum test based on distribution of variables. When p-value of one-sided test is less than 0.05, it is considered that there is statistical significance in baseline variable.

15.2.1.5 Analysis of Secondary Endpoints

Time-dependent response variables can be expressed using Kaplan-Meier curve, and differences in these variables between groups are tested using log-rank test. Data for all categorical endpoints (TLF rate at a specific time point, etc.) are shown with number of patients, percentage and Clopper-Pearson's exact 95% confidence interval. Differences between treatment groups are shown using 95% confidence interval. Analyses of secondary endpoints are conducted with the PTE population.

- Acute success: Device success rate, lesion success rate, procedure success rate
- Secondary Endpoint:
  Rates at 30 days, 6 months, 12 months, 2, 3, 4 and 5 years for the following:
  - TLF
  - Patient Oriented Clinical Endpoint: Overall cardiovascular outcomes from the patient's perspective. This endpoint is a composite endpoint that includes all-cause mortality (cardiac and non-cardiac), stroke, MI (target vessel and non-target vessel), and revascularization (target vessel and non-target vessel)
  - A composite of all-cause mortality, MI (target vessel or non-target vessel), and revascularization (target vessel and non-target vessel)
  - Composite of cardiovascular death, target vessel myocardial infarction (TV-MI)*, or clinically-indicated target vessel revascularization (CI-TVR)
  - Composite of cardiovascular death, stroke, MI (target vessel and non-target vessel) and revascularization (target vessel and non-target vessel)
  - Composite of cardiovascular death, MI (target vessel and non-target vessel) and revascularization (target vessel and non-target vessel)
  - CI-TLR
  - TLR
  - Target vessel revascularization (TVR)
  - Clinically-indicated TVR (CI-TVR)
  - Revascularization (target vessel and non-target vessel)
  - Q-wave MI
  - Non Q-wave MI
  - MI (target vessel and non-target vessel)
  - Target vessel MI
  - Cardiovascular death
  - All-cause death
  - Composite of cardiovascular death or target vessel MI
  - Composite of all-cause death or MI (target vessel and non-target vessel)
  - Composite of all-cause death, MI (target vessel and non-target vessel), or TVR

- Composite of probable or definite stent thrombosis‡
- Probable stent thrombosis‡
- Definite stent thrombosis‡

  ** Defined as myocardial infarction not clearly attributed to a non-target vessel*
  ‡ Defined as per the Academic Research Consortium (ARC-2) criteria

15.2.2. Safety Evaluation

15.2.2.1 Safety Endpoint

- Verification of endpoints at 1, 6 and 12 months after study procedure and every year until 5 years after study procedure.Device/stent thrombosis rateAdverse events serious adverse events, malfunctions

  Note:Primary assessment of device/stent thrombosis is conducted using the ARC-2 definition. Thrombosis associated with MI is evaluated using only the per protocol definition of MI. For definition, see (2) Terms of "I. Definition of Terms" (page 7).

15.2.2.2 Analysis of Safety Endpoints

For rate of occurrence of device thrombosis (two-sided 95% confidence interval is determined).

For rates of occurrence of serious adverse events, non-serious adverse events and malfunctions, two-sided 95% confidence interval is determined. Similar summarization is conducted for adverse events for which causal relationship with study device cannot be ruled out.

15.2.3 Analysis of Pharmacokinetic Study

15.2.3.1 Pharmacokinetic Study

Primary endpoint: Pharmacokinetic parameters at 7 days after study procedure (e.g. Cmax, AUC, $T_{1/2}$)

15.2.3.2 Analysis of Pharmacokinetics

To show change in blood concentration of drug at different time points.

- Regarding pharmacokinetic parameters below calculated from blood concentration of drug, descriptive statistics will be calculated.
- Pharmacokinetic parameters at multiple time points after study procedure:
  - e.g., Cmax, AUC, and $T_{1/2}$

Similar summarization is conducted for pharmacokinetics parameters at multiple time points in subjects who were treated with overlapping or multiple devices

15.3 Interim analysis

In this study, application documents for approvals are written with interim report based on data by 12 months after PCI.. No formal interim analyses are planned

16. Deviation from, Change in and Revision of Protocol

16.1 Compliance with Protocol

The sponsor/in country clinical care-taker provides primary investigator with documents and information necessary for evaluation of validity of protocol including the latest investigator's brochure,instructions for use, etc. prior to initiation of clinical study.

Primary investigator discusses and evaluates ethical and scientific validities of contents of protocol and conduct of this clinical study with the sponsor/in country clinical care-taker

based on documents and information provided and agrees on contents of this protocol and compliance with them.

As proof of agreement above, both primary investigator and the sponsor/in country clinical care-taker sign and seal or sign and date on document to representing the official version of protocol.

16.2 Deviation from or Change in Protocol

Primary investigator or subinvestigator may not deviate from or change the protocol without prior written agreement from the sponsor/in country clinical care-taker and prior written approval based on prior review by institutional review board, except for the following cases.

(1) It is medically inevitable to avoid emergent risk to patient, etc.
(2) Changes related only to administrative matters of clinical study

In case of (1) above, primary investigator must submit details and reason of deviation to the sponsor/in country clinical care-taker, head of medical institution and institutional review board as promptly as possible and obtain approval from institutional review board, head of medical institution and the sponsor/in country clinical care-taker in writing.

Primary investigator or subinvestigator records all deviations from protocol.

Primary investigator promptly submits report of all changes in clinical study that may have significant impact on conduct of clinical study or that may increase risk of hazard to patient to the sponsor/in country clinical care-taker, head of medical institution and institutional review board.

16.3 Revisions of Protocol

In the following cases, the sponsor/in country clinical care-taker evaluates continuation of clinical study in accordance with protocol at all medical institutions, some of medical institutions, or each subject and revises protocol as needed.

(1) Information on quality, efficacy and safety of study device or other information important for proper conduct of clinical study was obtained.
(2) It is medically inevitable to revise protocol.
(3) Head of medical institution directed to revise protocol based on recommendation from institutional review board at medical institution.
(
(4) It is necessary to revise major characteristics of analysis (primary endpoint and its analysis) described in protocol.

In cases of decision of the sponsor/in country clinical care-taker to revise or change protocol, primary investigator and sponsor/in country clinical care-taker follows procedures for preparation of protocol.
In other words, the sponsor/in country clinical care-taker thoroughly discusses/evaluates revision with primary investigator, agrees on details of revision and compliance and signs and seals or signs and dates on document to replace protocol as proof. In addition, the sponsor/in country clinical care-taker promptly reports to institutional review board through head of medical institution in writing and obtains approval from institutional review board.

17. Identification of Source Data

17.1 Source Documents

(1) Source documents refer to documents used to generate data to be recorded on eCRF form as results of clinical study.

This includes medical records (e.g., medical record, nurse's record, prescription record, operative note, worksheet for clinical study or medical chart seal), documents which are issued by patient registration center, subject screening list, informed consent form, clinical laboratory test report, ECG chart, film from diagnostic imaging, magnetic media or CD-ROM, study device control record, etc.

(2) All documents and records related to clinical study are stored by primary investigator or medical institution in a manner to allow direct access by the sponsor/in country clinical care-taker and personnel from regulatory authority.

(3) Original copies of informed consent forms are retained in accordance with method specified at medical institution.

(4) Copies of e-CRF in electronic media for their site are retained by primary investigator.

17.2 Items for which Records on eCRF are Considered to be Source Data

Among data recorded on eCRF, records on eCRF are considered to be source data for the following items.

(1) Results of assessment of seriousness and severity of adverse events

(2) Reason for discontinuation

(3) Assessment of causal relationship between adverse event and study device/procedure

17.3 Data not Directly Recorded in eCRF and Considered to be Source Data

Data analyzed by the core laboratory are not directly recorded in eCRF, and they should be considered as source data.

17.4 Collection of eCRF Data

(1) This clinical study is conducted using Electronic Data Capture (EDC).

(2) Subject data are entered directly at medical institution using web browser. These data collected using EDC are considered to be eCRF.

(3) As for quality assurance of eCRF, "Use of Electronic Records and Electronic Signatures in Application for Approvals or Licenses of Medical Products" (PFSB Notification No. 0401022 issued on April 1, 2005) and "Guidance for Electronic Data Capture in Clinical Trials" [issued on November 1, 2007 by Japan Pharmaceutical Manufacturers Association (JPMA) Pharmaceutical Product Assessment Committee] should be followed.

(4) For all subjects consented to participate in this clinical study, eCRF is prepared using the EDC entry screen consistent with items for eCRF data submitted with notification for clinical study.

(5) Primary investigator, subinvestigator or clinical research coordinator prepares eCRF form in accordance with procedures provided by the sponsor/in country clinical care-taker. When clinical research coordinator makes entry, it is conducted under supervision of primary investigator or subinvestigator.

(6) When data are entered in electronic case report from medical institution, predefined logical check is applied automatically. Primary investigator, subinvestigator or clinical research coordinator makes correction as needed.

(7) Comparison against source documents and data review are conducted by the sponsor/in country clinical care-taker for eCRF. When additional inquiry is necessary, the sponsor/in country clinical care-taker issues a manual query in the system, and primary investigator or subinvestigator or clinical research coordinator makes correction of data as needed.
(8) When revision on CRF needed after the first data input is saved in the server system, such information should be recorded in electronically as history of revision and reasons for revision.
(9) Primary investigator signs electronically upon completion of all corrections of eCRF data and verification of accuracy and completeness of correction history and contents. Then, data stored on CD-ROM are verified and stored at medical institution.
(10) Details of data collection are specified in a separate procedures document.

18. Direct Access to Source Documents, etc.

18.1 Direct Access to Source Documents, etc.

In cases of monitoring and audit by the sponsor/in country clinical care-taker and investigation by regulatory authority and institutional review board, primary investigator and medical institution provide direct access to all records related to clinical study including source documents. The sponsor/In country clinical care-taker conducts monitoring and audit at medical institution and directly accesses records related to clinical study including source documents to verify that clinical study is conducted properly and that reliability of data is ensured sufficiently.

Monitoring of cases includes 100% verification of primary endpoint against source document and verification of all informed consent forms for signature, seal and date. In addition, at each medical institution, there will be comparison of data recorded in eCRF against source document is conducted in addition to central monitoring activities.

18.2 Monitoring

The sponsor/in country clinical care-taker takes ethical, legal and scientific responsibilities associated with conduct of this clinical study. In addition, "Procedures for monitoring" is prepared in advance, and monitoring is conducted accordingly. Clinical research associates designated by the sponsor/in country clinical care-taker visit or call medical institution regularly to provide or obtain latest information on clinical study and record their activities.

In addition, the sponsor/in country clinical care-taker may consign entire or parts of monitoring activities to contract research organization.

19. Quality Control and Quality Assurance of Clinical Study

19.1 Items Related to Quality Control of Clinical Study

19.1.1 Major Activities of Monitoring

The sponsor/In country clinical care-taker conducts monitoring to verify that primary investigator or subinvestigator is conducting clinical study in accordance with protocol and laws and regulations related to clinical study. In addition, in accordance with contract with head of medical institution, the sponsor/in country clinical care-taker conducts monitoring of the items listed below and conduct of clinical study as needed and if necessary, requests corrections to primary investigator or medical institution. The sponsor/in country clinical care-taker reserves the right to exclude specific primary investigator or medical institution from clinical study for violation of protocol or regulation.

(1) Verification of status on entry of subjects
(2) Verification of status on obtainment of informed consent from subjects
(3) Verification of status on conduct of clinical study
(4) Verification of status on storage of documents or records related to clinical study
(5) Direct Access to Source Documents, etc.

19.1.2 Provision or Reporting of Information

Primary investigator and head of medical institution provide necessary information to clinical research associate and institutional review board.

Primary investigator reports status on conduct of clinical study to head of medical institution as needed, and head of medical institution receives review for continuation by institutional review board at least once a year.

19.2 Items Related to Quality Assurance of Clinical Study

The sponsor/In country clinical care-taker and head of medical institution clarifies organizational control for conduct of clinical study, and each function conducts their activities in accordance with standard operating procedures.

The sponsor/in country clinical care-taker conducts audits at the sponsor/in country clinical care-taker and medical institution at appropriate timing in accordance with contract with head of medical institution.

Primary investigator and head of medical institution provide necessary information to auditor and official from regulatory authority.

20. Retention of Records

20.1 Retention of Records

Medical institution retains all documents and records related to this clinical study for either one of periods listed below, whichever is longer. However, if the sponsor/In country clinical care-taker need to retain for a period longer than this, method and duration of retention are discussed with the sponsor/in country clinical care-taker.

(1) Date of manufacturing and marketing approval for this investigational device (When it is notified not to attach to application for manufacturing and marketing approval of investigational device, 3 years from date of notification)
(2) 3 years after discontinuation or completion of this clinical study

20.1.1 Notification of Acquisition of Manufacturing and Marketing Approval

When manufacturing and marketing approval is obtained, the sponsor/in country clinical care-taker promptly notifies head of medical institution in writing.

In addition, when there was re-review or reevaluation after manufacturing and marketing approval, the sponsor/in country clinical care-taker notifies head of medical institution of its status.

21. Items Related to Ethics and Compliance with GCP

This clinical study is conducted in accordance with ethical principle based on Declaration of Helsinki, provisions stipulated under Article 14-3, and the "Ministerial Ordinance on Standards for Clinical Study of Medical Device" (MHLW Ministerial Ordinance No. 36) shown below and incorporated into the protocol of this clinical study.
When relevant laws and regulations are revised during this clinical study, revised laws and regulations are followed.

21.1 Institutional Review Board

To conduct this clinical study, it is necessary to obtain approval from institutional review board prior to enrolling subjects.

21.2 Protection of Privacy

The sponsor/in country clinical care-taker use subject identification code for preparation of eCRF to ensure confidentiality of subjects. In addition, for publication of information on clinical study, any information that allows identification of subject may not be included. In addition, the sponsor/in country clinical care-taker does not disclose any information regarding privacy of subjects obtained through this clinical study to third parties.

22. Payment and Insurance

22.1 Payment

The sponsor may provide subjects with financial aid such as aid for transportation expenses, etc. through medical institution in accordance with regulations at each medical institution to help reduce burden on subjects participating in clinical study. If there is no appropriate regulation at medical institution, necessary measures are taken upon discussion between the sponsor and medical institution.

22.2 Compensation for Health Damage and Insurance

The Sponsor will be responsible for reasonable unanticipated emergent patient medical care costs that are a direct result of the evaluable subject’s participation in this Study that are not covered by third-party payers and that are not the result of malpractice, negligence, failure to follow the Protocol or any reckless or willful act or omission by the Investigator, Sub-investigators, Institution or their employees. The Sponsor shall maintain commercially reasonable levels of insurance for the duration of the Study to support its indemnification obligations under this Agreement. Institution shall maintain appropriate insurance coverage (if any) for the duration of the Study at levels sufficient to support its obligations under this Agreement and for damages or claims arising out of any acts of negligence, malpractice or other wrongful actions on the part of Institution, Investigator, Sub-investigators and their trustees, officers and employees, agents, independent contractors and related personnel.

23. Rules for Publication of Results of Clinical Study

At the conclusion of the Biadaptor Study, an abstract reporting the primary results will be prepared and presented in an appropriate international forum. A manuscript will also be prepared for publication in a reputable scientific journal regardless of positive or negative results.

The publication of the principal results from any single center experience within the trial is not allowed until both the preparation and publication of the multi-center results are completed.

All proposed publications and presentation by the Core Lab, Investigators or their personnel resulting from or relating to the Study must be submitted to the Sponsor for review and approval at least 60 days prior to the submission for publication or presentation.

Sponsor will review all materials for proposed publication or presentation for accuracy, confidential information or patentable inventions and the Sponsor may delay any publication or presentation for a reasonable period of time.

All information and data relating to the clinical study, study devices and other study-related data are the sole property of sponsor.

24. Organizational Control for Clinical Study

Organizational control of clinical study is shown in Annex 1, Annex 2 and Annex 3.

25. Study Period

The BIOADAPTOR RCT study will randomize up to 222 total subjects, who will be followed in accordance with the study protocol for 5 years post procedure. It is anticipated that the study duration, from time the first patient is enrolled until the last completes their 5- year follow-up visit, will be approximately 72 months.

Even during the period above, informed consent to subject is stopped when the planned number of subjects for the entire study is reached. Subjects from whom consent is obtained before this may be enrolled into clinical study provided they meet all entry criteria for inclusion into the study.

In this clinical study, subjects are followed up for 5 years after study procedure, but application for manufacturing and marketing approval will be filed using data at 12 months after study procedure. Therefore, if approval is obtained during 5 years of follow-up period, the study is continued as post-marketing study.

26. Reference

[1] Chieffo A, Latib A, Caussin C, et al. A prospective, randomized trial of intravascular-ultrasound guided compared to angiography guided stent implantation in complex coronary lesions: the AVIO trial. Am Heart J. Jan 2013;165(1):65-72.

[2] Fischman DL, Leon MB, Baim DS, Schatz RA, Savage MP, Penn I, Detre K, Veltri L, Ricci, D, Nobuyoshi M et al. A randomized comparison of coronary-stent placement and balloon angioplasty in the treatment of coronary artery disease. Stent Restenosis Study Investigators. *N Engl J Med*. 1994;331:496-501.

[3] Serruys PW, de Jaegere P, Kiemeneij F, Macaya C, Putsch W, Heyndrickx G, Emanuelsson HMarco J, Legrand V, Materne P, et al. A comparison of balloon-expandable-stent implantation with balloon angioplasty in patients with coronary artery disease. BENESTENT Study Group. *N Engl J Med*. 1994;331:449-95.

[4] Kastrati A, Schomig A, Elezi S, et al, Predictive Factors of Restenosis After Coronary Stent Placement. *J Am Coll Cardiol*. 1997;30:1428-36.

[5] Morice MC, Serruys PW, Sousa JE, Fajadet J, Ban Hayashi E, Perin M, Colombo A, Schuler G, Barragan P, Guagliumi G, Molnàr F, Falotico R; RAVEL Study Group. Randomized Study with the Sirolimus-Coated Bx Velocity Balloon-Expandable Stent in the Treatment of Patients with de Novo Native Coronary Artery Lesions. A randomized comparison of a sirolimus-eluting stent with a standard stent for coronary revascularization. *N Engl J Med*. 2002 Jun 6;346 (23):1773-80.

[6] Sousa JE, Costa MA, Abizaid A, et al. Lack of neointimal proliferation after implantation of sirolimus-coated stents in human coronary arteries: a quantitative coronary angiography and three-dimensional intravascular ultrasound study. *Circulation*. 2001;103:192-5.

[7] Stone GW, Ellis SG, Cox DA, et al. One-Year Clinical Results with the Slow-Release, Polymer-Based, Paclitaxel-Eluting TAXUS Stent. *Circulation*. 2004;109: 1942-1947.

[8] Serruys PW, Ong ATL, Piek JJ, et al. A Randomized Comparison of a Durable Polymer Everolimus-eluting Stent with a Bare Metal Coronary Stent: The Spirit First Trial. *EuroInterv*. 2005;1:58-65.

[9] Waltenberger J, et al. Real-world experience with a novel biodegradable polymer sirolimus-eluting stent: twelve-month results of the BIOFLOW-III registry. *EuroIntervention*. 2016;11(10):1106-1110.

[10] Waltenberger, J et.al., BIOFLOW-III an all comers registry with a Sirolimus Eluting Stent, Presentation of Five Year Target Lesion Failure Data. *JACC* 2017;70(18) Suppl B:TCT-737.

[11] https://cardiovascularnews/bioflow-iv-confirms-non-inferiority-of-biotroniks-orsiro-in-japan

[12] von Birgelen C, et al. Very thin strut biodegradable polymer everolimus-eluting and sirolimus-eluting stents versus durable polymer zotarolimus-eluting stents in allcomers with coronary artery disease (BIO-RESORT): a three-arm, randomised, non-inferiority trial. *Lancet*. 2016 Nov 26;388(10060):2607-2617.

[13] de Winter RJ, et.al., A sirolimus-eluting bioabsorbable polymer-coated stent (MiStent) versus an Everolimus-eluting durable polymer stent (Xience) after percutaneous coronary intervention (DESSOLVE III): a randomised, single-blind, multicentre, non-inferiority, phase 3 trial. *The Lancet*. 2018;391(1-11):431-440.

[14] Jain RK, et al. One-year outcomes of a BioMime™ Sirolimus-Eluting Coronary Stent System with a biodegradable polymer in all-comers coronary artery disease patients: The merit-3 study. *Indian Heart J.* 2016;68:599-603.

[15] Iqbal J, Verheye S, Abizaid A, et al. DESyne Novolimus-Eluting Coronary Stent Is Superior to Endeavor Zotarolimus-Eluting Coronary Stent at Five-Year Follow-up: Final Results of the Multicentre EXCELLA II Randomised Controlled Trial. *EuroIntervention*. 2016 Dec;12(11):e1336-e1342.

[16] Costa R. Multi-Center, Randomized Evaluation of the Elixir DESyne® Novolimus Eluting Coronary Stent System with Biodegradable Polymer Compared to a Zotarolimus-Eluting Coronary Stent System: Final 5-Year Results from the EXCELLA BD Study, TCT 2016, poster presentation.

[17] Ziad AA, Serruys PW, Kimura T, Gao R, et al. 2-year outcomes with the Absorb bioresorbable scaffold for treatment of coronary artery disease: a systematic review and meta-analysis of seven randomised trials with an individual patient data substudy. *Lancet*. 2017 Aug 19;390(10096):760-722.

[18] Abizaid A, Costa RA, Schofer J, Ormiston J, Maeng M, Witzenbichler B, Botelho RV, Costa JR, Chamié D, Abizaid AS, Castro JP, Morrison L, Toyloy S, Bhat V, Yan J, Verheye S. Serial Multimodality Imaging and 2-Year Clinical Outcomes of the Novel DESolve Novolimus-Eluting Bioresorbable Coronary Scaffold System for the Treatment of Single De Novo Coronary Lesions. *J Am Coll Cardiol Intv*. 2016;9:565–574.

[19] Cutlip D, Windecker S, Mehran R, et al. Clinical Endpoints in Coronary Stent Trials: A Case for Standardized Definitions. *Circulation*. 2007;115:2344-2351.
